# The effect of Kinesio Taping on motor function in children with cerebral palsy: a systematic review and meta-analysis of randomized controlled trials

**DOI:** 10.3389/fneur.2025.1527308

**Published:** 2025-03-06

**Authors:** Xiaoguang Lin, Jiongliang Zhang, Minmin Wu, Jinting Li, Wenjing Song, Luwen Zhu

**Affiliations:** ^1^Heilongjiang University of Chinese Medicine, Harbin, China; ^2^The Second Affiliated Hospital of Heilongjiang University of Chinese Medicine, Harbin, China

**Keywords:** kinesio taping, cerebral palsy, gross motor function, randomized controlled trials, systematic review, meta-analysis

## Abstract

**Introduction:**

Kinesio taping (KT) is a well-known rehabilitation therapy technique used for treating children with cerebral palsy. However, no meta-analysis of kinesio taping has been conducted specifically for this purpose. This systematic review and meta-analysis aim to explore the effectiveness of kinesio taping in enhancing gross motor function, balance ability, and gait in children with cerebral palsy.

**Methods:**

A comprehensive database search was conducted using PubMed, Embase, the Cochrane Library, Web of Science, Cnki, Wan Fang, VIP, and the Physiotherapy Evidence Database (PEDro) to identify randomized controlled trials (RCTs) investigating the impact of kinesio taping (KT) on cerebral palsy. RCTs published until May 31, 2024, that met our predetermined inclusion and exclusion criteria were included. Data extraction, literature review, and assessment of the methodological quality of the trials were performed. The meta-analysis was conducted using StataSE version 16.

**Results:**

The primary outcome was Gross Motor Function Measure, Berg Balance Scale, Muscle Tension-Heel-Ear Test. The secondary outcomes were step frequency, step speed, step length. Our meta-analysis includes 378 children from 10 RCTs incorporated. Main result the Gross Motor Function Measure (GMFM D) (SMD = 1.00, 95%CI = 0.24–1.77, *p* = 0.01, *I*^2^ = 87.3), the Gross Motor Function Measure (GMFM E) (SMD = 0.84, 95%CI = 0.22–1.46, *p* = 0.008, *I*^2^ = 81.5%), the Berg Balance Scale (BBS) (SMD = 0.81, 95%CI = 0.20–1.42, *p* = 0.009, *I*^2^ = 76.3%). Muscle Tension-Heel-Ear Test (SMD = 1.57, 95%CI = 0.59–2.55, *p* = 0.002, *I*^2^ = 79.8%). The children showed significant improvements in gross motor function, balance and muscle tension compared to the results of the control group. The secondary step length (SMD = 0.46, 95% CI = 0.18–0.76, *I*^2^ = 47.3%, *p* = 0.002) had an improvement effect, but no significant effect on step frequency and step speed.

**Conclusion:**

To some extent, compared to the control group, the addition of kinesio taping improved motor dysfunction in children with cerebral palsy during rehabilitation.

**Systematic review registration:**

https://www.crd.york.ac.uk/PROSPERO/search, identifier: CRD42024528254.

## 1 Introduction

Cerebral palsy (CP) is a well-recognized neurodevelopmental condition that begins in early childhood and persists throughout life (1). Children with CP typically experience motor dysfunction, including muscle weakness and heightened reflexes (2), which can lead to difficulties with posture and coordination, ultimately affecting self-care, mobility, and daily activities (1). Currently, there is no optimal or effective treatment available for improving the gross motor skills and balance functions of children with CP.

According to research conducted by Iona Novak in 2020 (3), substantial clinical trial data support the efficacy of training-based interventions, including action observation training, bimanual training, goal-directed training, and mobility training. Furthermore, several adjunctive interventions, when combined with task-specific motor training, may enhance the positive effects of these training methods. These adjunctive interventions include electrical stimulation, hydrotherapy, taping, transcranial direct current stimulation, and virtual reality serious gaming.

Kinesio tape (KT) is a specialized elastic tape made from latex-free cotton fibers, which has no medicinal properties. It is designed to mimic the elastic properties of muscles, skin, and fascia. When applied correctly, this tape does not restrict soft tissue movement but instead supports weak muscles while allowing for a full range of motion (ROM) (4). KT is believed to stimulate the cutaneous receptors of the peripheral sensorimotor system, which are associated with pain, proprioception, and motor control (5). KT is commonly used for exercise-related injuries, neurological and oncological patients, as well as in pediatric rehabilitation to reduce pain, promote or inhibit muscle activity, prevent injuries, reset joints, assist the lymphatic system, support posture, and enhance body awareness (6, 7).

Although nine systematic reviews have explored the use of KT for children with CP, a comprehensive meta-analysis is still lacking. Existing research suggests that KT may positively influence fine and gross motor abilities, functional independence in activities of daily living (ADL), sitting and standing control, and balance (8). However, evidence indicates that KT is more effective for children with mild to moderate CP and less effective for those with severe CP (9). Furthermore, KT is generally recommended as an adjunct to other rehabilitation techniques, as it may enhance the overall rehabilitation process and improve basic activities of daily living (BADL) (9, 10). Kinesio taping applied to children with spastic diplegia and/or functioning at Gross Motor Function Classification System (GMFCS) (11) levels I and II has moderate evidence (level II) for effectiveness in improving GMF when used as an adjunct to physiotherapy (12). There is strong evidence that KT has a positive effect on sitting and moderate evidence that KT has a positive effect on upper limb function (13, 14). Research has demonstrated that there is insufficient evidence of the effects of KT on other outcomes related to motor function such as gait pattern, gross motor function, balance, and range of motion (14).

Based on these issues, this new meta-analysis aims to evaluate data from RCTs to determine the effectiveness of KT in improving gross motor function, balance, and reducing muscle tension in children with CP. This meta-analysis will provide a more comprehensive understanding of the role of KT in enhancing gross motor function in children with CP, with the goal of offering clearer clinical recommendations.

## 2 Methods and materials

This systematic review and meta-analysis were conducted following the Preferred Reporting Items for Systematic Reviews and Meta-Analyses (PRISMA) guidelines (15). A comprehensive database search was carried out using PubMed, Embase, the Cochrane Library, Web of Science, Cnki, Wan Fang, VIP, and the Physiotherapy Evidence Database (PEDro) (16) to identify RCTs investigating the impact of KT on CP. The study protocol (registration No. CRD42024528254) was registered in PROSPERO.

### 2.1 Search strategy and study selection

A systematic search for articles was conducted from their inception to May 31, 2024. There were no limitations on the language of the articles used for the search. The detailed search strategy is presented in Supplementary Table 1. After removing duplicates, the titles and abstracts of the publications were checked separately by two reviewers (XGL and JLZ). For further evaluation, full-text readings of studies whose relevance could not be determined by title or abstract screening were conducted.

### 2.2 Selection criteria

#### 2.2.1 Inclusion criteria

RCTs were considered eligible, if responding to the questions defined by the following the participants, intervention, comparator and outcome (PICOS) model:

(P) Participants: All participants in the study were children diagnosed with CP by qualified clinicians. None of the participants had received any of the following treatments within the last 6 months or had any of the following conditions: surgical interventions (such as tendon lengthening, peripheral nerve decompression, or partial spinal nerve dissection), botulinum toxin injections, severe circulatory disorders, epilepsy, or intellectual disabilities.

(I) Intervention: The experimental groups included KT and conventional rehabilitation training (Such as gait training, balance training, muscle relaxation training, etc.).

(C) Comparator: Conventional rehabilitation.

(O) Outcome measure: Balance assessed by the Berg Balance Scale (BBS).

(S) All studies included in the analysis were randomized controlled trials (RCTs).

#### 2.2.2 Exclusion criteria

(1) Limitations in motor function due to other diseases, such as stroke or traumatic brain injury. (2) Non RCTs studies, including reviews, conferences, patents, secondary analyses, and case reports. (3) At least 50% of the sample did not have a diagnosis of CP or a risk for CP.

### 2.3 Intervention

All children received conventional CP rehabilitation training, such as exercise therapy, after admission. The experimental group received KT treatment concurrently. The control group only underwent the basic physical rehabilitation therapy mentioned above, which included gait training, balance training, Bobath therapy, proprioceptive neuromuscular facilitation (PNF), muscle relaxation training, etc.

### 2.4 Outcomes

This study reported the clinical results of an assessment of movement, gait, and balance in patients with CP. Primary outcomes included gross motor function, Muscle Tension-Heel-Ear Test, and balance. Stride speed, stride frequency, and stride length in basal gait were considered as secondary outcomes.

### 2.5 Study design

The data analysis included only RCTs that underwent peer review. Review papers, abstracts, protocols, commentaries, or conceptual papers were excluded, except for RCTs.

### 2.6 Data extraction and risk of bias

To identify relevant papers for inclusion, two reviewers (JLZ and XGL) independently examined the abstracts and titles of the articles identified through the searches. The selection process has been meticulously documented to create a PRISMA flow diagram. The two reviewers independently extracted the following data from the selected studies: authors, country, year of publication, age distribution, proportion of men, study design, sample size, intervention conditions, frequency of intervention, and outcome measures. Both reviewers (JLZ and XGL) independently evaluated the methodological quality of the studies using the PEDro scale. Higher scores (ranging from 0 to 10) on the 11-item PEDro scale indicate better methodological quality. To categorize the studies based on quality, the following cut-off points were suggested: excellent (9–10), good (6–8), fair (4–5), and poor (≤3). A third reviewer was consulted to resolve any discrepancies between the two reviewers and make the final decision.

In this part of the analysis, the risk of bias (ROB) for each included study was independently assessed by two authors (XGL and JLZ) using tools from the Cochrane Collaboration (17) covering seven items: random sequence generation, allocation concealment, blinding of participants and personnel, blinding of outcome assessment, incomplete outcome data, selective reporting, and other sources of bias. The ROB for each item was graded as low, unclear or high risk of bias. Discrepancies in quality assessment were resolved through consultation with the third author (MMW). (See Supplementary 7 for ROB criteria) (Supplementary 8 for Risk of bias).

#### 2.6.1 Certainty of evidence assessment

The Grading of Recommendations, Assessment, Development, and Evaluation (GRADE) (18) scale is widely implemented in health technology assessment and guideline development organizations worldwide. Evidence from RCTs starts as high-quality evidence but can be downgraded based on five factors: risk of bias, inconsistency, indirectness, imprecision, and publication bias. The quality of evidence is assessed by two reviewers.

### 2.7 Statistical analysis

The sample data were analyzed using Stata SE version 16.0 (Stata Corp LP, College Station, Texas, USA). Interstudy heterogeneity was assessed using Cochran Q and I^2^ statistics, where significant heterogeneity was defined as *p* < 0.10 and *I*^2^ > 50%.

For outcomes measured on separate scales, the standard mean difference (SMD) was computed. When the studies were reverse scaled (higher values indicated worse outcomes rather than better results), the meaning in each group was multiplied by −1. The estimated *I*^2^ value statistic was used to evaluate statistical heterogeneity. The results of the meta-analyses were graphically represented using forest plots. When there was no significant heterogeneity in the data, it was analyzed using a fixed effects model. Conversely, a random effects model was used in cases with significant group heterogeneity.

### 2.8 Publication bias and sensitivity analysis

To assess the potential of publication bias in the current sample, if the number of outcome indicators in RCTs does not reach 10, a publication bias test would not be carried out using a funnel plot; instead, the Egger’s test (19) method was adopted. Subgroups were analyzed according to the number of weeks of intervention included in the experiment, divided into 4, 8, 10, and 12 weeks. Subgroups were also analyzed based on the site of the patch, categorized as patch site below the hip and patch site above the hip.

### 2.9 Assessment of heterogeneity and reporting bias

Heterogeneity was assessed using the Higgins test by calculating *I*^2^, and values of *I*^2^ greater than 50% could indicate significant heterogeneity. If there were more than 10 eligible studies, funnel plots were utilized to assess publication bias. At the same time, Egger’s tests were employed to verify the presence of publication bias.

## 3 Results

### 3.1 Search result

**Figure 1:**
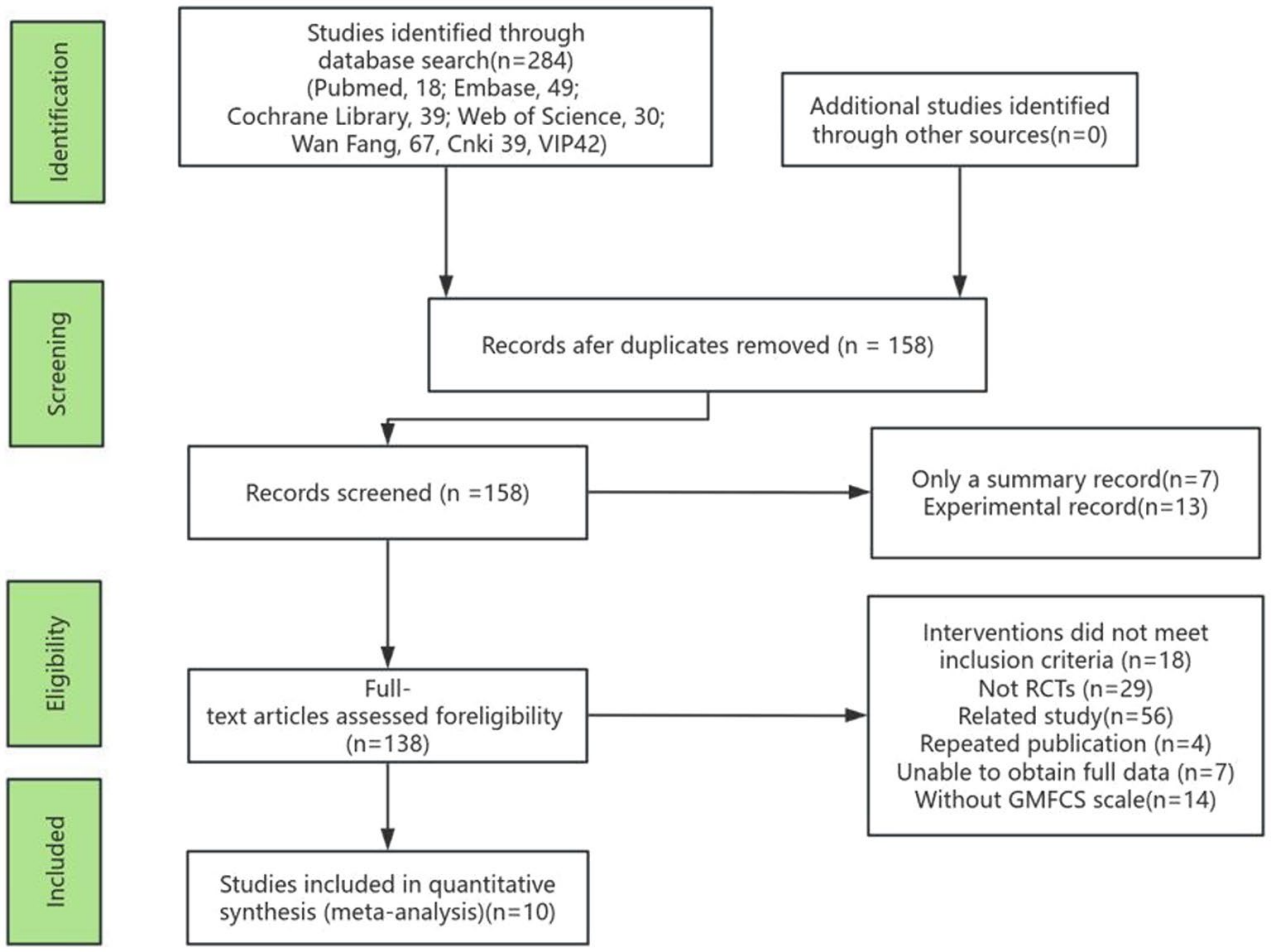
The study selection process.

From the seven databases used in the initial search, 284 potentially relevant studies were identified (PubMed, *n* = 18; Embase, *n* = 49; Cochrane Library, *n* = 39; Web of Science, *n* = 30; Wan Fang, *n* = 67; CNKI, *n* = 39; VIP, *n* = 42). After excluding duplicates, 158 articles were screened based on title and abstract for inclusion in the full-text review. Ultimately, 138 studies passed this stage. Subsequently, 10 studies (20–29) were included in the meta-analysis after excluding 128 studies. The included studies focused on Gross Motor Function D-area (GMFM-D) (30), Gross Motor Function E-area (GMFM-E), Berg Balance Scale (BBS) (31), flexibility, Muscle Tension-Heel-Ear Test (Muscle Tension), step length, step speed, and step frequency. The PRISMA flowchart illustrates the study selection process (Figure 1).

### 3.2 Study characteristics

Detailed characteristics of the included studies are presented in Table 1.

**Table 1:** The basic characteristics of included studies.

Study	Country	Design	EG					CG					
			Sample size	Age year	Male%	Intervention	Time, frequency	Sample size	Age year	Male%	Intervention	Time, frequency	Outcome
Kemer et al. (20)	Turkey	RCT	11	11 ± 2.22	36.4	KT group physical therapy +CG therapy	A total of 8 sessions, per week 2 sessions, for 4 weeks, per session 3 day.	9	9 ± 4.44	66.7	Including stretching, loading, functional stretching, gait mode promotion and electrotherapy.	A total of 8 sessions, per week 2 sessions, for 4 weeks, per session 40 min.	GMFM D GMFM E Balance step frequency
Kaya Kara et al. (21)	Turkey	RCT	15	7.5 ± 3.33	53.3	KT group physical therapy +CG therapy	A total of 24 sessions, per week 2 sessions, for 12 weeks, per session 3 day.	15	7.2 ± 4.96	46.6	Conventional treatment, stretching, loading, functional stretching, walking.	Per week 2 sessions, for 12 weeks.	GMFM D GMFM E
Şimşek et al. (22)	Turkey	RCT	15	8.27 ± 3.43	53.3	KT group physical therapy +CG therapy	A total of 24 sessions, per week 2 sessions, for 12 weeks, per session 3 day.	15	6.87 ± 2.10	66.7	Physical therapy: upper limb activities + grasping + sitting + balance reaction	A total of 36 sessions, per week 3 sessions, for 12 weeks, per session 1 h.	GMFM
Abdel Ghafar et al. (23)	Egypt	RCT	13	12.08 ± 2.56	61.5	KT group physical therapy +CG therapy	A total of 28sessions, per week 7sessions, for 4 weeks, per session 1 day	13	11.85 ± 2.27	58.3	Traditional physical therapy.	A total of 12 sessions, per week 3 sessions, for 4 weeks, per session 1 h.	Stride length, Cadence step, Walking velocity, Step length
Shi et al. (25)	China	RCT	37	3.00 ± 0.90	70.3	KT group physical therapy +CG therapy	A total of 24 sessions, per week 2 sessions, for 12 weeks, per session 1-2 day.	37	3.36 ± 0.91	67.6	Standing, walking, walking, Pelvic control, cross -obstacle and other training. Feeling training massage and massage training	A total of 96 sessions, per week 12 sessions, for 8 weeks, per session 30 min.	GMFM D, GMFM E, BBS, Step length, step speed
Xu et al. (26)	China	RCT	25	5.62 ± 1.75	48	KT group physical therapy +CG therapy	A total of 24sessions, per week 3 sessions, for 8 weeks, per session 8 h.	25	5.57 ± 1.69	56	Bobath + PNF	A total of 96 sessions, per week12sessions, for 8 weeks, per sessions 45 min.	GMFM A-E, step length, step speed, step frequency
Cheng and Zhao (27)	China	RCT	20	5.75 ± 2.22	60	KT group physical therapy +CG therapy	A total of 24 sessions, per week 2 sessions, for 12 weeks, per session 48 h.	20	5.68 ± 2.25	85	Sports therapy, homework Treatment, massage treatment, physical factor therapy	NA	Step length, step speed, stride length, stride width, BBS, GMFM
Wang et al. (28)	China	RCT	29	3.02 ± 1.02	51.7	KT group physical therapy +CG therapy	For 12 weeks, per session 1-2 day.	29	2.86 ± 0.91	58.6	Passive activity, muscle training	A total of 120 sessions, per week 10 sessions, for 12 weeks, per sessions 40 min.	GMFMD GMFME
Li et al. (29)	China	RCT	15	1.97 ± 0.58	46.7	KT group physical therapy +CG therapy	A total of 40 sessions, per week 4 sessions, for 10 weeks, per session 1 day	15	2.34 ± 0.58	60	Bobath therapy technology, stretching, physical weight training, balance training, walking exercise training, etc.	A total of 40 sessions, per week 4 sessions, for 10 weeks, per session 30 min.	GMFMD GMFME BBS
Zhou et al. (24)	China	RCT	10	1.97 ± 0.58	50	KT group physical therapy +CG therapy	A total of 40 sessions, per week 5 sessions, for 8 weeks, per session 1 day	10	2.25 ± 0.58	40	Conventional exercise treatment	A total of 40 sessions, per week 5 sessions, for 8 weeks, per session 40 min.	GMFM Muscle tension

GMFM D, gross motor function measure D; GMFM E, gross motor function measure E; BBS, berg balance scale; Muscle tension; Step length; Step speed; Step frequency.

The total sample size was 378, and all included children with CP were compared under a basic physiotherapy intervention. The control group received only physical therapy, whereas the experimental group received both physical therapy and KT intervention. 190 were assigned to the intervention group and 188 to the control group. There were 217 men (58%) and 156 women (42%) in the sample, with each trial containing 20 to 74 participants. The average age of the experimental group ranged from 1.97 to 12.08, while the control group ranged from 2.25 to 11.85. The main results of subgroup analysis on gross motor function, balance, and gait from the included studies can be found in Table 2.

**Table 2:** Results of the analysis of individual outcome indicators and their subgroups.

Outcomes		Group	Number of comparison	Total number of participants		Effect Size			Heterogeneity
					WMD/SMD	95%CI		*p*	*I*² (%)
		Over analysis	6	262	SMD = 1.00	[0.24, 1.77]		0.010	87.30%
GMFM−D		4 Weeks	1	20	SMD = 1.32	[0.34, 2.31]		0.008	NA
	Subgroup	8 Weeks	2	124	SMD = 0.66	[0.29, 1.02]		0.000	0.00%
		10 Weeks	1	30	SMD = 0.47	[−0.26, 1.20]		0.204	NA
		12 Weeks	2	88	SMD = 1.47	[−1.41, 4.35]		0.318	96.80%
		Over analysis	6	262	SMD = 0.84	[0.22, 1.46]		0.008	81.40%
		4 Weeks	1	20	SMD = 1.33	[0.39, 2.26]		0.005	NA
GMFM−E	Subgroup	8 Weeks	2	124	SMD = 0.64	[0.28, 1.00]		0.001	0.00%
		10 Weeks	1	30	SMD = −0.13	[−0.85, 0.59]		0.725	NA
		12 Weeks	2	88	SMD = 1.32	[−0.43, 3.06]		0.139	92.20%
		Over analysis	5	214	SMD = 0.81	[0.20, 1.42]		0.009	76.30%
		4 Weeks	1	20	SMD = 1.74	[0.69, 2.79]		0.001	63.20%
BBS	Subgroup	8 Weeks	2	124	SMD = 1.14	[0.49, 1.78]		0.001	NA
		10 Weeks	1	30	SMD = 0.24	[−0.48, 0.96]		0.516	NA
		12 Weeks	1	40	SMD = −0.02	[−0.64, 0.60]		0.952	NA
		Over analysis	3	124	SMD = 1.57	[0.59, 2.55]		0.002	79.80%
Muscle tension	Subgroup	8 Weeks	2	94	SMD = 1.81	[0.47, 3.14]		0.008	82.40%
		10 Weeks	1	30	SMD = 1.07	[0.30, 1.84]		0.006	NA
		Over analysis	4	189	SMD = 0.47	[0.18, 0.76]		0.002	47.30%
Step length	Subgroup	4 Weeks	1	25	SMD = 0.30	[−0.49, 1.09]		0.452	NA
		8 Weeks	2	124	SMD = 0.69	[0.32, 1.05]		0.000	30.50%
		12 Weeks	1	40	SMD = −0.05	[−0.67, 0.57]	0.873		NA
		Over analysis	4	189	SMD = 0.38	[−0.14, 0.90]	0.157		66.60%
Step speed	Subgroup	4 Weeks	1	25	SMD = 0.08	[−0.71, 0.86]	0.849		NA
		8 Weeks	2	124	SMD = 0.68	[−0.24, 1.59]	0.146		82.90%
		12 Weeks	1	40	SMD = −0.01	[−0.63, 0.61]	0.986		NA
Stride frequency	Subgroup	Over analysis	4	135	SMD = 0.16	[−0.19, 0.50]	0.373		25.50%
		4 Weeks	2	45	SMD = 0.46	[−0.14, 1.06]	0.135		61.30%
		8 Weeks	1	50	SMD = 0.01	[−0.54, 0.57]	0.966		NA
		12 Weeks	1	40	SMD = 0.01	[−0.61, 0.63]	0.968		NA
GMFM−D		Over analysis	6	262	SMD = 1.00	[0.24, 1.77]	0.010		87.30%
	Subgroup	Below the hip	4	192	SMD = 0.97	[−0.19, 2.13]	0.102		92.20%
		Others	2	70	SMD = 1.00	[0.50, 1.50]	0.000		0.00%
		Over analysis	6	262	SMD = 0.84	[0.22, 1.46]	0.008		81.40%
GMFM−E	Subgroup	Below the hip	4	192	SMD = 0.78	[−0.14, 1.70]	0.098		88.30%
		Others	2	70	SMD = 0.86	[0.34, 1.38]	0.001		5.80%
		Over analysis	5	214	SMD = 0.81	[0.20, 1.42]	0.009		76.30%
BBS	Subgroup	Below the hip	3	144	SMD = 0.39	[−0.16, 0.94]	0.166		60.60%
		Others	2	70	SMD = 1.57	[1.03, 2.11]	0.000		0.00%
Muscle tension	Subgroup	Over analysis	3	124	SMD = 1.57	[0.59, 2.55]	0.002		79.80%
		Below the hip	2	104	SMD = 1.78	[0.44, 3.12]	0.009		86.60%
		Others	1	20	SMD = 1.07	[0.13, 2.02]	0.026		NA
Step length	Subgroup	Over analysis	4	189	SMD = 0.47	[0.18, 0.76]	0.002		47.30%
		Below the hip	3	139	SMD = 0.31	[−0.03, 0.64]	0.072		1.40%
		Others	1	50	SMD = 0.97	[0.38, 1.56]	0.001		NA
Step speed	Subgroup	Over analysis	4	189	SMD = 0.38	[−0.14, 0.90]	0.157		66.60%
		Below the hip	3	139	SMD = 0.14	[−0.20, 0.47]	0.426		0.00%
		Others	1	50	SMD = 1.16	[0.56, 1.76]	0.000		NA
Stride frequency	Subgroup	Over analysis	4	135	SMD = 0.16	[−0.19, 0.50]	0.373		25.50%
		Below the hip	2	65	SMD = 0.03	[−0.46, 0.51]	0.914		0.00%
		Others	2	70	SMD = 0.28	[−0.20, 0.76]	0.253		71.40%

GMFM D, gross motor function measure D; GMFM E, gross motor function measure E; BBS, berg balance scale; Muscle tension; Step length; Step speed; Step frequency; Weighted Mean Difference (WMD), Confidence Interval (CI), Standard Mean Difference (SMD).

### 3.3 Quality assessment

Using the Physiotherapy Evidence Database (PEDro) Scale to assess the risk of bias, the average score on the PEDro scale was 6, with grades ranging from 5 to 7. Except for one study (21), which was deemed to be of good quality, nine studies (20, 22–29) were deemed to be of fair quality (Table 3). All measurements were assessed using GRADE scoring, indicating low certainty (Table 4).

**Table 3:** The methodological quality included studies on the PEDro scale.

Study	Items of PEDro scale											Total scores	Quality
	1	2	3	4	5	6	7	8	9	10	11		
Seda Nur Kemer et al.	Yes	1	0	1	0	0	0	0	0	1	1	4	Fair
Ozgun et al.	Yes	1	0	1	0	0	1	0	1	1	1	6	Good
T Ulay et al.	Yes	1	0	1	0	0	0	0	1	1	1	5	Fair
Mohamed et al.	Yes	1	0	1	0	0	1	0	0	1	1	5	Fair
Zhou Wen Ping et al.	Yes	1	0	1	0	0	0	0	1	1	1	5	Fair
Shi Jinli et al.	Yes	1	0	1	0	0	0	0	1	1	1	5	Fair
Xu et al.	Yes	1	0	1	0	0	0	0	1	1	1	5	Fair
Cheng et al.	Yes	1	0	1	0	0	0	0	1	1	1	5	Fair
Wang et al.	Yes	1	0	1	0	0	0	0	1	1	1	5	Fair
Li et al.	Yes	1	0	1	0	0	0	0	1	1	1	5	Fair

Items of the PEDro scale: 1, specified eligibility criteria (yes/no); 2, random allocation; 3, concealed allocation; 4, comparability at baseline; 5, blinded subjects; 6, blinded therapists; 7, blinded assessors; 8, sufficient follow-up; 9, intention-to-treat analysis 10, comparison between groups; 11, point estimates and variability. For terms 2–11:1, the corresponding criterion is satisfied; 0, the criterion is not satisfied. The following cut-off points were suggested: excellent 182 (9–10), good (6–8), fair (4–5), and poor (≤3).

**Table 4:** Quality of evidence.

Outcomes	Study design	Risk of bias	Inconsistency	Indirectness	Imprecision	Publication bias	Other consideration	Numbers of participants		Absolute effect	Certainty
								EG	CG	SMD (95% CI)	
*GMFM-D*	RCTs	serious	no	no	no	no	no	132	130	1.00 (0.24,1.77)	Low
GMFM-E	RCTs	serious	no	no	no	no	no	132	130	0.84 (0.22,1.46)	Low
Berg	RCTs	serious	serious	no	no	no	no	108	106	0.81 (0.20,1.42)	Low
Muscle Tension	RCTs	serious	no	no	serious	no	no	62	62	1.57 (0.59, 2.55)	Low
Step frequency	RCTs	serious	no	no	serious	no	no	69	67	0.16 (−0.19,0.50)	Low
Step speed	RCTs	serious	no	no	serious	no	no	95	94	0.08 (−0.71,0.86)	Low
Step length	RCTs	serious	no	no	serious	no	no	95	94	0.46 (0.18,0.76)	Low

EG, Experimental group; CG, Control group; CI, Confidence interval; GMFM, Gross motor function measure; BBS, Berg Balance Scale; SMD, Standard Mean Difference.

### 3.4 Outcome statistics

Among the 10 studies included, 6 studies focused on the gross movement of the lower limbs (20, 21, 25, 27–29), 5 studies (20, 25–27, 29) were related to the BBS (balance), and 4 studies included information on step frequency, step speed, and step length (23, 25–28). The detailed results are presented in Table 2.

### 3.5 Outcomes analysis

#### 3.5.1 GMFM-D

After analysis, the effectiveness of KT on the gross motor function scale D area of children with CP is significant. Six studies (20, 21, 25, 26, 28, 29) reported on the GMFM-D. The difference between the experimental group and the control group was statistically significant (SMD = 1.00, 95% CI = 0.24–1.77, *p* = 0.01, *I*^2^ = 87.3%) (Supplementary 1, Figure A). There was no evidence of publication bias (P for Egger’s test = 0.691) (Supplementary 4, Figure 1). The certainty of this evidence was low. After sensitivity analysis, excluding the study by (28), *I*^2^ dropped to 54.6%, the difference between the experimental group and the control group was statistically significant (*p* = 0.001), which resulted in a stable and reliable outcome (Supplementary 5, Figure 1).

#### 3.5.2 GMFM-E

The gross motor function table E area assesses the scoring, running, and jumping abilities of children with CP. After analyzing the data, the effectiveness of KT on the gross motor function scale E area of children with CP is significant. Six studies (20, 21, 25, 26, 28, 29) reported on the GMFM-E. The difference in control was statistically significant (SMD = 0.84, 95% CI = 0.22–1.46, *p* = 0.008, *I*^2^ = 81.5%) (Supplementary 1, Figure B). Egger’s test (*p* = 0.982 > 0.05) showed no evidence of publication bias (Supplementary 4, Figure 2). The certainty of this evidence was low. After a sensitivity analysis, excluding the study by (28), *I*^2^ dropped to 46.8%, the difference between the experimental group and the control group was statistically significant (*p* = 0.004), resulting in a stable and reliable outcome (Supplementary 5, Figure 2).

#### 3.5.3 Berg Balance Scale

Berg Balance Scale was developed to measure balance in elderly individuals. Nowadays, it is also used to assess balance in children with balance dysfunction.

After the analysis, KT had significant results in the balance function of the intervention effect of children with CP. The research included in this scale included (20, 25–28). The difference between the experimental group and the control group was statistically significant (SMD = 0.81, 95% CI = 0.20–1.42, *p* = 0.009, *I*^2^ = 76.3%) (Supplementary 1, Figure C). Through Egger’s test (*p* = 0.709 > 0.05) (Supplementary 4, Figure 3), there was no publication bias. Excluding the low evidence (27), *I*^2^ reduced by 9.2%, the difference between the experimental group and the control group was statistically significant (*p* = 0.001) (Supplementary 5, Figure 3).

#### 3.5.4 Muscle tension-heel-ear test

The muscle tension assessment, including the heel-ear test, is a stretching test used to determine muscle tension as outlined in China’s CP rehabilitation guidelines (2015) (32). Three studies (24, 25, 29) reported on muscle tension, and the results were statistically significant (SMD = 1.57, 95% CI = 0.59–2.55, *I*^2^ = 79.80%, *p* = 0.002) (Supplementary 1, Figure D). The quality of this evidence was low; after eliminating (25), *I*^2^ reduced to 79.8, the difference between the experimental group and the control group was statistically significant (*p* = 0.000), resulting in a steady outcome (Supplementary 5, Figure 4). There was no evidence of publication bias (P for Egger’s test = 0.307) (Supplementary 4, Figure 4).

#### 3.5.5 Step speed

Step speed refers to the walking speed of children with CP. The research included in (23, 25–27) (SMD = 0.38, 95% CI = −0.14-0.90, *I*^2^ = 66.6%, *p* = 0.157) (Supplementary 1, Figure E) provided evidence supporting step speed but was deemed to have low certainty. After eliminating low-quality research (26), *I*^2^ reduced by 66.6%, the difference between the experimental group and the control group was not statistically significant (*p* = 0.426), resulting in stable results (Supplementary 5, Figure 5). There was no evidence of publication bias (P for Egger’s test = 0.986) (Supplementary 4, Figure 5).

#### 3.5.6 Step length

Step length refers to the one-step length of children with CP. The research included in the same (23, 25–27) showed a standardized mean difference (SMD = 0.46, 95% CI = 0.18–0.76, *I*^2^ = 47.3%, *p* = 0.002) (Supplementary 1, Figure F). The result was statistically significant. After eliminating low-quality research (26), *I*^2^ reduced by 45.9%, the difference between the experimental group and the control group was not statistically significant (*p* = 0.075), yielding stable results (Supplementary 5, Figure 6). Through Egger’s test, there was no evidence of publication bias (P for Egger’s test = 0.729) (Supplementary 4, Figure 6).

#### 3.5.7 Step frequency

Step frequency refers to the rhythm of steps in children with CP. Four studies (20, 23, 26, 27) reported on the step frequency. The evidence supporting step frequency was deemed to have low certainty (SMD = 0.16, 95% CI = −0.19 to 0.50, *I*^2^ = 25.5%, *p* = 0.373) (Supplementary 1, Figure G). After eliminating low-quality research (20), *I*^2^ reduced by 25.5%, the difference between the experimental group and the control group was not statistically significant (*p* = 0.913), which yielded consistent results (Supplementary 5, Figure 7). There was no evidence of publication bias (P for Egger’s test = 0.182) (Supplementary 4, Figure 7).

### 3.6 Subgroup analysis

We performed subgroup analyses for three categorical indicators: (A) Different weeks of intervention. (B) Different adhesion areas. Specific results are detailed in Table 2.

#### 3.6.1 Subgroup analysis of GMFM-D

In the intervention weeks, data analysis was conducted in groups. The study of 8 weeks (25, 26) (SMD = 0.66, 95% CI = 0.29–1.02, *I*^2^ = 0.0%, *p* = 0.000) showed a significant reduction (Supplementary 2, Figure 1).

Grouping based on the site of adhesion showed that the patch site above the hip had a standardized mean difference (SMD = 1.00, 95% CI = 0.50–1.50, *I*^2^ = 0%, *p* = 0.000), indicating a statistically significant difference (Supplementary 3, Figure 1).

#### 3.6.2 Subgroup analysis of GMFM-E

The study by (25, 26) (SMD = 0.64, 95% CI = 0.28–1.00, *I*^2^ = 0%, *p* = 0.001) on the 8-week intervention showed a significant decrease in heterogeneity and statistical significance (Supplementary 2, Figure 2).

Patch site above the hip (20, 26): (SMD = 0.86, 95% CI = 0.34–1.38, *I*^2^ = 5.8%, *p* = 0.001), which revealed a statistically significant difference (Supplementary 3, Figure 2).

#### 3.6.3 Subgroup analysis of Berg Balance Scale

In the number of intervention weeks, the data analysis was performed in groups. The study by (25, 26) (SMD = 1.50, 95% CI = 0.87–2.13, *I*^2^ = 63.2%, *p* = 0.001) of the intervention over 8 weeks showed heterogeneity and statistical significance (Supplementary 2, Figure 3).

Grouping based on the site of adhesion, the patch site below the hip (25, 27, 29): (SMD = 0.39, 95% CI = −0.16-0.94, *I*^2^ = 60.6%, *p* = 0.166). The result was not statistically significant. The patch site above the hip (20, 26): (SMD = 1.57, 95% CI = 1.03–2.11, *I*^2^ = 0%, *p* = 0.000), which revealed a statistically significant difference (Supplementary 3, Figure 3).

#### 3.6.4 Subgroup analysis of step length

In the intervention weeks, a group analysis of data was conducted, and after 8 weeks of intervention research (25, 26) (SMD = 0.69, 95% CI = 0.32–1.05, *I*^2^ = 30.5%, *p* = 0.000), heterogeneity decreased significantly and became statistically significant.

A treatment period of 8 weeks was significantly more effective than both short and long treatment courses in improving gross motor skills, balance function, and step length. In the subgroup where KT was applied, the improvement in gross motor skills and balance function was notably higher in the adhesion site above the hip compared to below the hip. However, there were no significant effects observed in terms of muscle tension and gait.

### 3.7 Sensitivity analysis

Sensitivity analysis of the data was performed using Stata SE version 16.0. The results suggested that the data sensitivity in each group was generally stable. The results of the sensitivity analysis of GMFM D GMFM E suggested that (28) was the main source of heterogeneity. It is assumed that this is because the experimental observation group took 20 min out of each exercise therapy session to receive training specifically designed to improve motor control, in addition to the regular exercise therapy and intramuscular patch treatment. Supplementary 5 presents the outcomes of the sensitivity analysis.

## 4 Discussion

### 4.1 Main finding

The purpose of this meta-analysis is to evaluate the effects of KT when used in combination with motion training for children with varying degrees of motor function impairment. This is the first meta-analysis of KT on the gross motor function of children with CP. The results of this study demonstrate the effects of KT on children with CP. Based on the inclusion of 10 studies, it was found that KT combined with exercise training improves the gross movement function of children with CP. However, there was no significant improvement in step speed and step frequency. The quality assessment using the PEDro scale revealed only one study with slightly good quality. Table 4 indicates low certainty of evidence and high risk of bias for outcomes. Through sensitivity analysis, a stable and reliable result was obtained, except for balance force. In conclusion, the intervention of KT combined with exercise training is beneficial for children with CP.

Investigators have stated the goals of taping children with CP as being to correct postural misalignment, enhance joint stability, activate weak muscles, support weak structures, manage spasticity, and stimulate the sensory system. The latest 2024 systematic review (8) suggests that the use of KT has beneficial effects on upper extremity (UE) function, including range of motion (ROM), gross and fine motor function, grip strength, spasticity, and manual dexterity, in both children and adolescents with CP. This is consistent with some of our results. However, further research is needed to reinforce the conclusions on the efficacy of KT as a therapeutic tool.

In the study by (24, 26), and (29), they performed spinal taping to promote dorsiflexion stability. We hypothesized that spinal taping is essential for increasing stability in children, but more clinical trials are needed to validate this idea further.

### 4.2 Analysis of sources of heterogeneity

#### 4.2.1 GMFM D and GMFM E

After excluding the literature one by one through sensitivity analyses, it was found that the heterogeneity decreased significantly after the exclusion of study (28), and the heterogeneity of GMFM D results remained. The subgroup heterogeneity present in study (28) was found to be extremely high by subgroup analysis, both in subgroup analyses with different paste sites and in subgroup analyses with different weeks of intervention. This is consistent with our sensitivity analyses. By reading the full text of the analysis, it was hypothesized that the heterogeneity could be due to the fact that the observation group in this study, in addition to the regular exercise therapy and intramuscular patch treatment, took 20 min out of each exercise therapy session to receive training specifically designed to improve motor control, including training for step speed, step length and step width. The analysis of the sources of heterogeneity in this result also applied to the sources of heterogeneity in GMFM E. The results of the exclusion studies (28) were found to be significant. Heterogeneity was found to decrease significantly after the exclusion of the study (28). It is hypothesized that the source of heterogeneity is the same for this study.

#### 4.2.2 BBS

After excluding study (27) by sensitivity analysis, the model was found to be stable with insignificant changes in heterogeneity. Subgroup analyses were performed and found that the subgroups in which study (25) was located retained a high degree of heterogeneity across the subgroups of studies in terms of paste site and weeks of intervention. By reading the full article, it was hypothesized that the source of heterogeneity could be due to the addition of sensory and electrical stimulation training to the control group-based rehabilitation.

As for the balance function, KT may improve the balance function of children through body stimulation. A study (20) speculates that internal effects on the balance function should focus more on the activation of the gluteal muscles. By stimulating the body and activating the gluteal muscles, the pelvis and hips of the children can be promoted on the front and sagittal surfaces. The symmetry and stability of the bones can better improve the balance parameters, as mentioned in (21), where children are prone to abnormal standing balance ability and abnormal standing position control. Due to the relatively high trunk tension in children and insufficient core strength, clinics need to implement KT therapy to promote the contraction of the erector spinae muscle, relieve the abnormal muscle tension of the child, and achieve the goal of promoting the reconstruction of the child’s muscle function.

#### 4.2.3 Muscle tension-heel-ear test

To explore the source of consistency, heterogeneity decreased from 79.8% to 0 after excluding the study by sensitivity analysis (25). By reading the full article, we hypothesized that that the heterogeneity originated from the paste-up site in the study, which wrapped around the ankle site, making the heterogeneity in the analysis of the dystonia results too large. Due to the limited amount of research included, after the analysis of subgroups, the cause of high heterogeneity can be determined. After removing study (25), a reliable result was obtained. Interestingly, in studies (25, 29), the muscle tension of children with spasm-type CP was reduced by intervention in the knee joint and calf gastrocnemius muscle. Study (24) intervened through the spinal muscle contraction method, improving the trunk tension of children with low muscle tension and enhancing the muscle strength of the core muscle group, thus restoring the too weak muscle tension. Therefore, we speculate that intervention in different parts of children with different types of CP can have varying effects. KT can reduce the degree of spasm of high-tension muscles (25) in children with spasm. Shi mentioned that it can reshape the mechanical mode by relaxing muscle groups and fascia (25). Zhou speculated that it may provide the brain with more physical input through KT intervention, generating more signals to the muscle group and increasing the tension of abnormally low muscle groups (24).

#### 4.2.4 Step speed and step length

Heterogeneity decreased substantially after sensitivity analyses culling studies (26). By reading the full article, it was found that it might be due to the motor training of the neuroprotective therapy used in this study, which targeted the lower limbs of the children for improvement using the PNF and Bobath techniques, which might be the source of the heterogeneity.

#### 4.2.5 Step frequency

There was slight heterogeneity in this study. Heterogeneity disappeared after excluding the study (20) by sensitivity analysis. After subgroup analyses, it was found that the heterogeneity also came from the subgroup containing (20), and the remaining subgroups had a heterogeneity of 0. It was hypothesized that the heterogeneity originated from (20), which could be due to differences in the way step frequency was assessed.

### 4.3 Discussion of results of subgroup analysis

We found that KT based on exercise training is significant, which is consistent with the findings of other studies (21, 33, 34). It can be concluded that KT has a positive effect on gross exercise capabilities and balance. After analyzing the groups, the research effect of the 8-week intervention is even more apparent. The varying effects of different choices of taping sites remain an issue for further study. Following our analysis, the spinal taping method above the hip appears to be more relevant in terms of GMFM-D, GMFM-E, and balance. Interestingly, through the analysis of the duration of different intervention weeks, we found that the 8-week intervention research held significant significance, while the 10-week and 12-week interventions did not yield valid results. It may be that KT does not have a significant impact on long-term intervention effects and is recommended for multiple interventions within a medium-term period.

### 4.4 Analysis of mechanisms

Given the results of our meta-analysis, we hypothesize that the mechanisms of KT interventions on balance, gross motor function, and muscle tension may primarily consist of the following:

1. Proprioceptive mechanism

We hypothesize that KT may enhance balance and walking function by improving proprioceptive input and neuromuscular control. When applied to the skin, KT stimulates proprioceptive receptors, thereby increasing sensory feedback from the muscles, joints, and other body parts. This enhanced input allows for a more accurate representation of body position and movement status, enabling the brain to better perceive body posture and motion, which in turn facilitates improved regulation and control of balance and gross motor function. Yazici’s study found that KT could enhance walking posture and increase sensory stimulation transmission to the ankle and foot in hemiplegic patients following a stroke. Furthermore, the study indicated that the effects of KT extend beyond superficial tissues, as it can also induce heterogeneous deformation of muscle tissues throughout the entire limb, as evidenced by MRI analysis (35).

It has been demonstrated that KT can enhance proprioceptive input to the core region, thereby activating core control of the trunk and improving balance function (36). Because of central nervous system damage, the brain is often unable to provide the appropriate force and posture necessary for controlling limb movement (37). Consequently, when analyzing and processing information from sensory organs, uncoordinated movements and imbalances in muscle strength frequently occur. The application of KT exerts stretch and pressure on the skin, thereby strengthening sensory feedback in the area surrounding the tape. This enhancement of sensory input, combined with the repeated transmission of motor information, can help regulate skeletal muscle activity and nerve function, promoting improved balance and coordination in children with cerebral palsy (36).

1. Biomechanical mechanism

In terms of biomechanical mechanisms, we hypothesized that KT functions by regulating imbalanced muscle groups. Specifically, it reduces the tension in spastic muscles while enhancing the strength of flaccid muscle groups. This approach aims to correct the original faulty force structure of the lower limbs and further promote gait balance in children with cerebral palsy, as well as the sequencing of force-generating muscle groups during gross motor activities. Gait tests revealed that by applying relaxing patches to improperly used muscles and stimulating patches to weakened muscles, we could adjust the tension of the primary posture-controlling muscles and correct the force lines of the lower limbs. As a result, motor efficiency improved, abnormal gait patterns were alleviated, and children with cerebral palsy demonstrated enhancements in gross motor functions and balance abilities (27), which aligns with our hypothesis. El Shemy (38) and Bulent Elbasan (39), among others, have proposed that taping techniques can be used to activate certain muscles, induce muscle relaxation, and alleviate muscle spasm, thereby enhancing lower limb alignment and coordination. By adjusting the muscle tension in the lower limbs, improvements in motor function can be achieved.

### 4.5 Research strengths and limitations

This study found that KT is now only appropriate as a supplementary intervention to exercise treatment, not as a replacement for the original fundamental exercise training. KT improves some functions but does not fully affect others. Systematic reviews have shown that KT, as an adjuvant intervention strategy, may produce superior effects when combined with physiotherapy, such as exercise training. Research by Güçhan (40) suggests that taping is an increasingly popular adjunct to therapy because it is easy to apply, inexpensive, and can be easily removed or changed according to therapy objectives.

Although the RCTs included in this meta-analysis had detailed and specific inclusion criteria, the quality of the articles was somewhat limited due to the lack of blinding. The quality of the included literature was almost always ‘Fair’ grade, with some risk of bias. Given the poor quality of the included studies and the risk of bias, they still need to be interpreted with caution. It was challenging to group children with different types of CP and ages in a detailed and precise manner, making it difficult to determine the specific effects of KT on children of different age groups and CP types. Although the included randomized controlled trials did not mention adverse effects of KT, the use of KT may lead to skin reactions in some children with CP. The mechanisms by which KT works remain unclear. The effects of KT on muscle tension in children with various types of CP still need investigation, and more clinical trials are necessary to study the effects further.

## 5 Conclusion

This review highlights the effectiveness of KT in ameliorating specific symptoms in children with CP. The results show that KT has benefits on gross motor function and balance. However, the effect on walking is less clear, indicating areas for future research focus. The internal effect of KT as an adjunct intervention method cannot substitute sports training. There is a need to enhance the utilization of KT in rehabilitation therapy, despite potential increased treatment costs. The internal effects and impact of KT on children with CP, as well as the underlying mechanisms, warrant further exploration through additional clinical trials.

## Data Availability

The original contributions presented in the study are included in the article/Supplementary material, further inquiries can be directed to the corresponding author.

## Supplementary material

SUPPLEMENTARY 1 Forest Plot of the outcomes analysis.

SUPPLEMENTARY 2 Subgroup analysis weeks.

SUPPLEMENTARY 3 Subgroup analysis site of adhesion.

SUPPLEMENTARY 4 Egger’s test.

SUPPLEMENTARY 5 Sensitive analysis.

SUPPLEMENTARY 6 Search strategy.

SUPPLEMENTARY 7 ROB assessment.

SUPPLEMENTARY 8 Risk of bias.
