# Supplementary material for: The effect of Kinesio Taping on motor function in children with cerebral palsy: a systematic review and meta-analysis of randomized controlled trials
Source: Front Neurol. 2025 Mar 6;16:1527308. doi: 10.3389/fneur.2025.1527308 (PMC11927513; doi:10.3389/fneur.2025.1527308)
Supplement: SUPPLEMENTARY 1 — Forest Plot of the outcomes analysis. [file Data_Sheet_1.pdf]

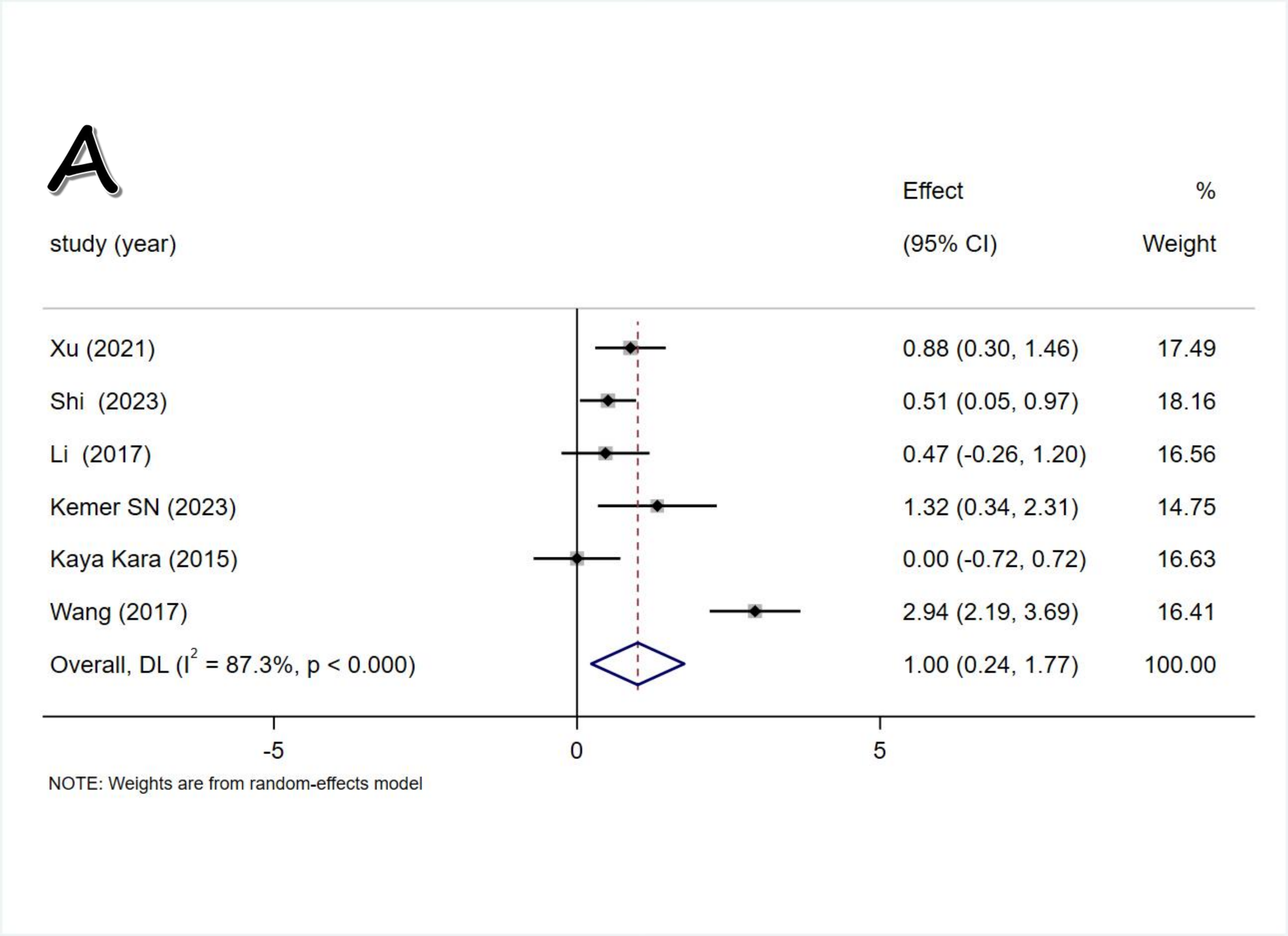
A
study (year)
Effect
(95% CI)
%
Weight
Xu (2021)
0.88 (0.30, 1.46)
17.49
Shi (2023)
0.51 (0.05, 0.97)
18.16
Li (2017)
0.47 (-0.26, 1.20)
16.56
Kemer SN (2023)
1.32 (0.34, 2.31)
14.75
Kaya Kara (2015)
0.00 (-0.72, 0.72)
16.63
Wang (2017)
2.94 (2.19, 3.69)
16.41
Overall, DL (I² = 87.3%, p < 0.000)
1.00 (0.24, 1.77)
100.00
-5
0
5
NOTE: Weights are from random-effects model

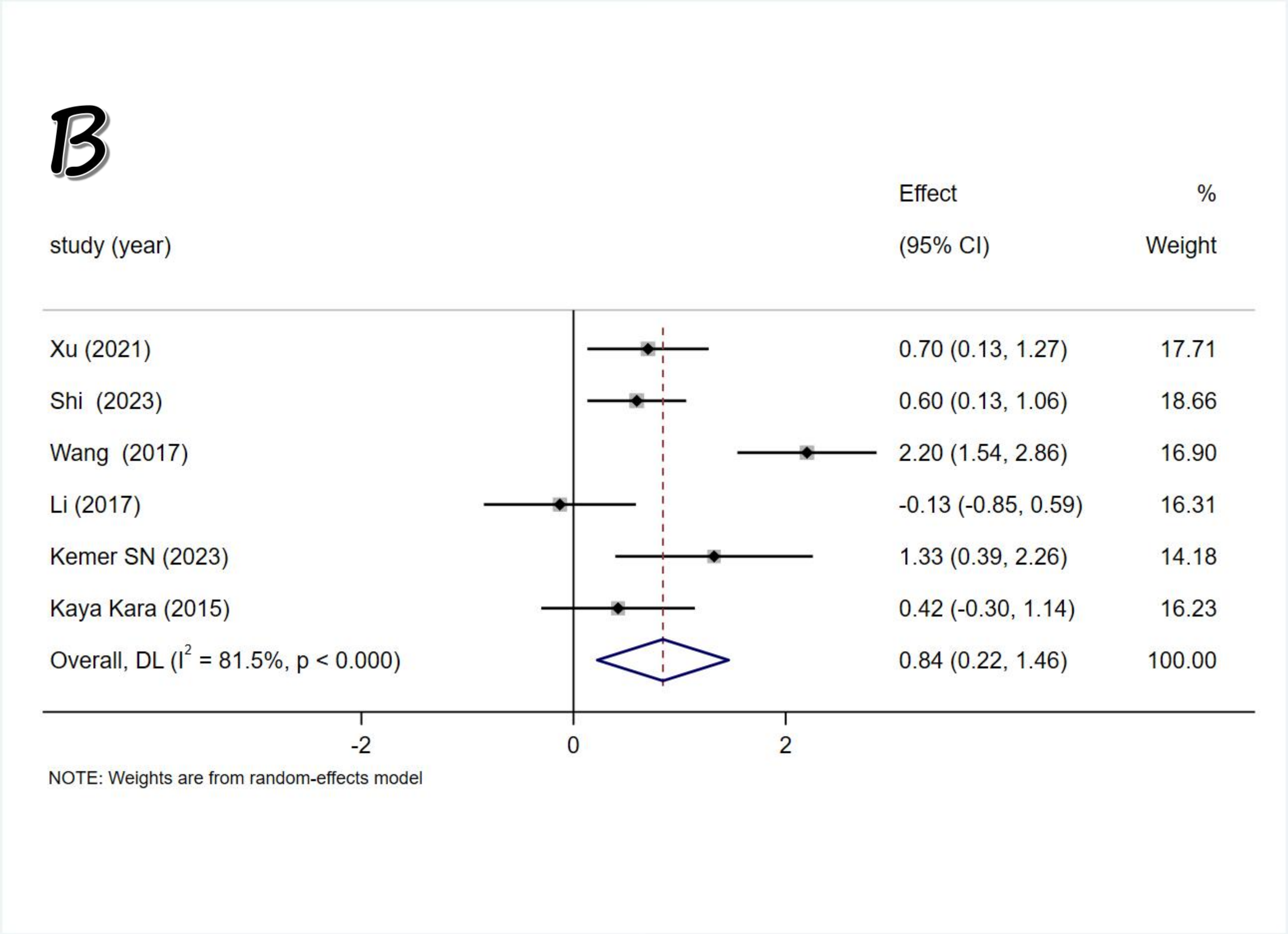
B
study (year)
Effect (95% CI)
% Weight
Xu (2021) 0.70 (0.13, 1.27) 17.71
Shi (2023) 0.60 (0.13, 1.06) 18.66
Wang (2017) 2.20 (1.54, 2.86) 16.90
Li (2017) -0.13 (-0.85, 0.59) 16.31
Kemer SN (2023) 1.33 (0.39, 2.26) 14.18
Kaya Kara (2015) 0.42 (-0.30, 1.14) 16.23
Overall, DL (I2 = 81.5%, p < 0.000) 0.84 (0.22, 1.46) 100.00
-2
0
2
NOTE: Weights are from random-effects model

C

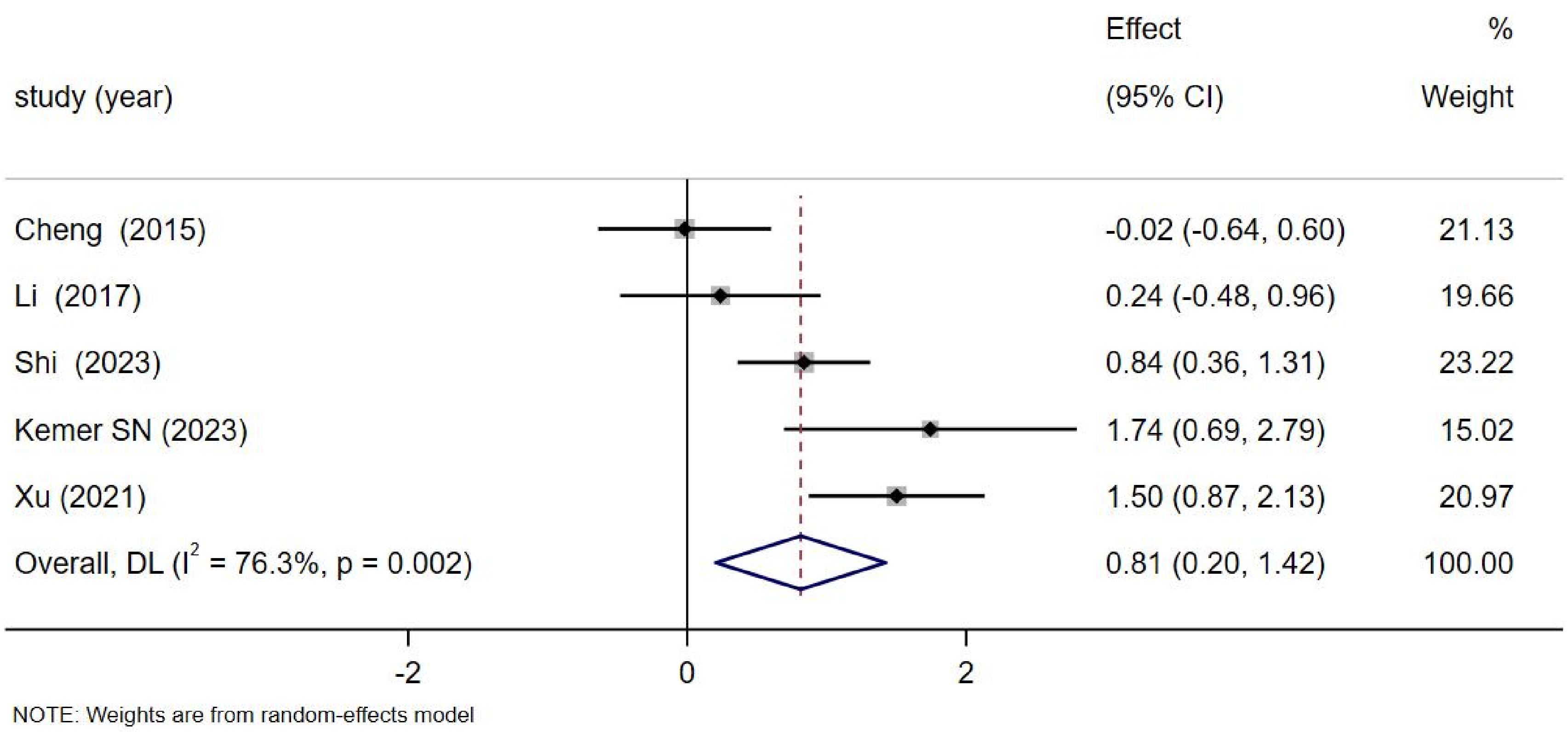
Effect
%
study (year)
(95% CI)
Weight
Cheng (2015)
-0.02 (-0.64, 0.60)
21.13
Li (2017)
0.24 (-0.48, 0.96)
19.66
Shi (2023)
0.84 (0.36, 1.31)
23.22
Kemer SN (2023)
1.74 (0.69, 2.79)
15.02
Xu (2021)
1.50 (0.87, 2.13)
20.97
Overall, DL ($I^2$ = 76.3%, p = 0.002)
0.81 (0.20, 1.42)
100.00
-2
0
2
NOTE: Weights are from random-effects model

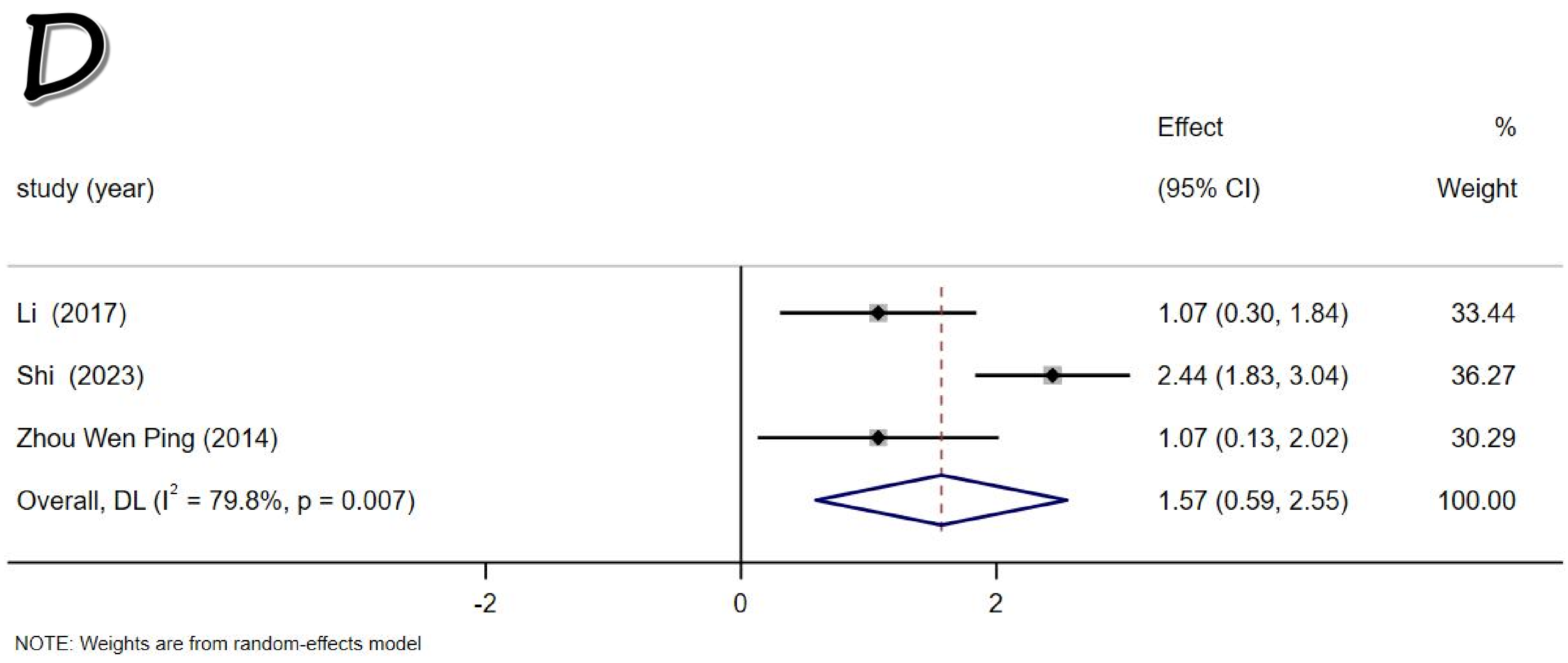
D
study (year)
Effect (95% CI)
% Weight
Li (2017)
1.07 (0.30, 1.84)
33.44
Shi (2023)
2.44 (1.83, 3.04)
36.27
Zhou Wen Ping (2014)
1.07 (0.13, 2.02)
30.29
Overall, DL (I2 = 79.8%, p = 0.007)
1.57 (0.59, 2.55)
100.00
-2
0
2
NOTE: Weights are from random-effects model

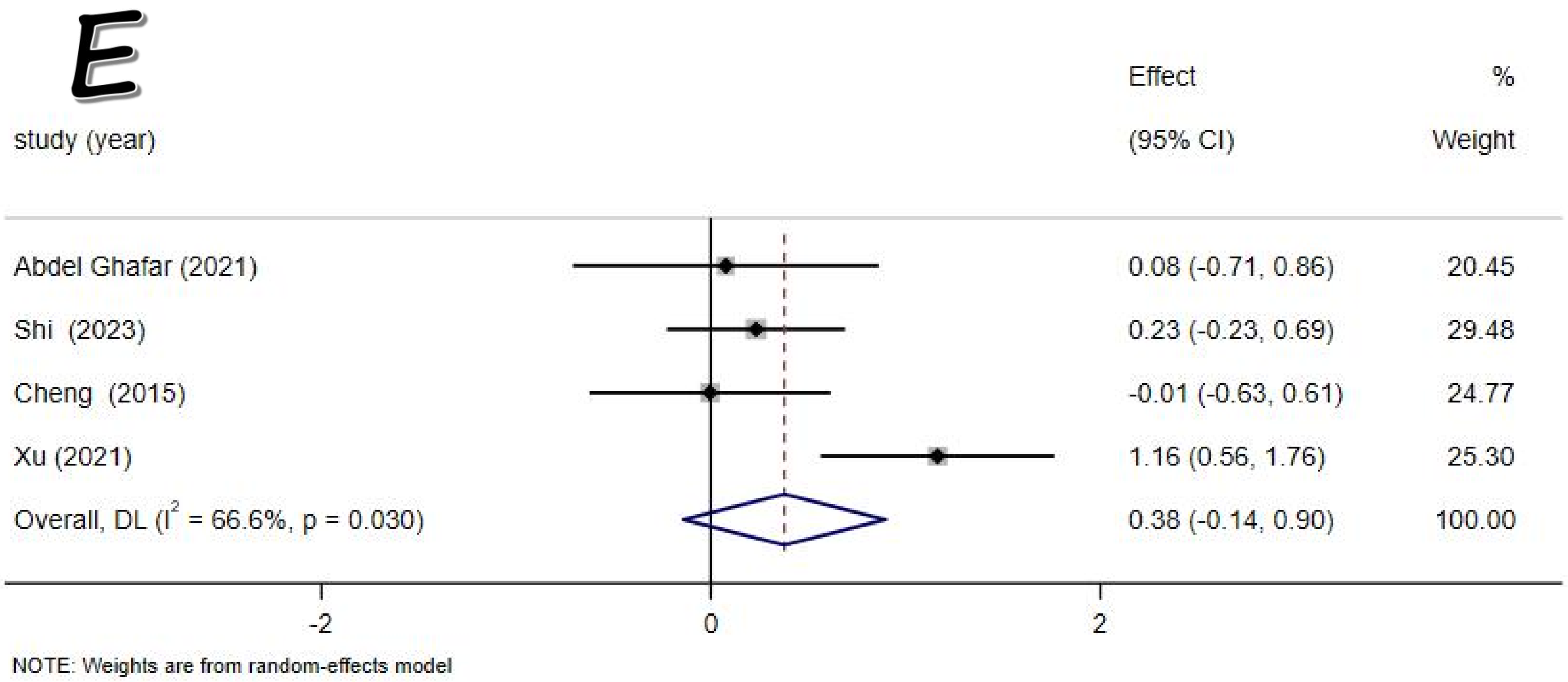
E
study (year)
Effect
(95% CI)
%
Weight
Abdel Ghafar (2021)
0.08 (-0.71, 0.86)
20.45
Shi (2023)
0.23 (-0.23, 0.69)
29.48
Cheng (2015)
-0.01 (-0.63, 0.61)
24.77
Xu (2021)
1.16 (0.56, 1.76)
25.30
Overall, DL (I2 = 66.6%, p = 0.030)
0.38 (-0.14, 0.90)
100.00
-2
0
2
NOTE: Weights are from random-effects model

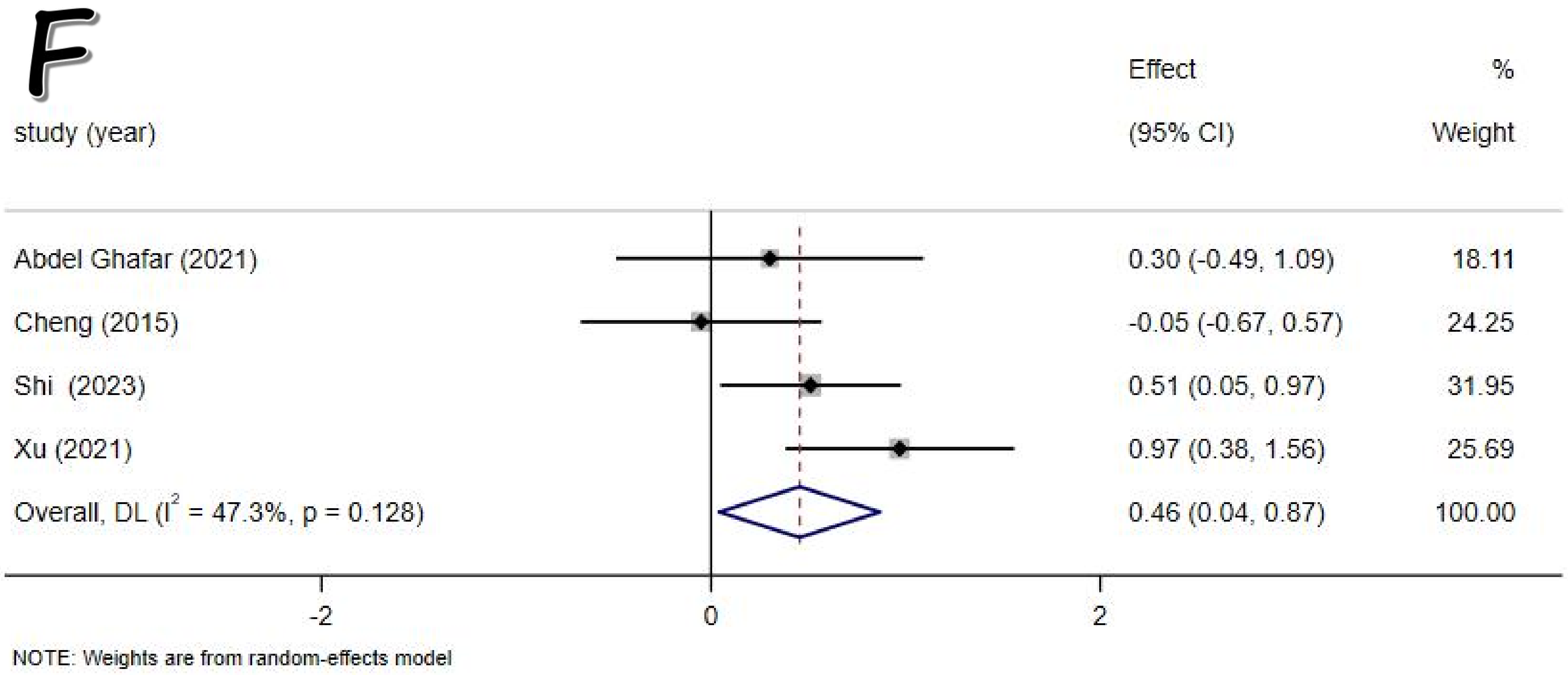
F
study (year)
Effect (95% CI)
% Weight
Abdel Ghafar (2021) 0.30 (-0.49, 1.09) 18.11
Cheng (2015) -0.05 (-0.67, 0.57) 24.25
Shi (2023) 0.51 (0.05, 0.97) 31.95
Xu (2021) 0.97 (0.38, 1.56) 25.69
Overall, DL ($I^2$ = 47.3%, p = 0.128) 0.46 (0.04, 0.87) 100.00
-2
0
2
NOTE: Weights are from random-effects model

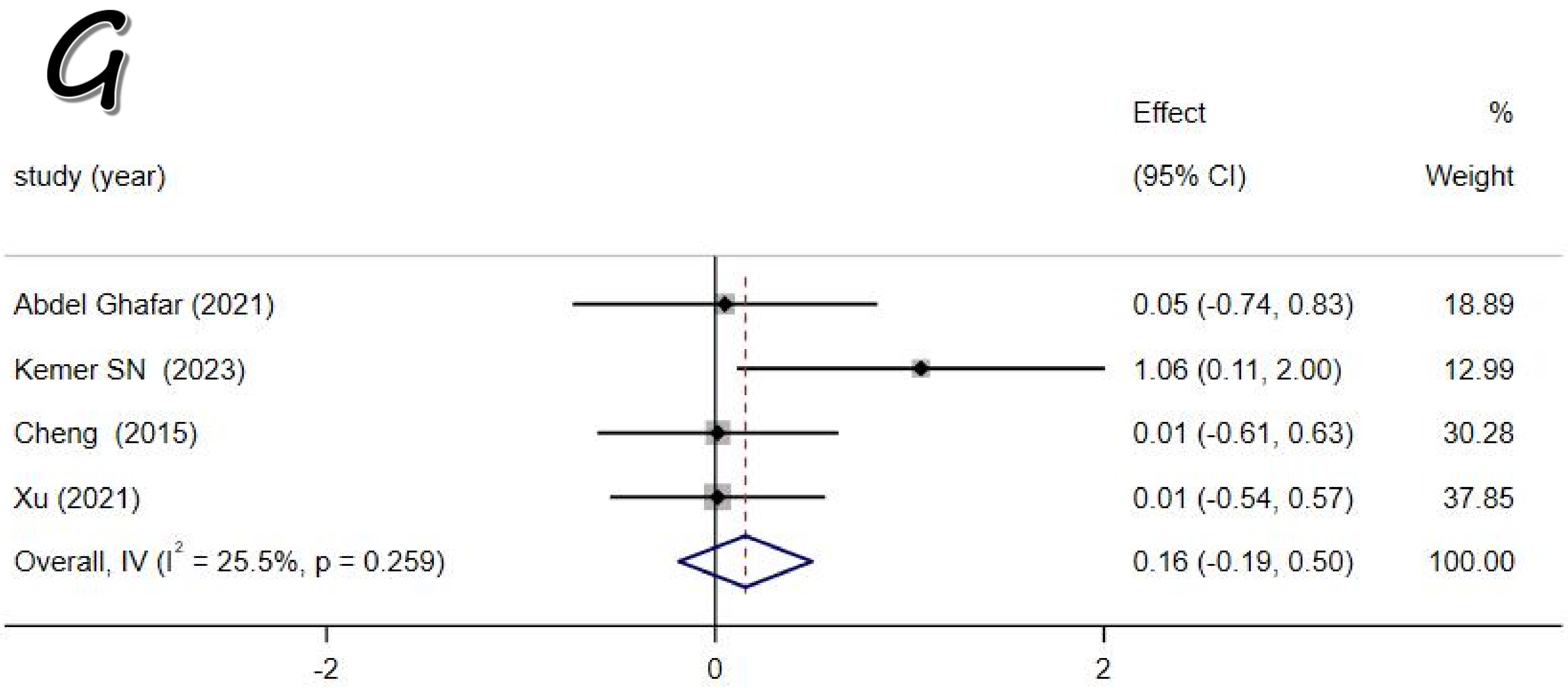
G
Effect
%
study (year)
(95% CI)
Weight
Abdel Ghafar (2021)
0.05 (-0.74, 0.83)
18.89
Kemer SN (2023)
1.06 (0.11, 2.00)
12.99
Cheng (2015)
0.01 (-0.61, 0.63)
30.28
Xu (2021)
0.01 (-0.54, 0.57)
37.85
Overall, IV (I² = 25.5%, p = 0.259)
0.16 (-0.19, 0.50)
100.00
-2
0
2

Forest Plot of the outcomes analysis：

A： GMFM-D B:GMFM-E C:BBS D:Muscle Tension-Heel-Ear Test

E: Step speed F: Step length G: Step frequency

Over DL (DerSimonian-Laird):

This is an indicator of heterogeneity calculated using the DerSimonian-Laird method.The DL method is a random effects model used to calculate the combined effect sizes and their confidence intervals.

Over IV (Inverse Variance):
This is an indicator of heterogeneity calculated using the inverse variance weighting method.The IV method is a fixed effects model.

$I^2$ (I-squared) is the percentage of heterogeneity and indicates the proportion of between-stud y variation to the total variation. p-values were used to test whether the heterogeneity was sig nificant or not, (usually $p < 0.05$ is considered significant).
