# Supplementary material for: The effect of Kinesio Taping on motor function in children with cerebral palsy: a systematic review and meta-analysis of randomized controlled trials
Source: Front Neurol. 2025 Mar 6;16:1527308. doi: 10.3389/fneur.2025.1527308 (PMC11927513; doi:10.3389/fneur.2025.1527308)
Supplement: SUPPLEMENTARY 2 — Subgroup analysis weeks. [file Data_Sheet_2.pdf]

1

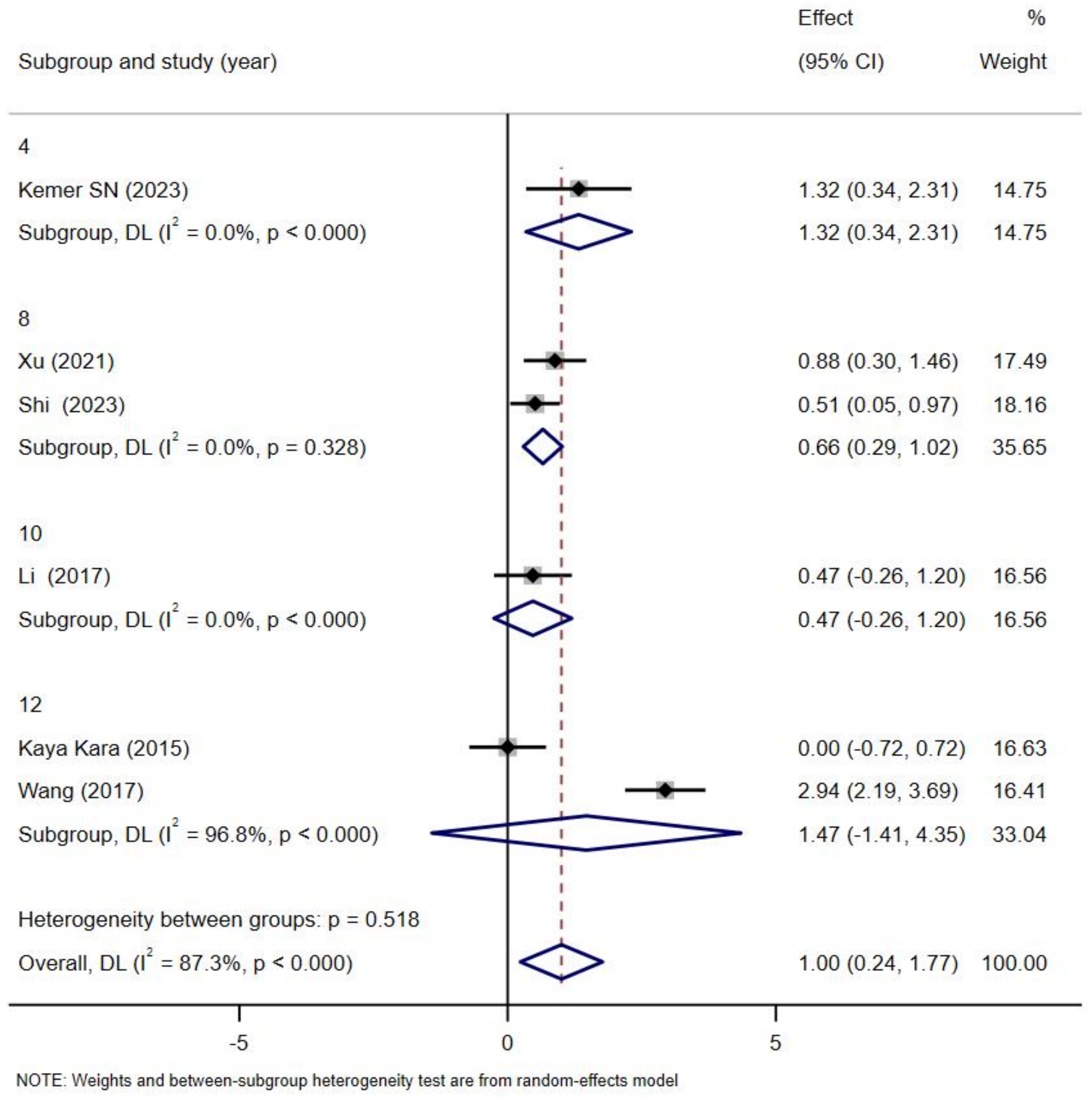
Effect
%
Subgroup and study (year)
(95% CI)
Weight
4
Kemer SN (2023)
1.32 (0.34, 2.31)
14.75
Subgroup, DL (I² = 0.0%, p < 0.000)
1.32 (0.34, 2.31)
14.75
8
Xu (2021)
0.88 (0.30, 1.46)
17.49
Shi (2023)
0.51 (0.05, 0.97)
18.16
Subgroup, DL (I² = 0.0%, p = 0.328)
0.66 (0.29, 1.02)
35.65
10
Li (2017)
0.47 (-0.26, 1.20)
16.56
Subgroup, DL (I² = 0.0%, p < 0.000)
0.47 (-0.26, 1.20)
16.56
12
Kaya Kara (2015)
0.00 (-0.72, 0.72)
16.63
Wang (2017)
2.94 (2.19, 3.69)
16.41
Subgroup, DL (I² = 96.8%, p < 0.000)
1.47 (-1.41, 4.35)
33.04
Heterogeneity between groups: p = 0.518
Overall, DL (I² = 87.3%, p < 0.000)
1.00 (0.24, 1.77)
100.00
-5
0
5
NOTE: Weights and between-subgroup heterogeneity test are from random-effects model

2

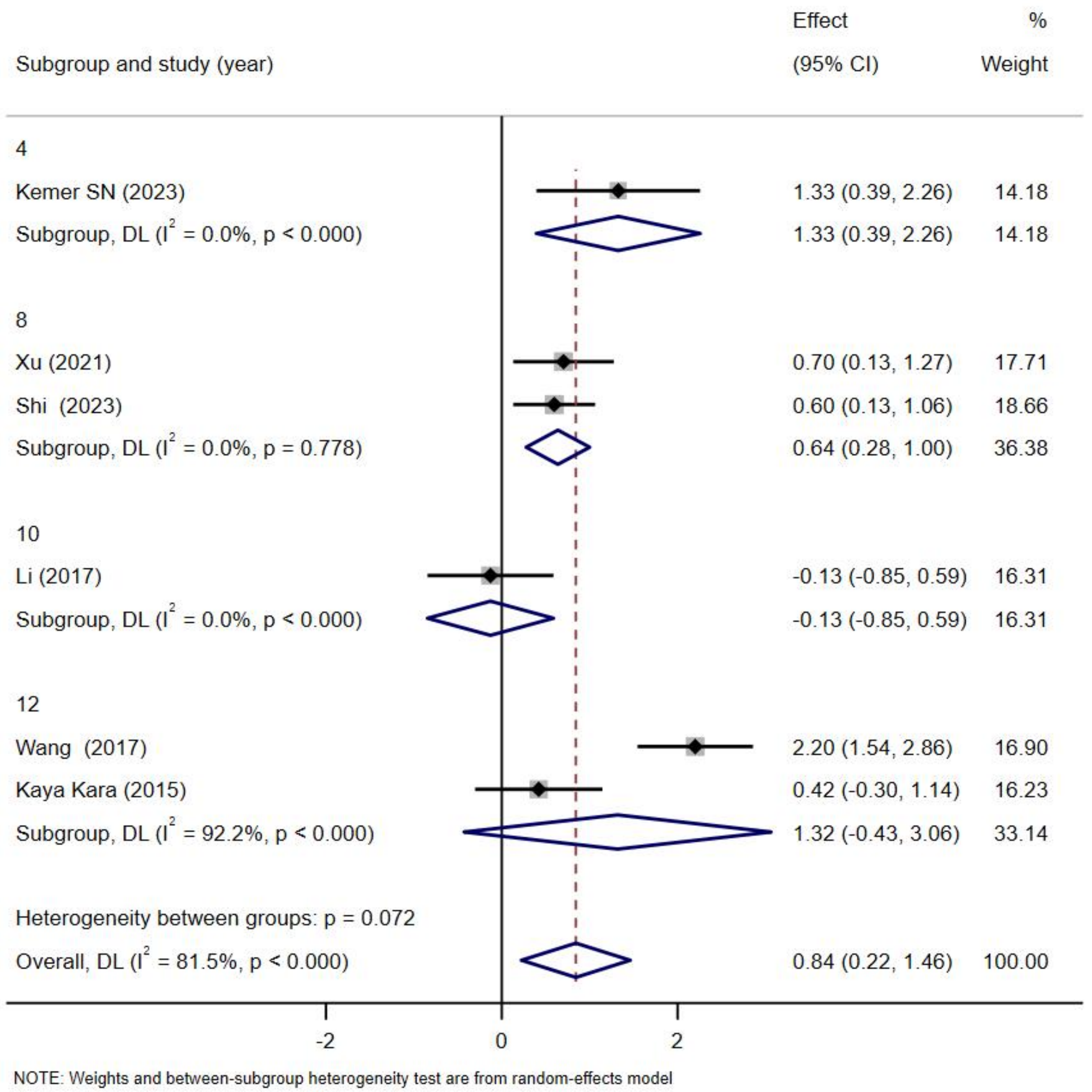

Subgroup and study (year)
Effect (95% CI)
% Weight
4
Kemer SN (2023) 1.33 (0.39, 2.26) 14.18
Subgroup, DL (I^2 = 0.0%, p < 0.000) 1.33 (0.39, 2.26) 14.18
8
Xu (2021) 0.70 (0.13, 1.27) 17.71
Shi (2023) 0.60 (0.13, 1.06) 18.66
Subgroup, DL (I^2 = 0.0%, p = 0.778) 0.64 (0.28, 1.00) 36.38
10
Li (2017) -0.13 (-0.85, 0.59) 16.31
Subgroup, DL (I^2 = 0.0%, p < 0.000) -0.13 (-0.85, 0.59) 16.31
12
Wang (2017) 2.20 (1.54, 2.86) 16.90
Kaya Kara (2015) 0.42 (-0.30, 1.14) 16.23
Subgroup, DL (I^2 = 92.2%, p < 0.000) 1.32 (-0.43, 3.06) 33.14
Heterogeneity between groups: p = 0.072
Overall, DL (I^2 = 81.5%, p < 0.000) 0.84 (0.22, 1.46) 100.00
-2
0
2
NOTE: Weights and between-subgroup heterogeneity test are from random-effects model

3

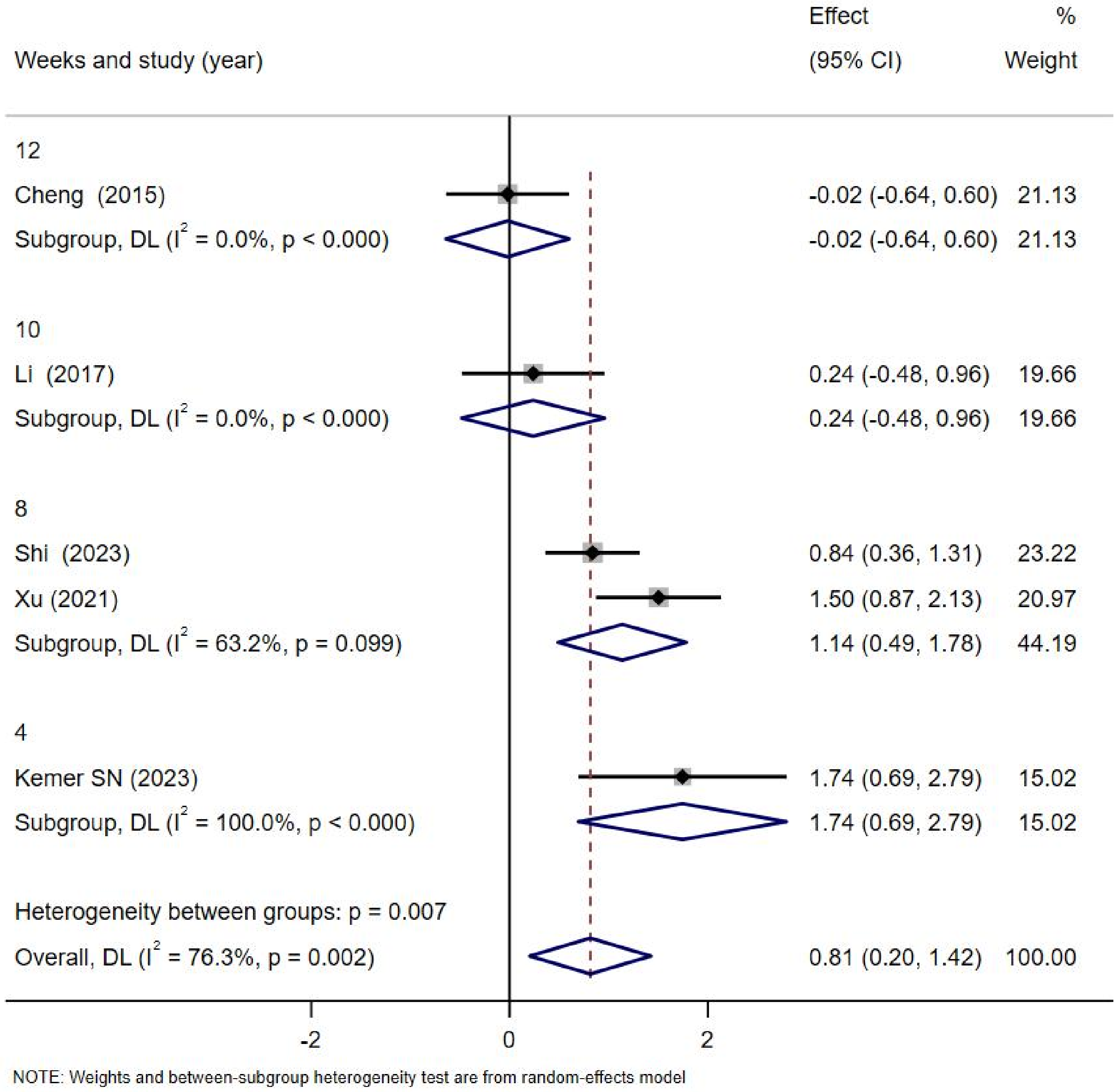

Weeks and study (year)
Effect (95% CI)
% Weight
12
Cheng (2015) -0.02 (-0.64, 0.60) 21.13
Subgroup, DL (I^2 = 0.0%, p < 0.000) -0.02 (-0.64, 0.60) 21.13
10
Li (2017) 0.24 (-0.48, 0.96) 19.66
Subgroup, DL (I^2 = 0.0%, p < 0.000) 0.24 (-0.48, 0.96) 19.66
8
Shi (2023) 0.84 (0.36, 1.31) 23.22
Xu (2021) 1.50 (0.87, 2.13) 20.97
Subgroup, DL (I^2 = 63.2%, p = 0.099) 1.14 (0.49, 1.78) 44.19
4
Kemer SN (2023) 1.74 (0.69, 2.79) 15.02
Subgroup, DL (I^2 = 100.0%, p < 0.000) 1.74 (0.69, 2.79) 15.02
Heterogeneity between groups: p = 0.007
Overall, DL (I^2 = 76.3%, p = 0.002) 0.81 (0.20, 1.42) 100.00
-2
0
2
NOTE: Weights and between-subgroup heterogeneity test are from random-effects model

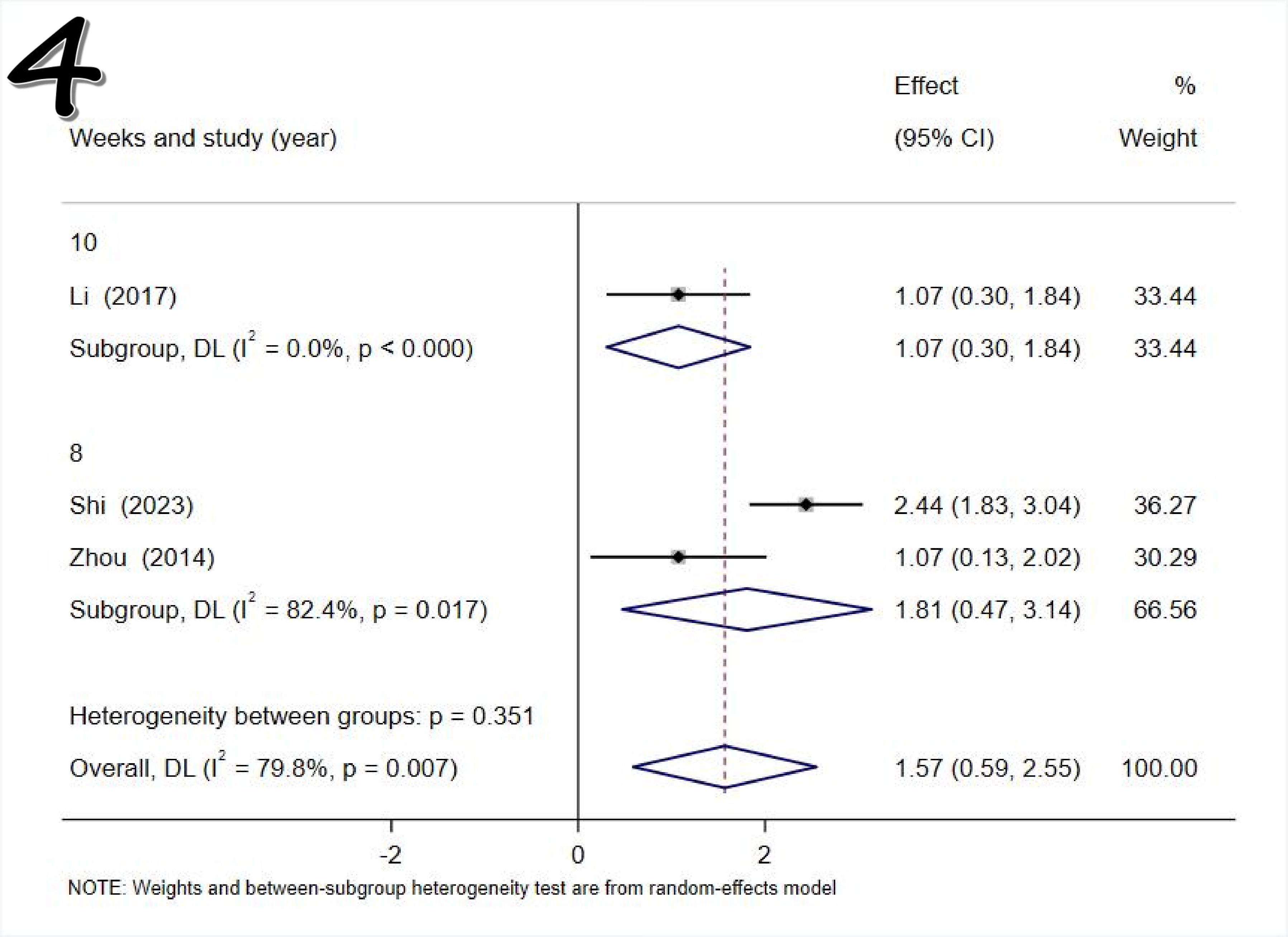
4
Weeks and study (year)
Effect (95% CI)
% Weight
10
Li (2017) 1.07 (0.30, 1.84) 33.44
Subgroup, DL (I^2 = 0.0%, p < 0.000) 1.07 (0.30, 1.84) 33.44
8
Shi (2023) 2.44 (1.83, 3.04) 36.27
Zhou (2014) 1.07 (0.13, 2.02) 30.29
Subgroup, DL (I^2 = 82.4%, p = 0.017) 1.81 (0.47, 3.14) 66.56
Heterogeneity between groups: p = 0.351
Overall, DL (I^2 = 79.8%, p = 0.007) 1.57 (0.59, 2.55) 100.00
-2
0
2
NOTE: Weights and between-subgroup heterogeneity test are from random-effects model

5

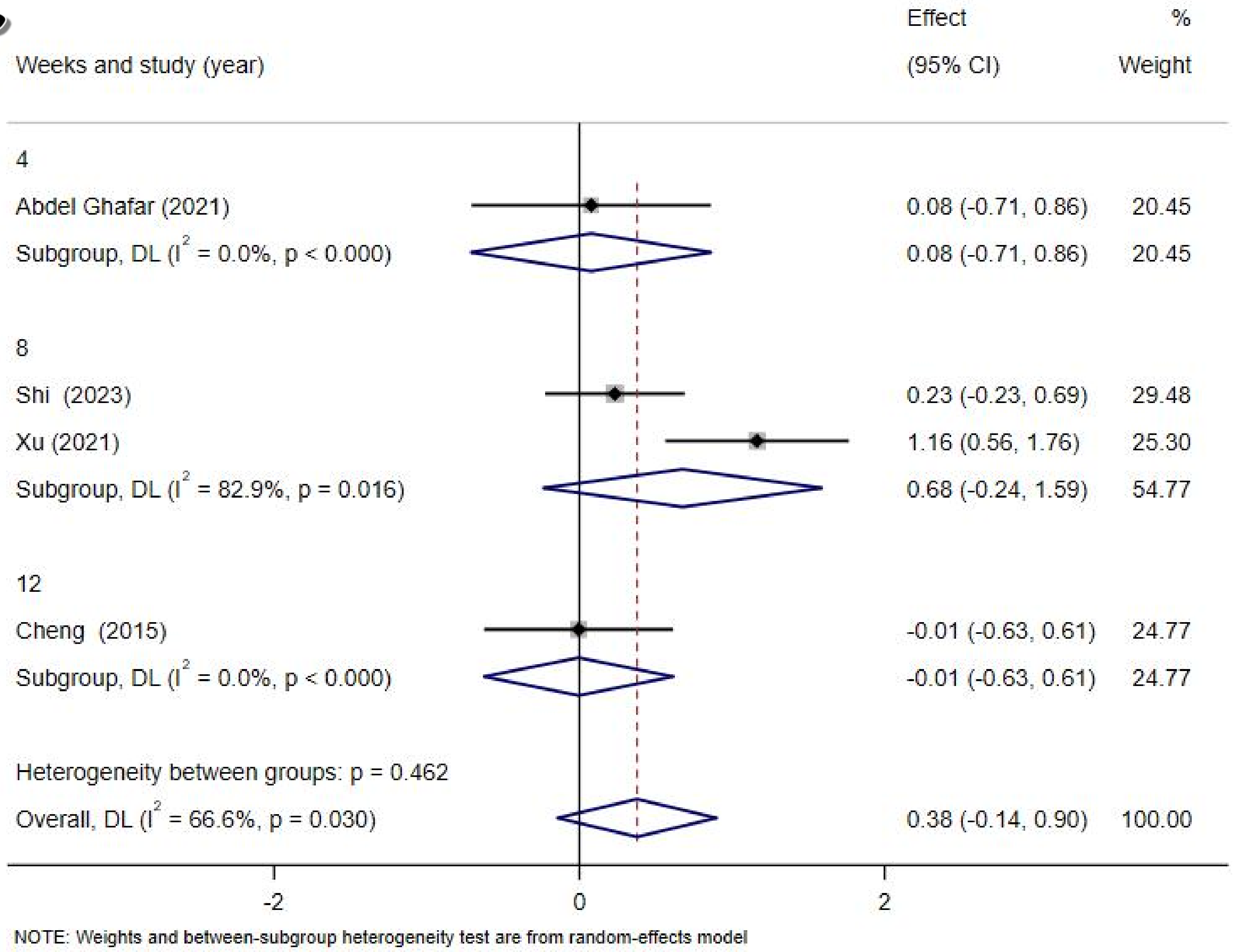
Weeks and study (year)
Effect (95% CI)
% Weight
4
Abdel Ghafar (2021) 0.08 (-0.71, 0.86) 20.45
Subgroup, DL (I2 = 0.0%, p < 0.000) 0.08 (-0.71, 0.86) 20.45
8
Shi (2023) 0.23 (-0.23, 0.69) 29.48
Xu (2021) 1.16 (0.56, 1.76) 25.30
Subgroup, DL (I2 = 82.9%, p = 0.016) 0.68 (-0.24, 1.59) 54.77
12
Cheng (2015) -0.01 (-0.63, 0.61) 24.77
Subgroup, DL (I2 = 0.0%, p < 0.000) -0.01 (-0.63, 0.61) 24.77
Heterogeneity between groups: p = 0.462
Overall, DL (I2 = 66.6%, p = 0.030) 0.38 (-0.14, 0.90) 100.00
-2
0
2
NOTE: Weights and between-subgroup heterogeneity test are from random-effects model

6

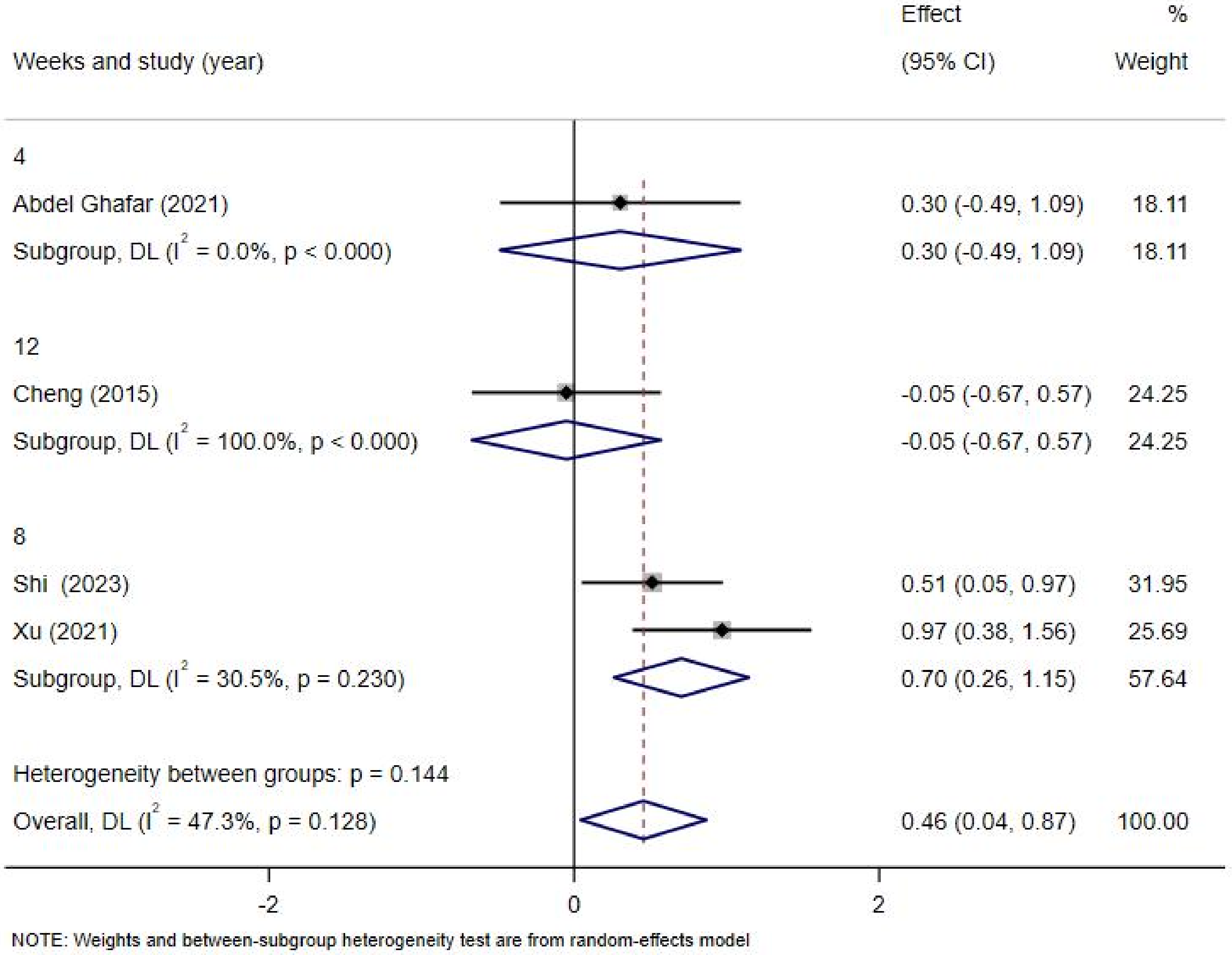

Weeks and study (year)
Effect (95% CI)
% Weight
4
Abdel Ghafar (2021) 0.30 (-0.49, 1.09) 18.11
Subgroup, DL (I^2 = 0.0%, p < 0.000) 0.30 (-0.49, 1.09) 18.11
12
Cheng (2015) -0.05 (-0.67, 0.57) 24.25
Subgroup, DL (I^2 = 100.0%, p < 0.000) -0.05 (-0.67, 0.57) 24.25
8
Shi (2023) 0.51 (0.05, 0.97) 31.95
Xu (2021) 0.97 (0.38, 1.56) 25.69
Subgroup, DL (I^2 = 30.5%, p = 0.230) 0.70 (0.26, 1.15) 57.64
Heterogeneity between groups: p = 0.144
Overall, DL (I^2 = 47.3%, p = 0.128) 0.46 (0.04, 0.87) 100.00
-2
0
2
NOTE: Weights and between-subgroup heterogeneity test are from random-effects model

7

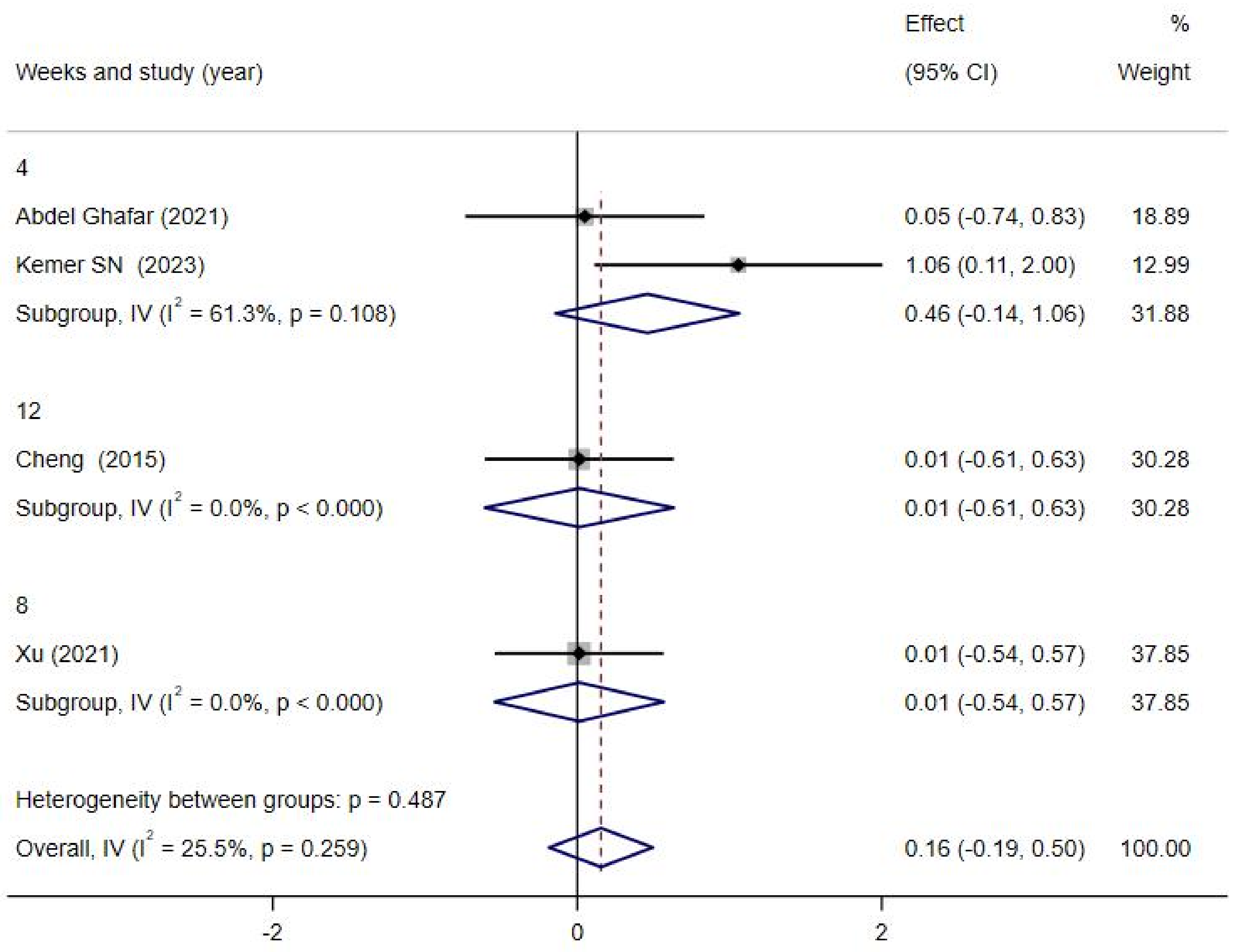

Effect
%
Weeks and study (year)
(95% CI)
Weight
4
Abdel Ghafar (2021)
0.05 (-0.74, 0.83)
18.89
Kemer SN (2023)
1.06 (0.11, 2.00)
12.99
Subgroup, IV (I² = 61.3%, p = 0.108)
0.46 (-0.14, 1.06)
31.88
12
Cheng (2015)
0.01 (-0.61, 0.63)
30.28
Subgroup, IV (I² = 0.0%, p < 0.000)
0.01 (-0.61, 0.63)
30.28
8
Xu (2021)
0.01 (-0.54, 0.57)
37.85
Subgroup, IV (I² = 0.0%, p < 0.000)
0.01 (-0.54, 0.57)
37.85
Heterogeneity between groups: p = 0.487
Overall, IV (I² = 25.5%, p = 0.259)
0.16 (-0.19, 0.50)
100.00
-2
0
2

Subgroup analysis weeks

1：GMFM-D　2:GMFM-E　3:BBS　4:Muscle Tension-Heel-Ear Test

5: Step speed　6: Step length　7: Step frequency

Over DL (DerSimonian-Laird):

This is an indicator of heterogeneity calculated using the DerSimonian-Laird method.The DL method is a random effects model used to calculate the combined effect sizes and their confidence intervals.

Over IV (Inverse Variance):

This is an indicator of heterogeneity calculated using the inverse variance weighting method.The IV method is a fixed effects model.

I^2 (I-squared) is the percentage of heterogeneity and indicates the proportion of between-study variation to the total variation. p-values were used to test whether the heterogeneity was significant or not, (usually $p < 0.05$ is considered significant).
