# Supplementary material for: The effect of Kinesio Taping on motor function in children with cerebral palsy: a systematic review and meta-analysis of randomized controlled trials
Source: Front Neurol. 2025 Mar 6;16:1527308. doi: 10.3389/fneur.2025.1527308 (PMC11927513; doi:10.3389/fneur.2025.1527308)
Supplement: SUPPLEMENTARY 3 — Subgroup analysis site of adhesion. [file Data_Sheet_3.pdf]

1

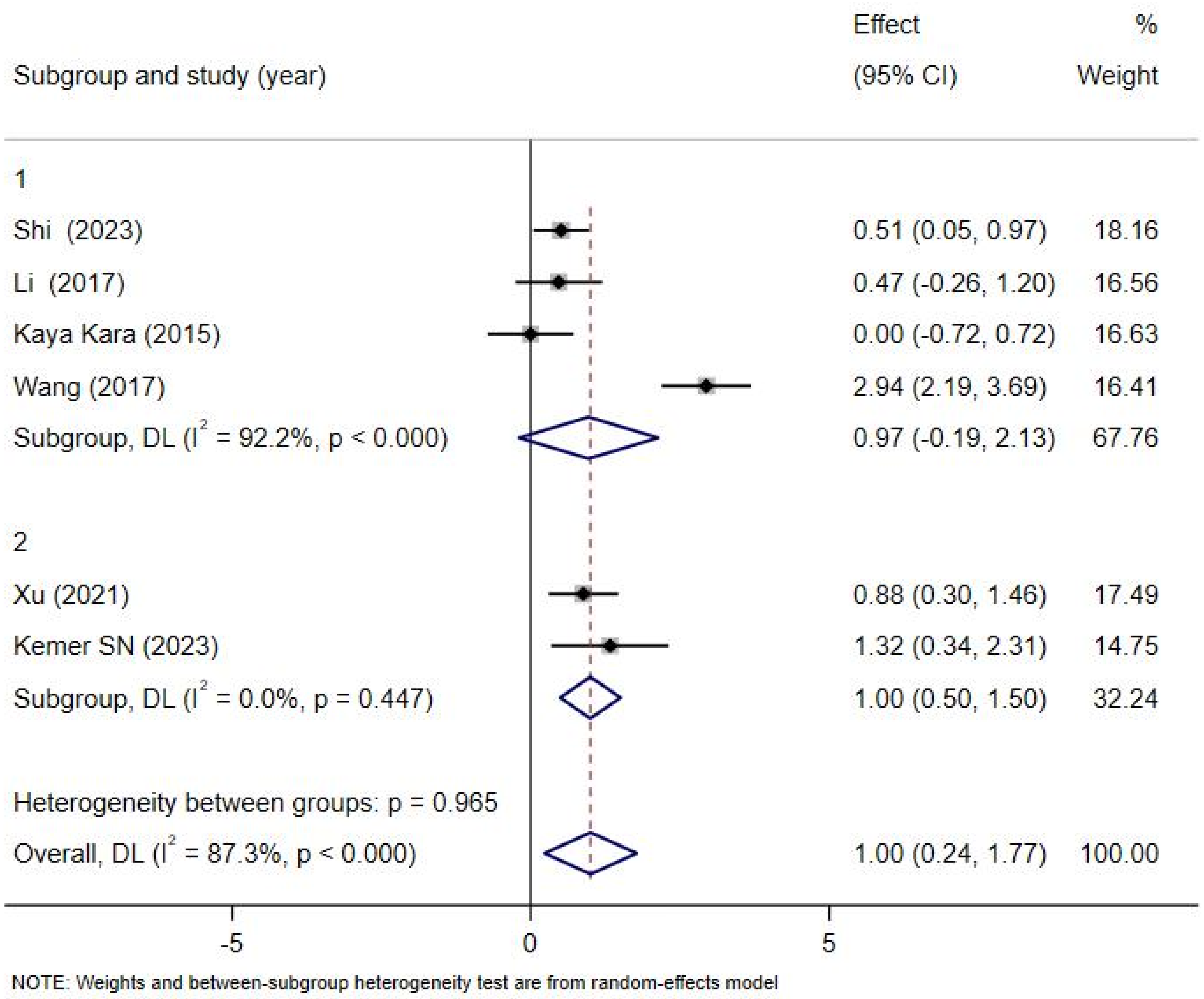
Subgroup and study (year)
Effect
(95% CI)
%
Weight
1
Shi (2023) 0.51 (0.05, 0.97) 18.16
Li (2017) 0.47 (-0.26, 1.20) 16.56
Kaya Kara (2015) 0.00 (-0.72, 0.72) 16.63
Wang (2017) 2.94 (2.19, 3.69) 16.41
Subgroup, DL (I² = 92.2%, p < 0.000) 0.97 (-0.19, 2.13) 67.76
2
Xu (2021) 0.88 (0.30, 1.46) 17.49
Kemer SN (2023) 1.32 (0.34, 2.31) 14.75
Subgroup, DL (I² = 0.0%, p = 0.447) 1.00 (0.50, 1.50) 32.24
Heterogeneity between groups: p = 0.965
Overall, DL (I² = 87.3%, p < 0.000) 1.00 (0.24, 1.77) 100.00
-5
0
5
NOTE: Weights and between-subgroup heterogeneity test are from random-effects model

2

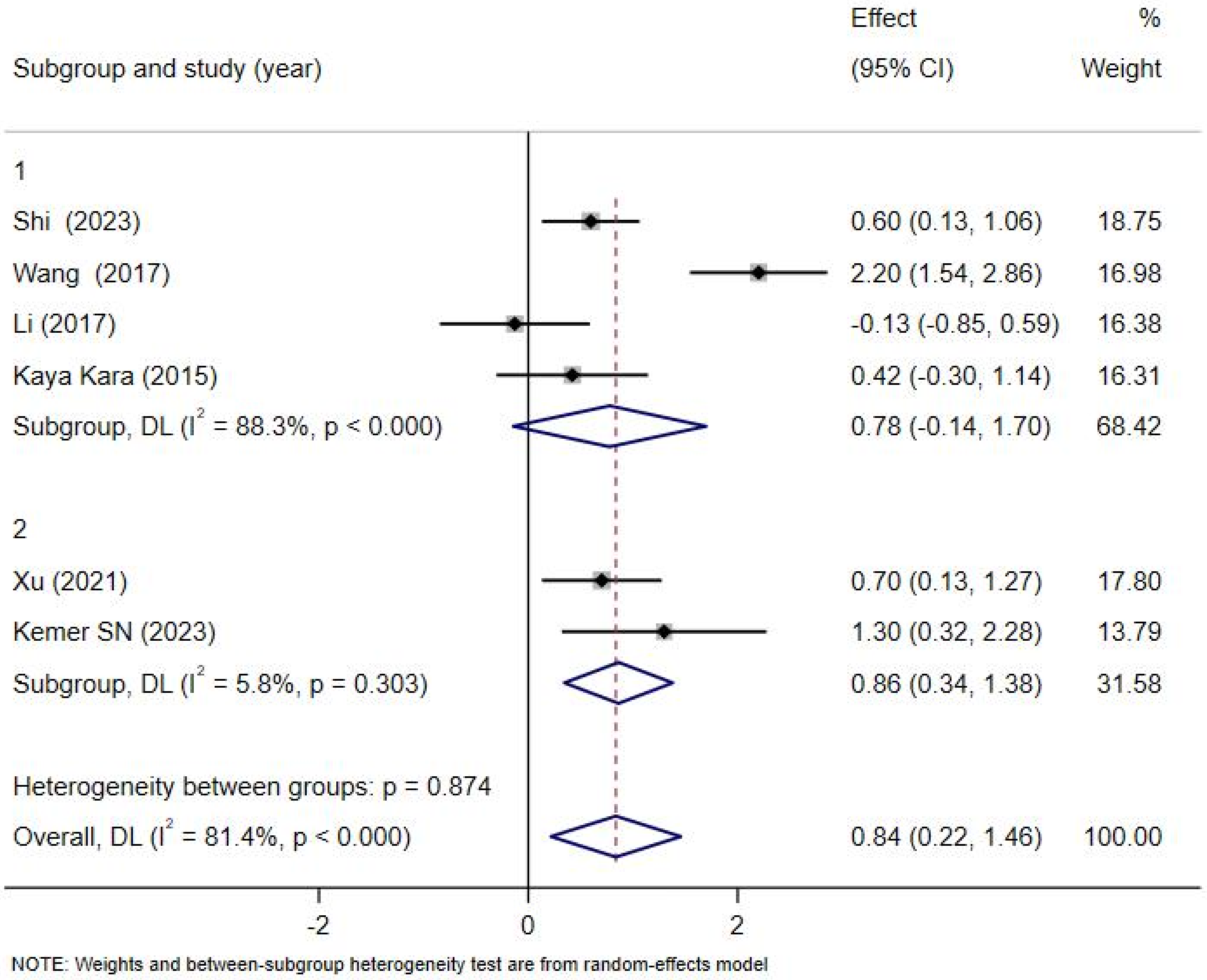
Effect
%
Subgroup and study (year)
(95% CI)
Weight
1
Shi (2023)
0.60 (0.13, 1.06)
18.75
Wang (2017)
2.20 (1.54, 2.86)
16.98
Li (2017)
-0.13 (-0.85, 0.59)
16.38
Kaya Kara (2015)
0.42 (-0.30, 1.14)
16.31
Subgroup, DL (I^2 = 88.3%, p < 0.000)
0.78 (-0.14, 1.70)
68.42
2
Xu (2021)
0.70 (0.13, 1.27)
17.80
Kemer SN (2023)
1.30 (0.32, 2.28)
13.79
Subgroup, DL (I^2 = 5.8%, p = 0.303)
0.86 (0.34, 1.38)
31.58
Heterogeneity between groups: p = 0.874
Overall, DL (I^2 = 81.4%, p < 0.000)
0.84 (0.22, 1.46)
100.00
-2
0
2
NOTE: Weights and between-subgroup heterogeneity test are from random-effects model

3

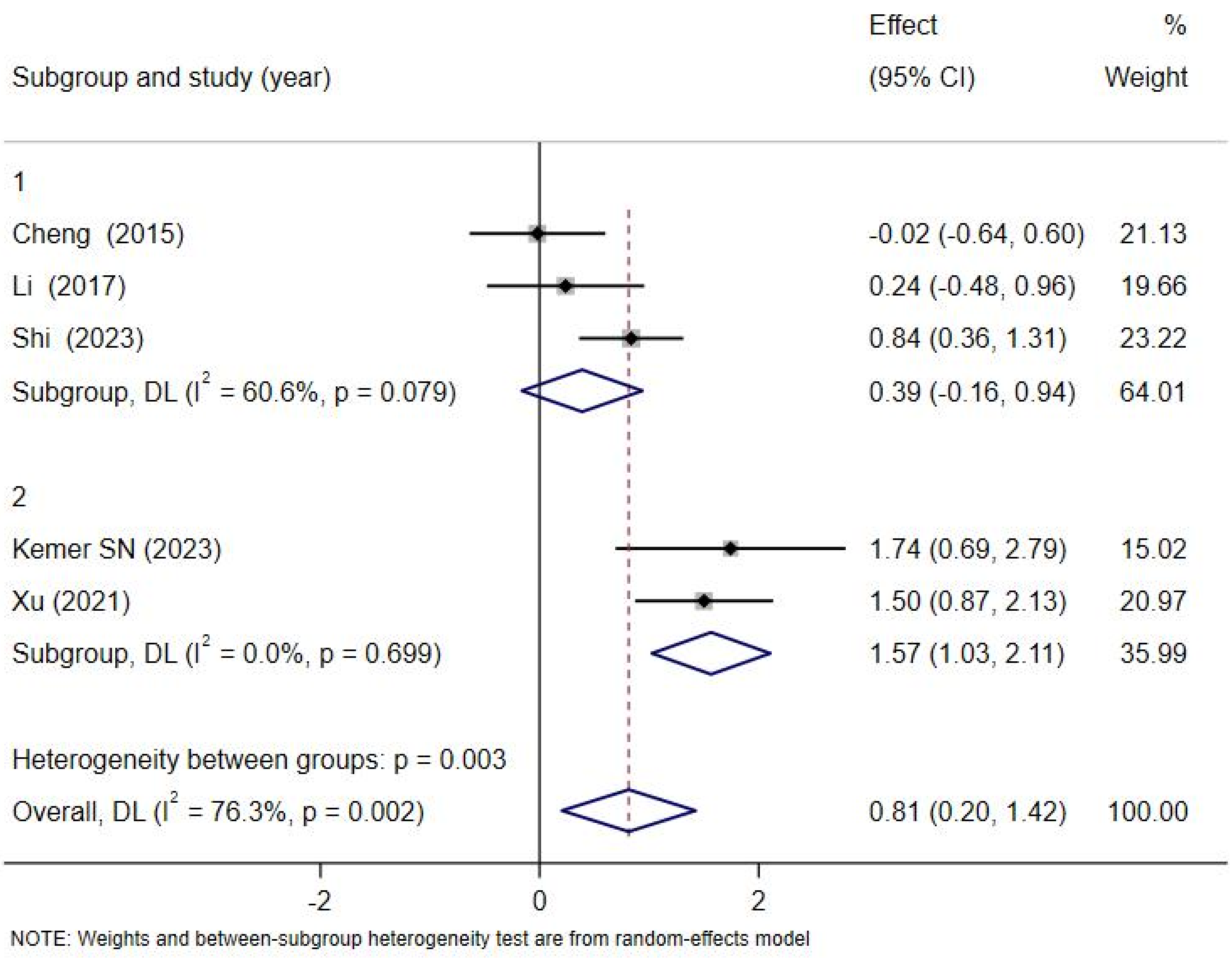

Effect
%
Subgroup and study (year)
(95% CI)
Weight
1
Cheng (2015)
-0.02 (-0.64, 0.60)
21.13
Li (2017)
0.24 (-0.48, 0.96)
19.66
Shi (2023)
0.84 (0.36, 1.31)
23.22
Subgroup, DL (I^2 = 60.6%, p = 0.079)
0.39 (-0.16, 0.94)
64.01
2
Kemer SN (2023)
1.74 (0.69, 2.79)
15.02
Xu (2021)
1.50 (0.87, 2.13)
20.97
Subgroup, DL (I^2 = 0.0%, p = 0.699)
1.57 (1.03, 2.11)
35.99
Heterogeneity between groups: p = 0.003
Overall, DL (I^2 = 76.3%, p = 0.002)
0.81 (0.20, 1.42)
100.00
-2
0
2
NOTE: Weights and between-subgroup heterogeneity test are from random-effects model

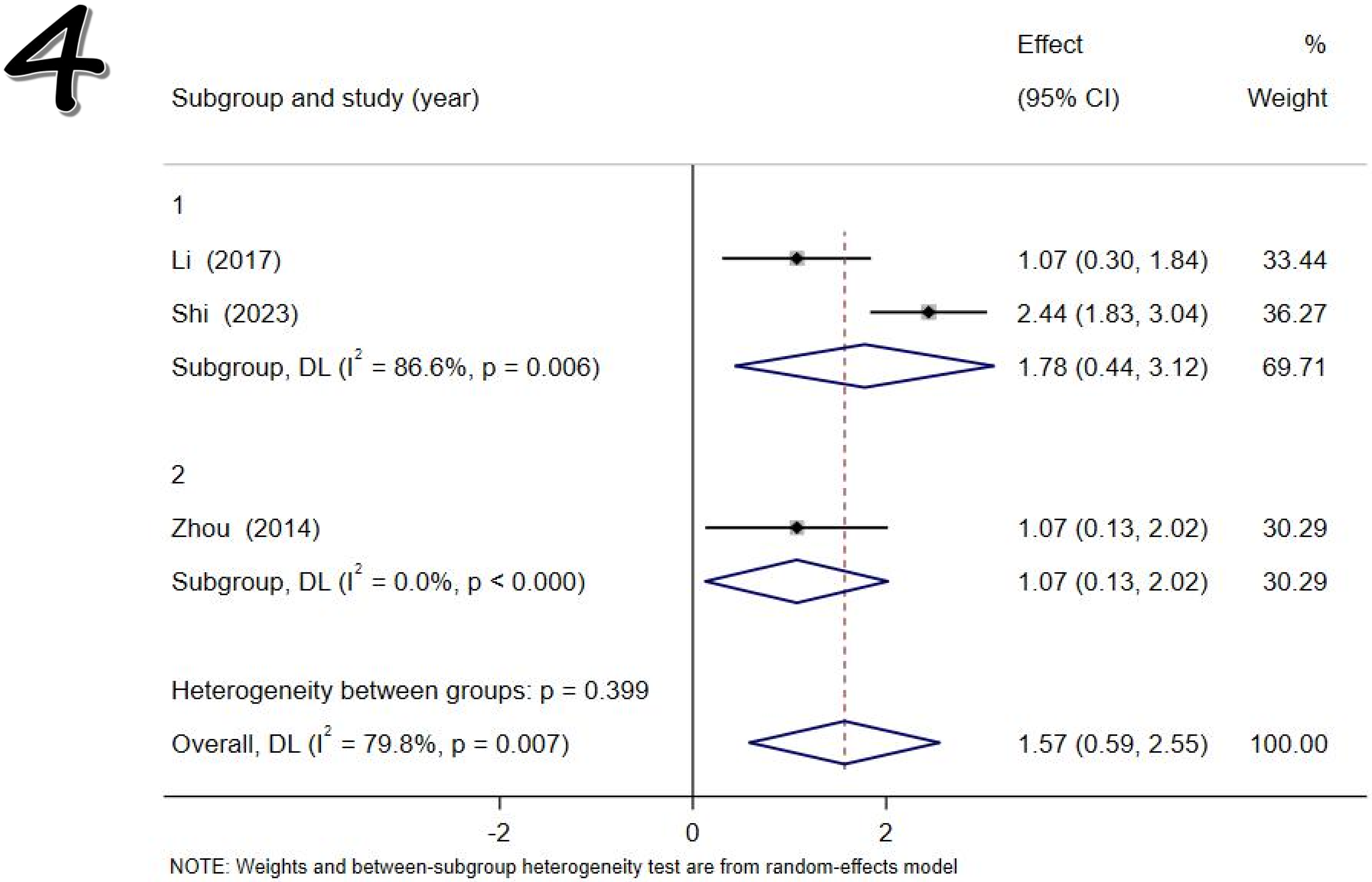
4
Subgroup and study (year)
Effect
(95% CI)
%
Weight
1
Li (2017)
1.07 (0.30, 1.84)
33.44
Shi (2023)
2.44 (1.83, 3.04)
36.27
Subgroup, DL (I^2 = 86.6%, p = 0.006)
1.78 (0.44, 3.12)
69.71
2
Zhou (2014)
1.07 (0.13, 2.02)
30.29
Subgroup, DL (I^2 = 0.0%, p < 0.000)
1.07 (0.13, 2.02)
30.29
Heterogeneity between groups: p = 0.399
Overall, DL (I^2 = 79.8%, p = 0.007)
1.57 (0.59, 2.55)
100.00
-2
0
2
NOTE: Weights and between-subgroup heterogeneity test are from random-effects model

5

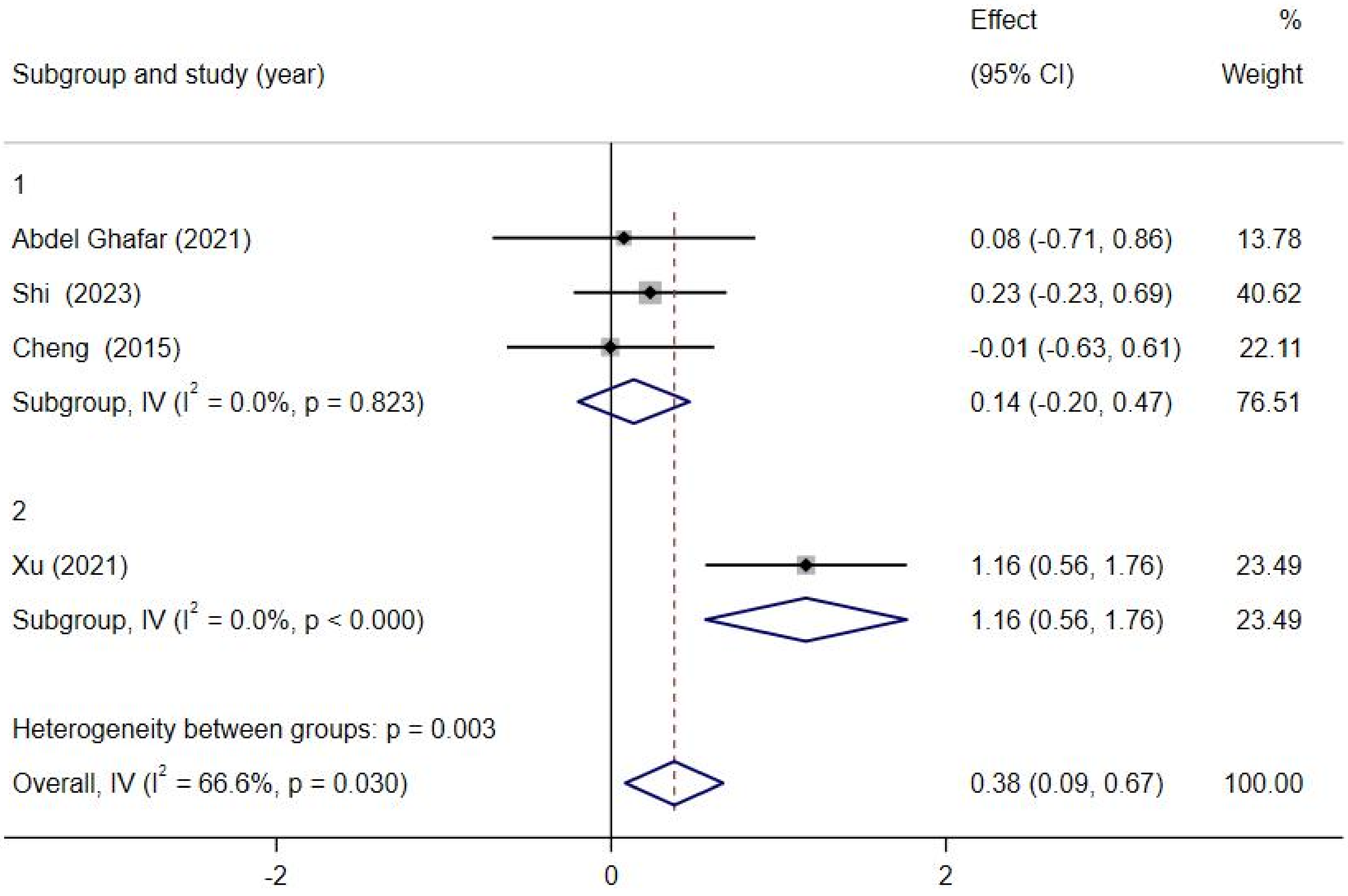
Effect
(95% CI)
%
Weight
Subgroup and study (year)
1
Abdel Ghafar (2021)
0.08 (-0.71, 0.86)
13.78
Shi (2023)
0.23 (-0.23, 0.69)
40.62
Cheng (2015)
-0.01 (-0.63, 0.61)
22.11
Subgroup, IV (I^2 = 0.0%, p = 0.823)
0.14 (-0.20, 0.47)
76.51
2
Xu (2021)
1.16 (0.56, 1.76)
23.49
Subgroup, IV (I^2 = 0.0%, p < 0.000)
1.16 (0.56, 1.76)
23.49
Heterogeneity between groups: p = 0.003
Overall, IV (I^2 = 66.6%, p = 0.030)
0.38 (0.09, 0.67)
100.00
-2
0
2

6

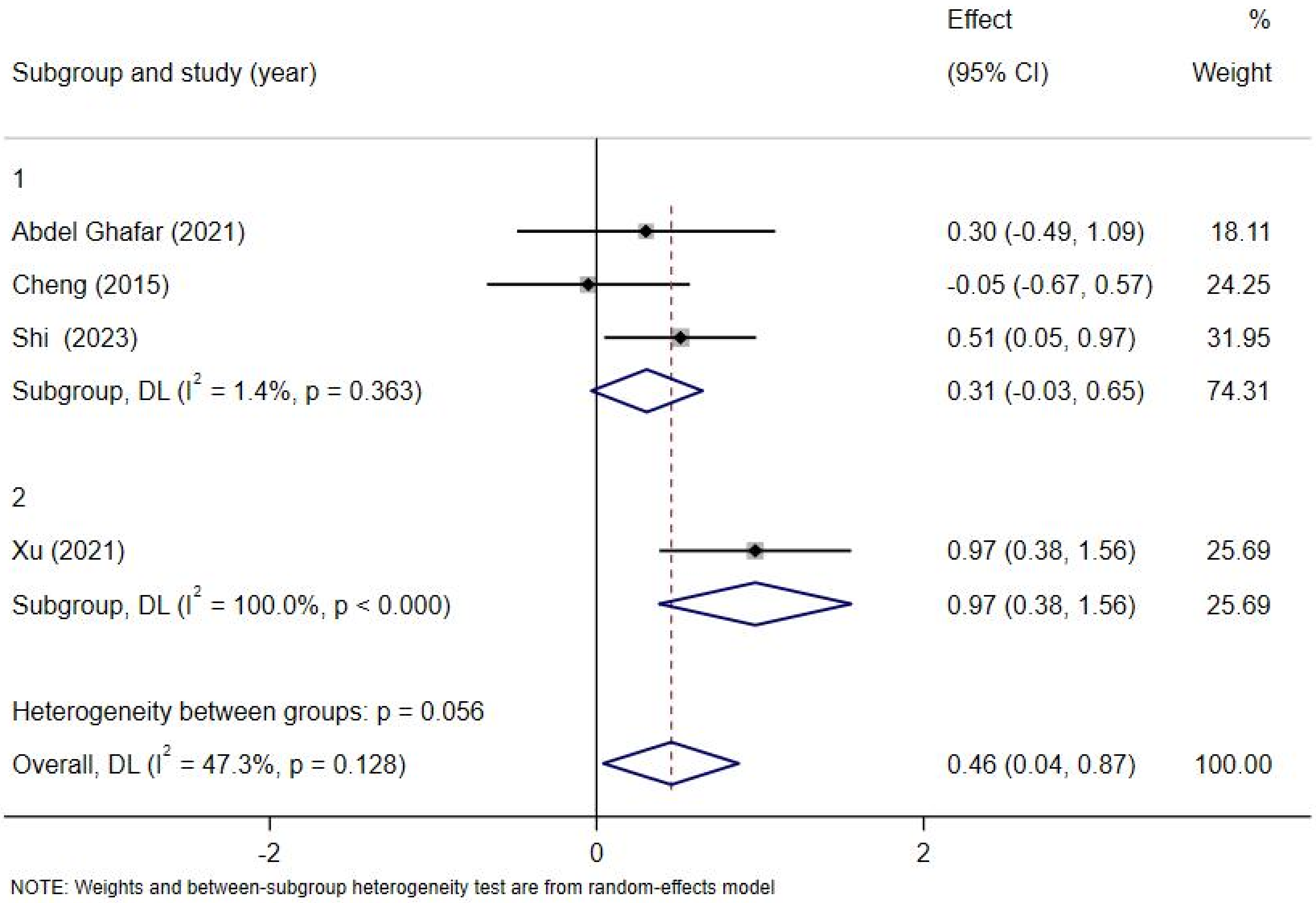
Subgroup and study (year)
Effect (95% CI)
% Weight
1
Abdel Ghafar (2021) 0.30 (-0.49, 1.09) 18.11
Cheng (2015) -0.05 (-0.67, 0.57) 24.25
Shi (2023) 0.51 (0.05, 0.97) 31.95
Subgroup, DL (I^2 = 1.4%, p = 0.363) 0.31 (-0.03, 0.65) 74.31
2
Xu (2021) 0.97 (0.38, 1.56) 25.69
Subgroup, DL (I^2 = 100.0%, p < 0.000) 0.97 (0.38, 1.56) 25.69
Heterogeneity between groups: p = 0.056
Overall, DL (I^2 = 47.3%, p = 0.128) 0.46 (0.04, 0.87) 100.00
-2
0
2
NOTE: Weights and between-subgroup heterogeneity test are from random-effects model

7

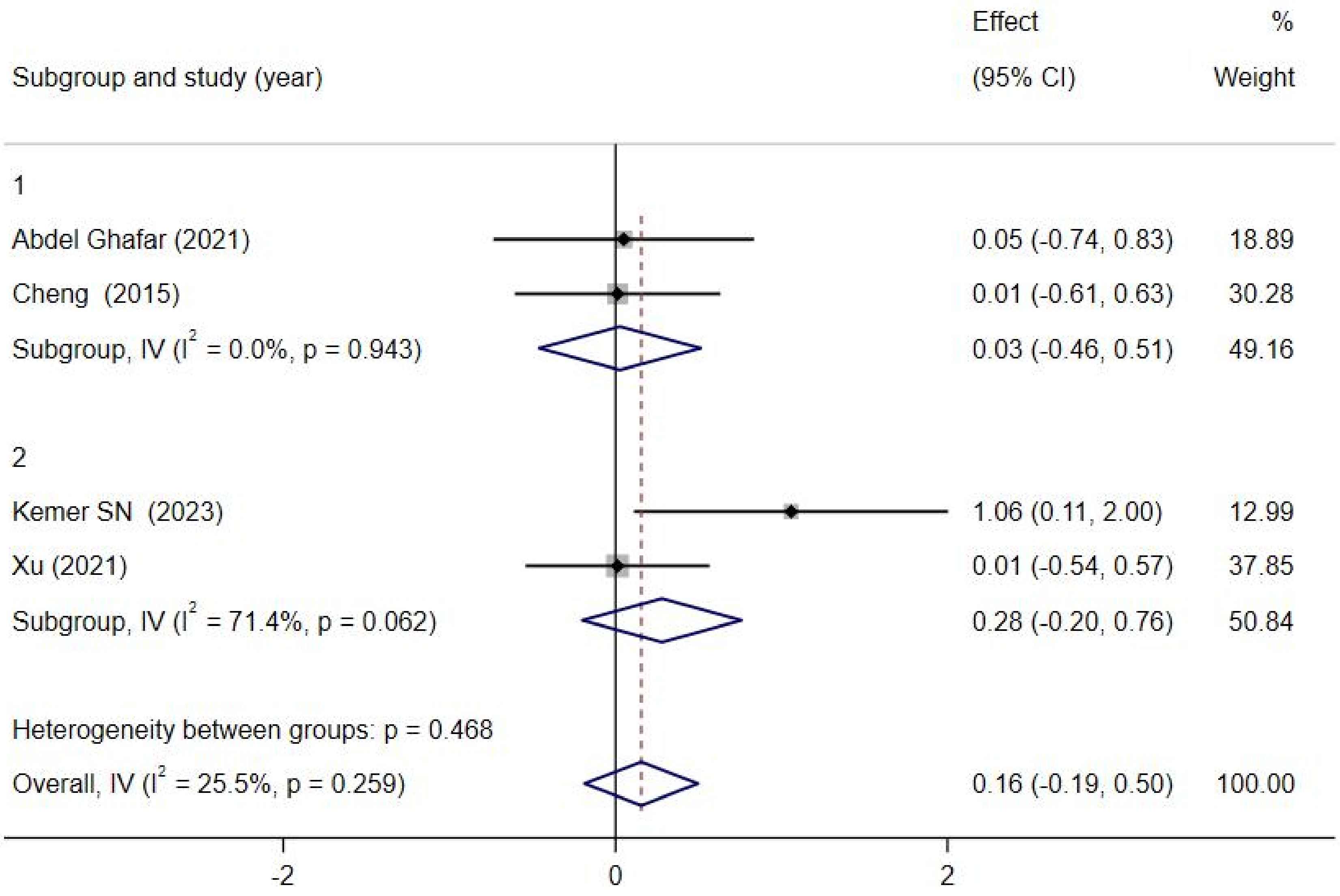
Effect
%
Subgroup and study (year)
(95% CI)
Weight
1
Abdel Ghafar (2021)
0.05 (-0.74, 0.83)
18.89
Cheng (2015)
0.01 (-0.61, 0.63)
30.28
Subgroup, IV (I^2 = 0.0%, p = 0.943)
0.03 (-0.46, 0.51)
49.16
2
Kemer SN (2023)
1.06 (0.11, 2.00)
12.99
Xu (2021)
0.01 (-0.54, 0.57)
37.85
Subgroup, IV (I^2 = 71.4%, p = 0.062)
0.28 (-0.20, 0.76)
50.84
Heterogeneity between groups: p = 0.468
Overall, IV (I^2 = 25.5%, p = 0.259)
0.16 (-0.19, 0.50)
100.00
-2
0
2

Subgroup analysis site of application

1： GMFM-D 2:GMFM-E 3:BBS 4:Muscle Tension-Heel-Ear Test
5: Step speed 6: Step length 7: Step frequency

Over DL (DerSimonian-Laird):
This is an indicator of heterogeneity calculated using the DerSimonian-Laird method.The DL method is a random effects model used to calculate the combined effect sizes and their confidence intervals.

Over IV (Inverse Variance):
This is an indicator of heterogeneity calculated using the inverse variance weighting method.The IV method is a fixed effects model.

I^2 (I-squared) is the percentage of heterogeneity and indicates the proportion of between-stud
y variation to the total variation. p-values were used to test whether the heterogeneity was sig
nificant or not, (usually $p < 0.05$ is considered significant).
