# Supplementary material for: The effect of Kinesio Taping on motor function in children with cerebral palsy: a systematic review and meta-analysis of randomized controlled trials
Source: Front Neurol. 2025 Mar 6;16:1527308. doi: 10.3389/fneur.2025.1527308 (PMC11927513; doi:10.3389/fneur.2025.1527308)
Supplement: SUPPLEMENTARY 4 — Egger’s test. [file Data_Sheet_4.pdf]

1

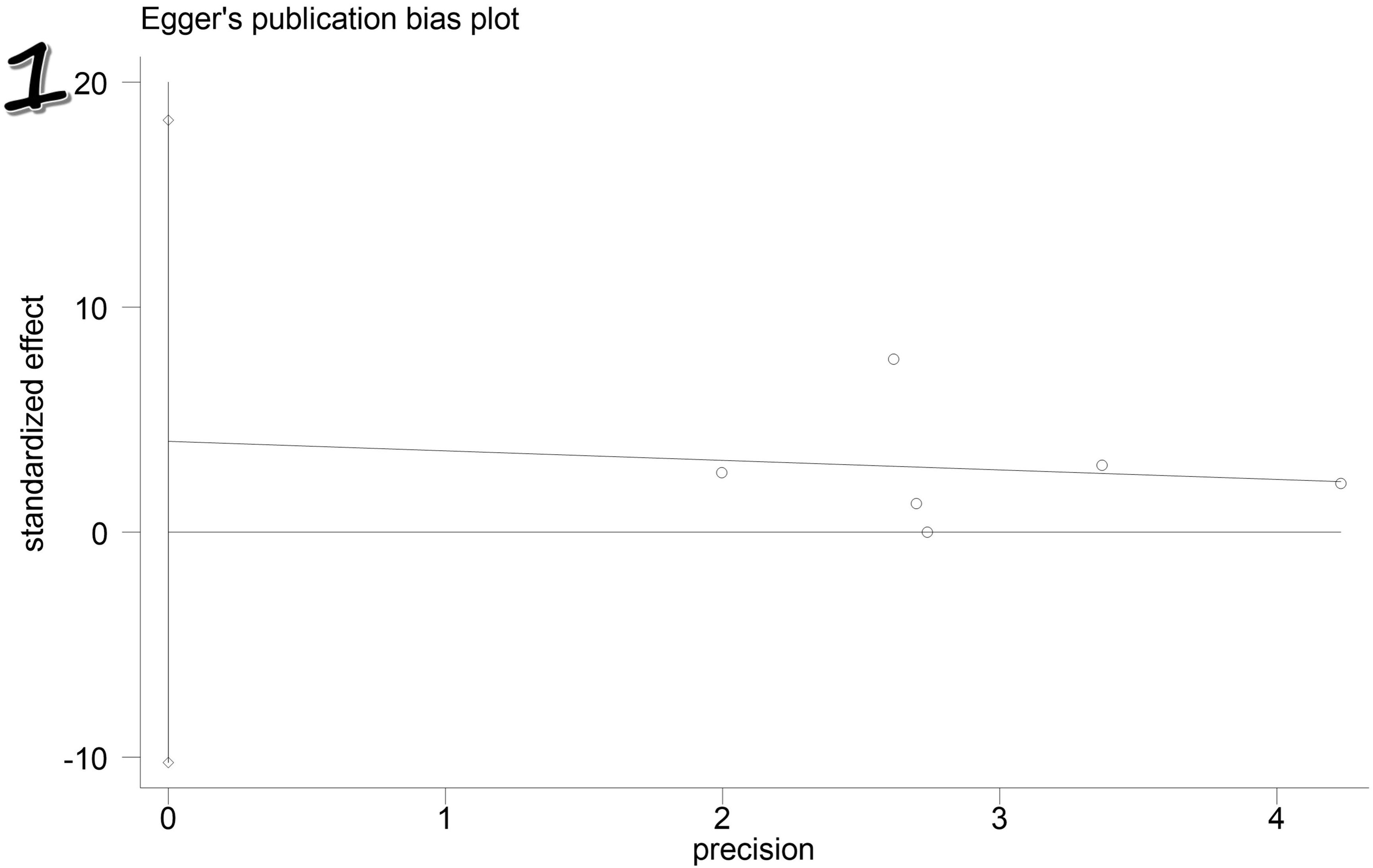

Egger's publication bias plot
standardized effect
20
10
0
-10
0
1
2
3
4
precision

2

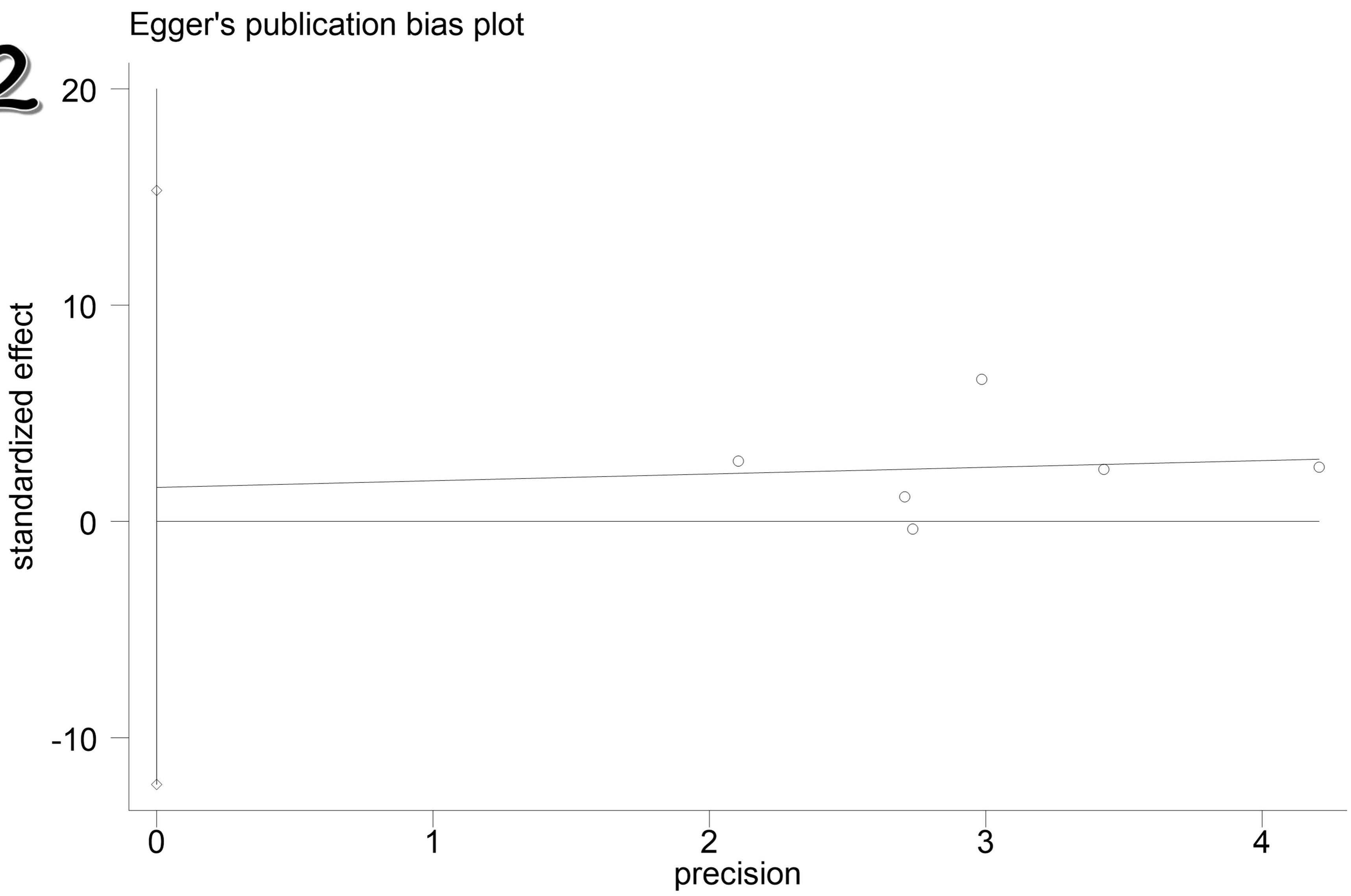

Egger's publication bias plot
20
10
0
-10
standardized effect
0
1
2
3
4
precision

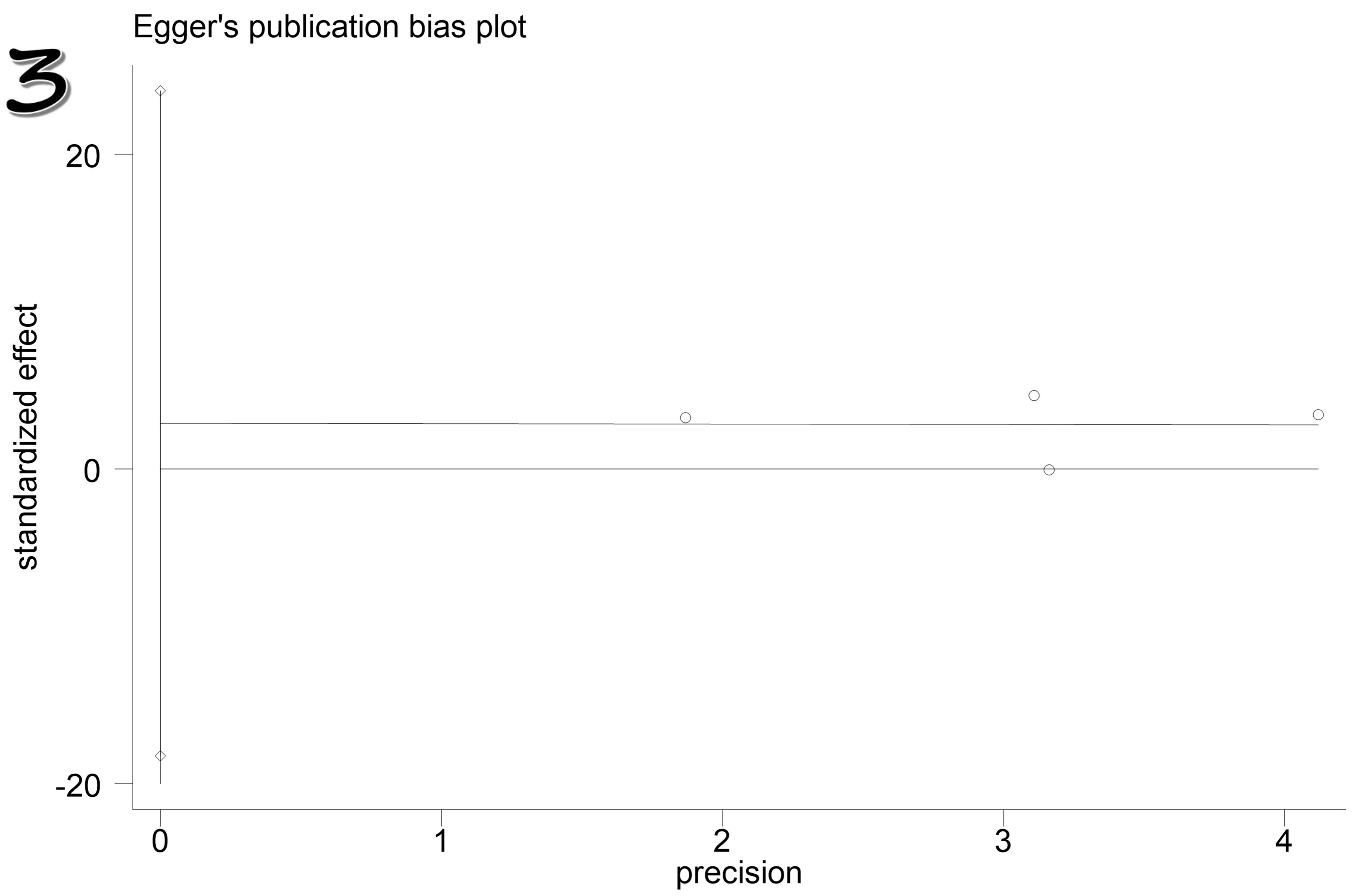

3
Egger's publication bias plot
standardized effect
20
0
-20
0
1
2
3
4
precision

4

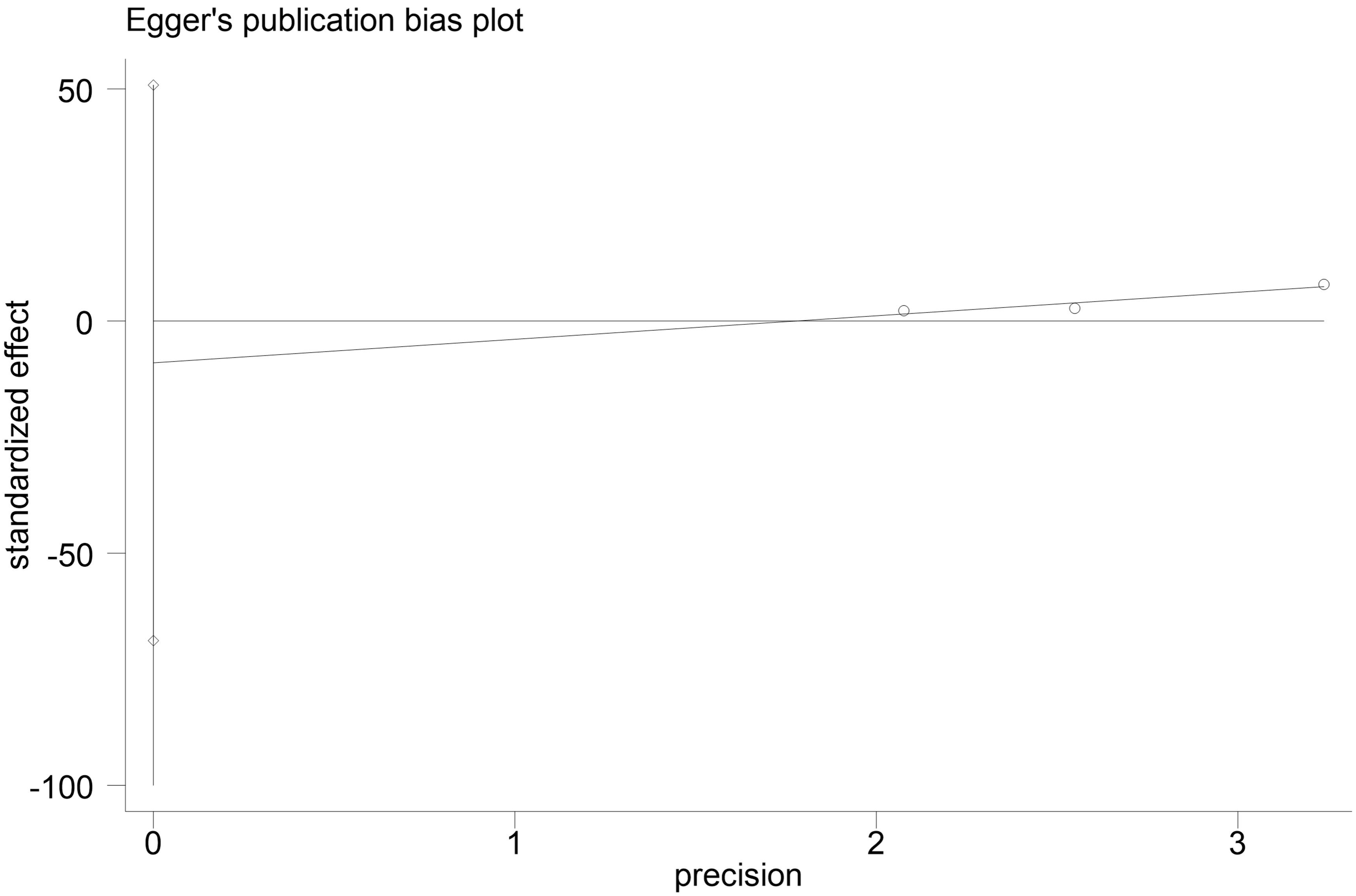

Egger's publication bias plot
50
0
-50
-100
standardized effect
0
1
2
3
precision

5

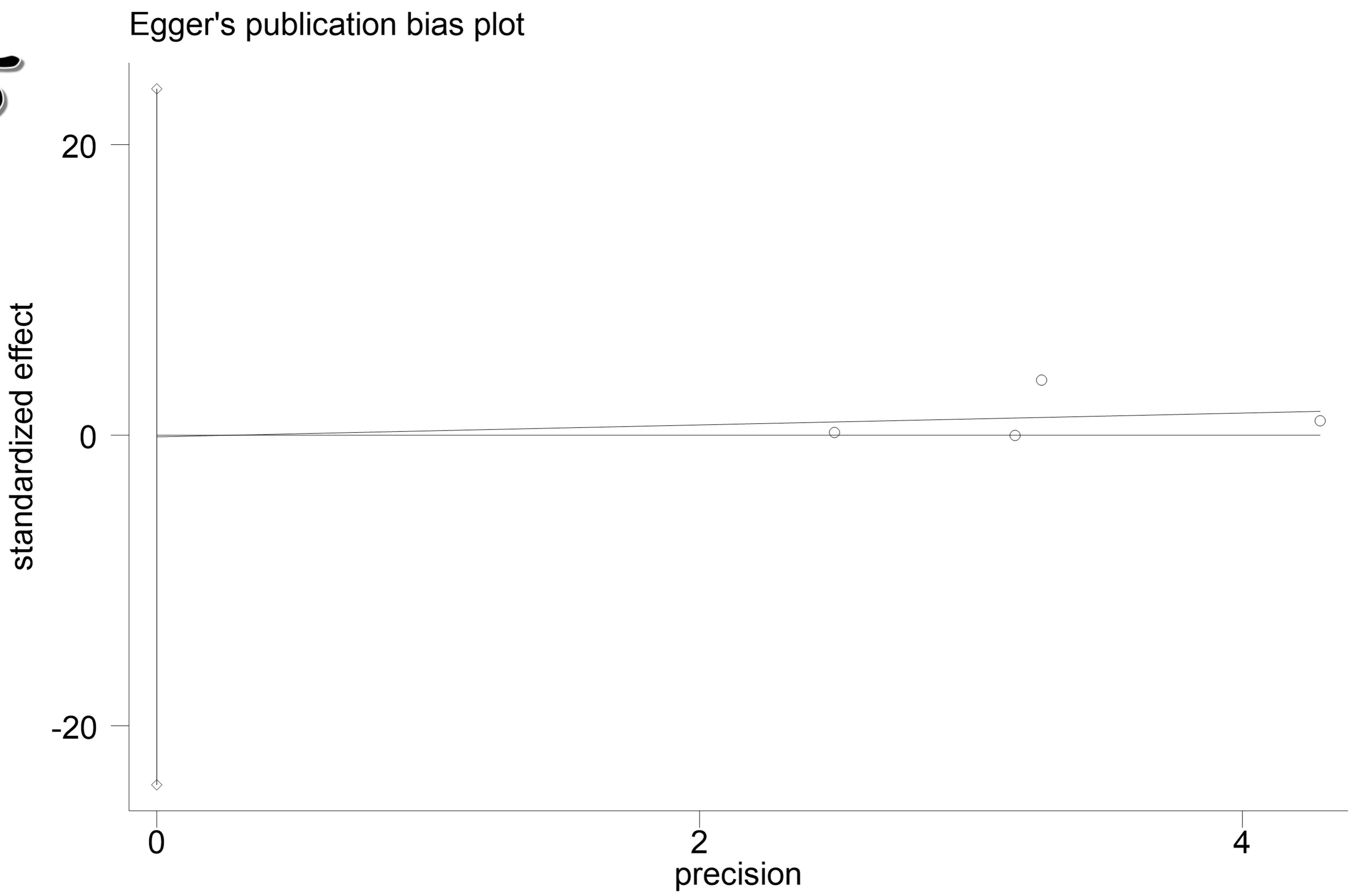
Egger's publication bias plot
20
0
-20
standardized effect
0
2
4
precision

6

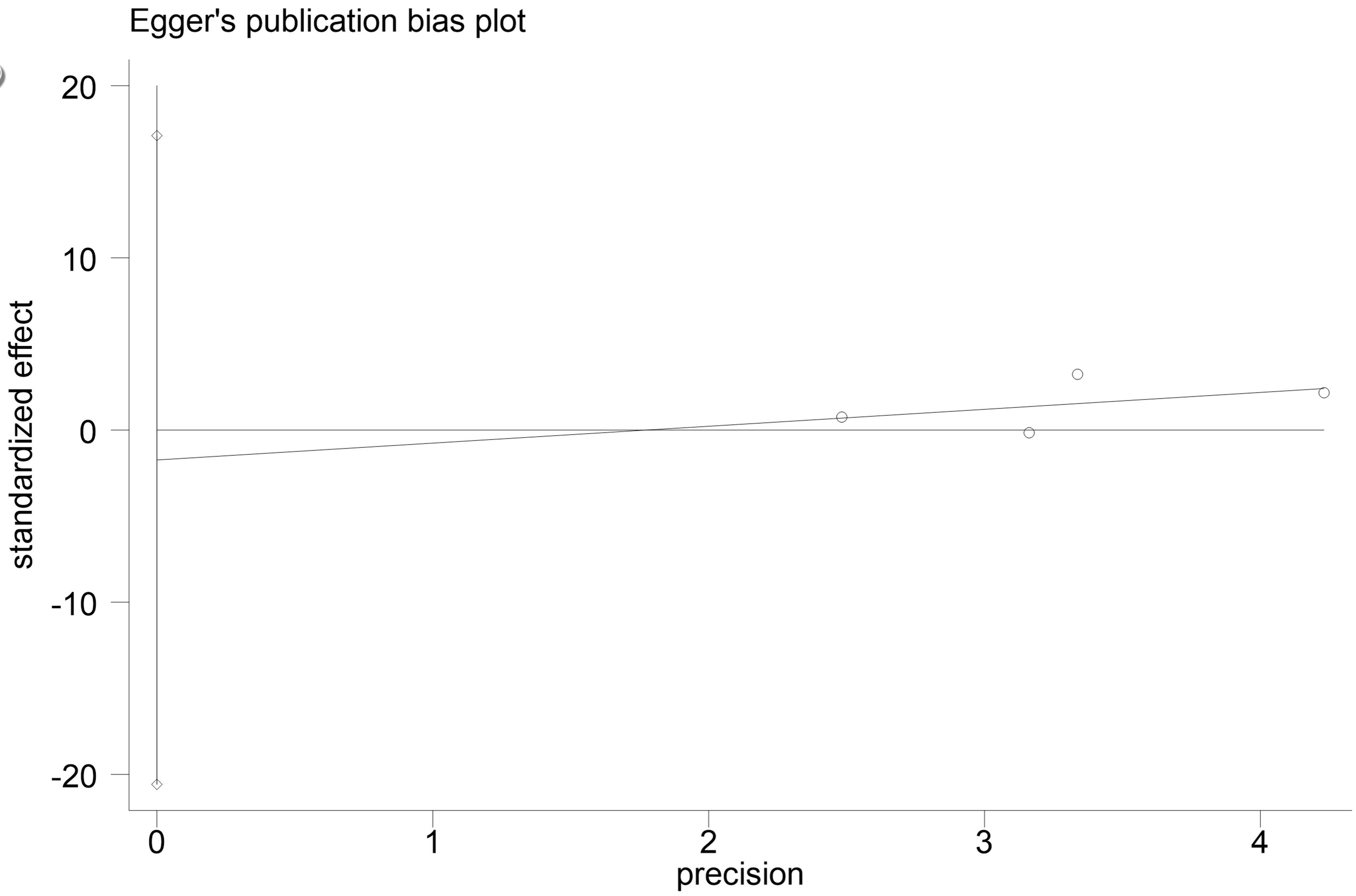

Egger's publication bias plot
standardized effect
precision
20
10
0
-10
-20
0
1
2
3
4

7

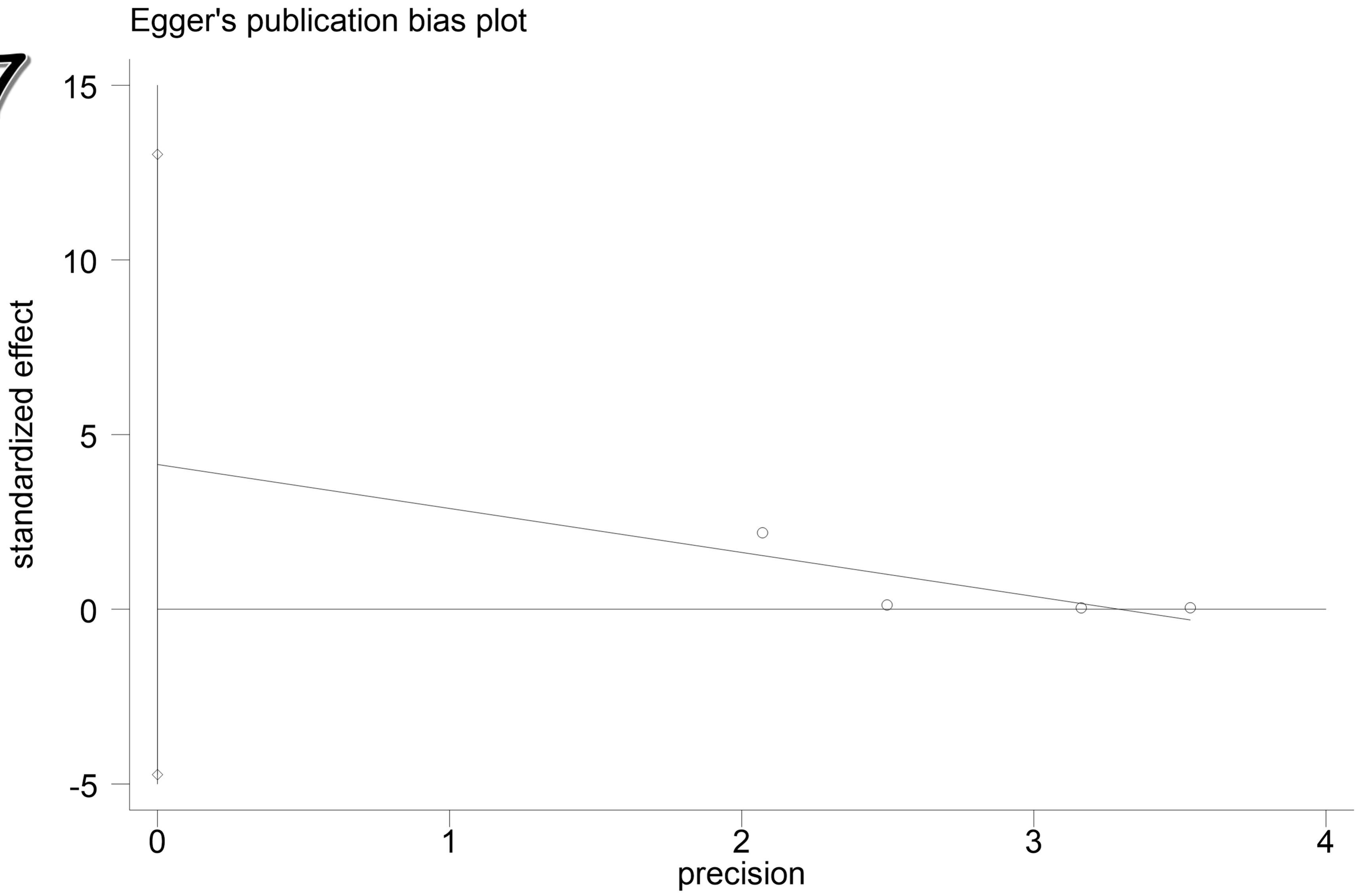
Egger's publication bias plot
15
10
5
0
-5
standardized effect
0
1
2
3
4
precision

Egger's

1:GMFM-D 2:GMFM-E 3:BBS 4:Muscle Tension-Heel-Ear Test

5: Step speed 6: Step length 7: Step frequency
