# Supplementary material for: The effect of Kinesio Taping on motor function in children with cerebral palsy: a systematic review and meta-analysis of randomized controlled trials
Source: Front Neurol. 2025 Mar 6;16:1527308. doi: 10.3389/fneur.2025.1527308 (PMC11927513; doi:10.3389/fneur.2025.1527308)
Supplement: SUPPLEMENTARY 5 — Sensitive analysis. [file Data_Sheet_5.pdf]

1

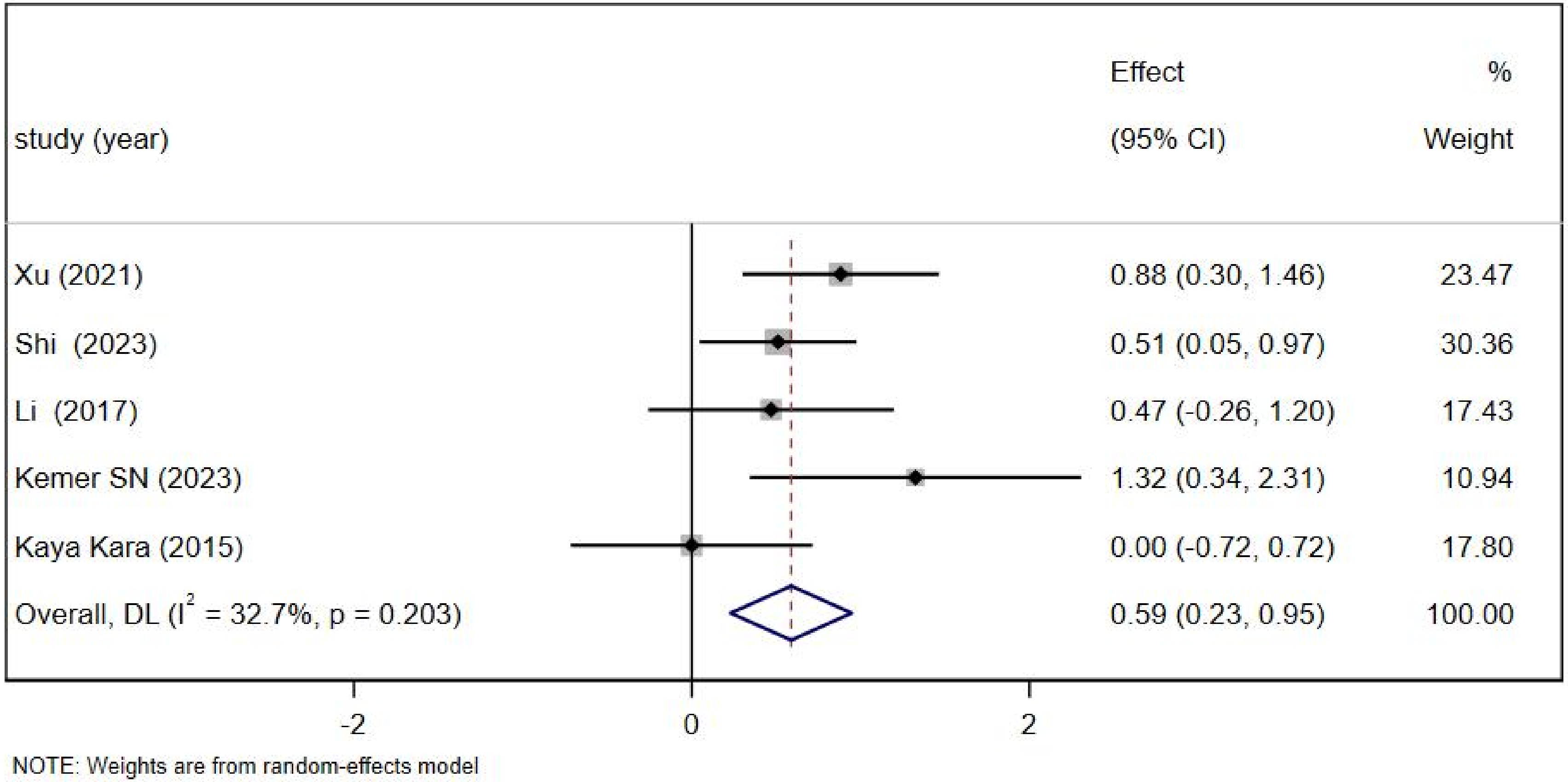

Effect
(95% CI)
%
Weight
study (year)
Xu (2021)
0.88 (0.30, 1.46)
23.47
Shi (2023)
0.51 (0.05, 0.97)
30.36
Li (2017)
0.47 (-0.26, 1.20)
17.43
Kemer SN (2023)
1.32 (0.34, 2.31)
10.94
Kaya Kara (2015)
0.00 (-0.72, 0.72)
17.80
Overall, DL (I^2 = 32.7%, p = 0.203)
0.59 (0.23, 0.95)
100.00
-2
0
2
NOTE: Weights are from random-effects model

2

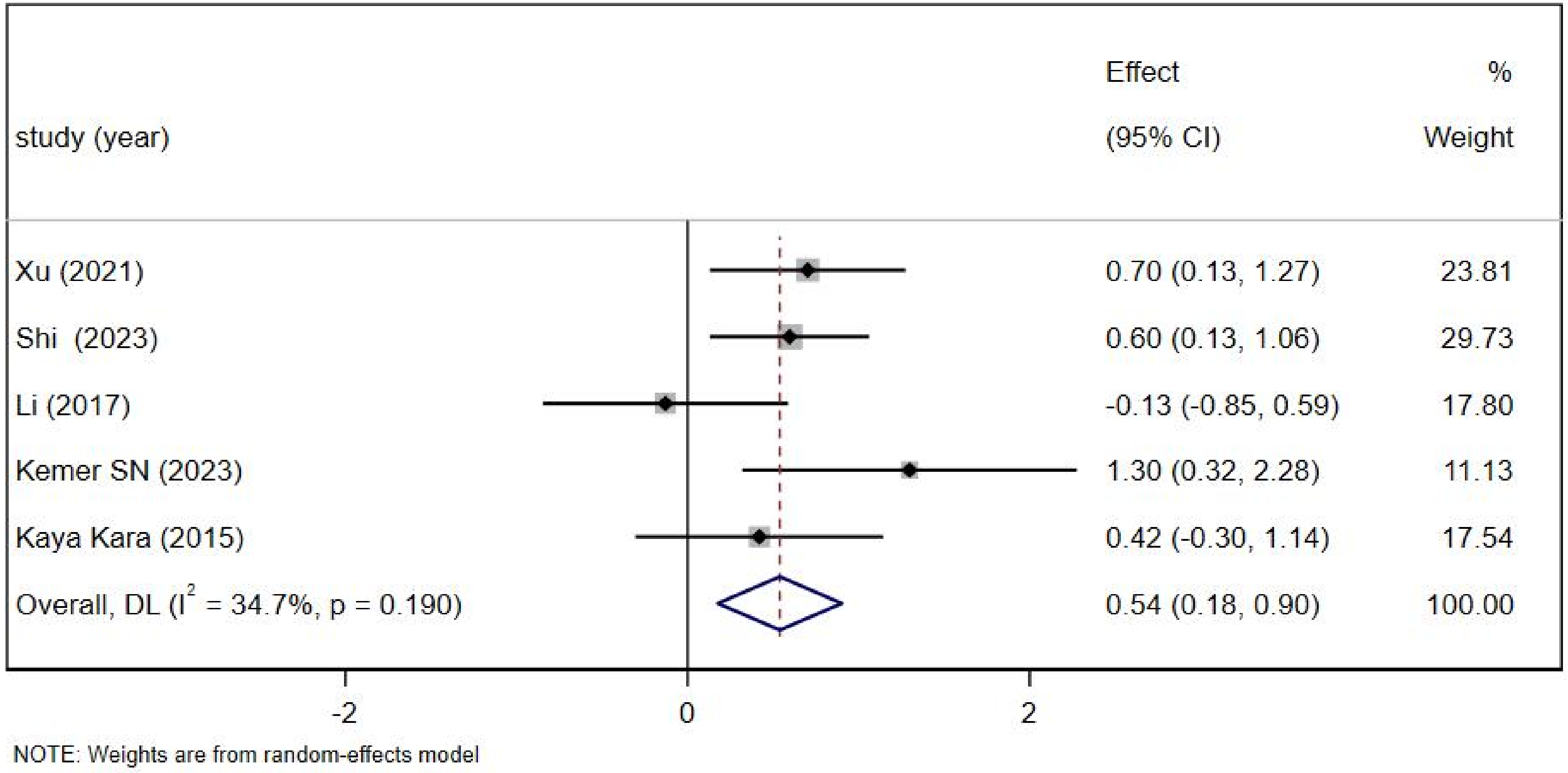

study (year)
Effect
(95% CI)
%
Weight
Xu (2021)
0.70 (0.13, 1.27)
23.81
Shi (2023)
0.60 (0.13, 1.06)
29.73
Li (2017)
-0.13 (-0.85, 0.59)
17.80
Kemer SN (2023)
1.30 (0.32, 2.28)
11.13
Kaya Kara (2015)
0.42 (-0.30, 1.14)
17.54
Overall, DL (I2 = 34.7%, p = 0.190)
0.54 (0.18, 0.90)
100.00
-2
0
2
NOTE: Weights are from random-effects model

3

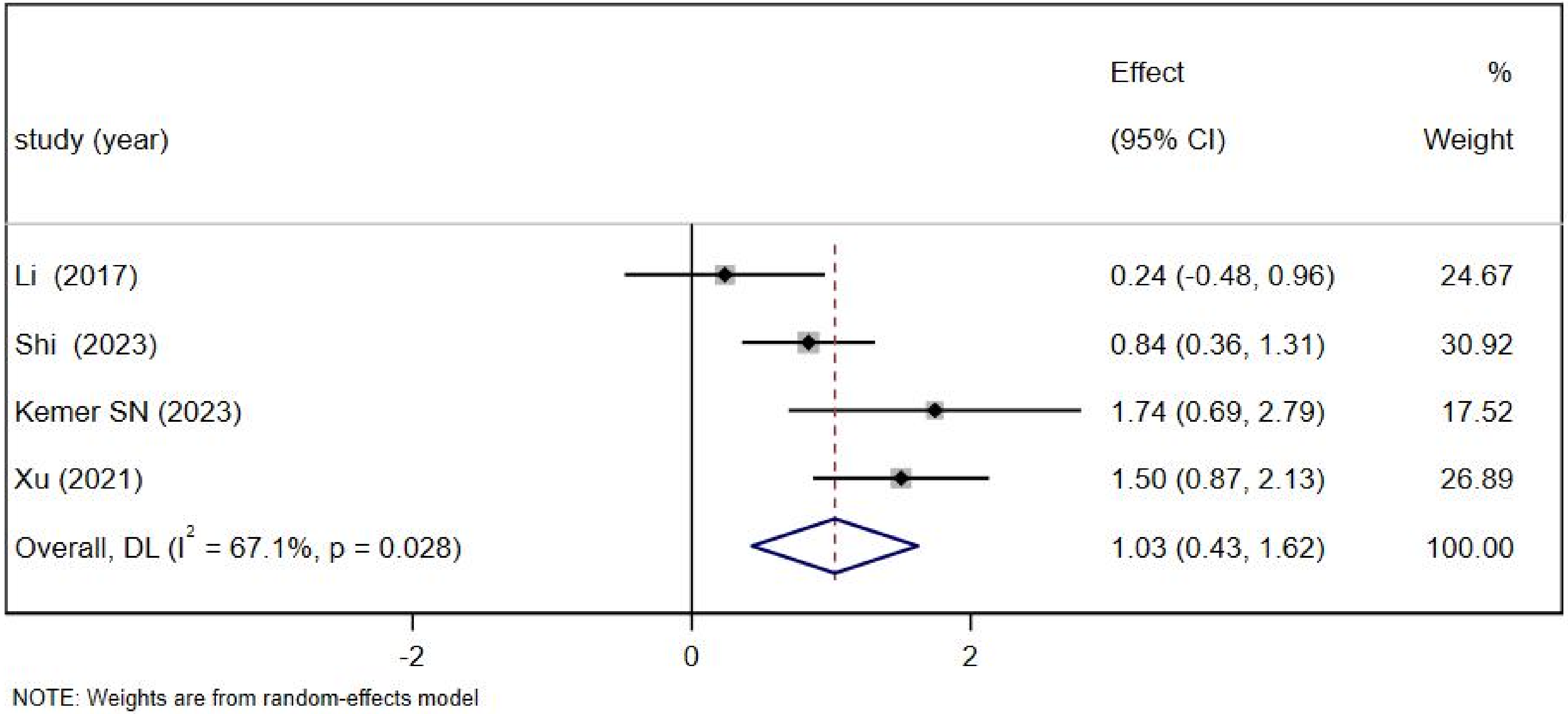

Effect
%
study (year)
(95% CI)
Weight
Li (2017)
0.24 (-0.48, 0.96)
24.67
Shi (2023)
0.84 (0.36, 1.31)
30.92
Kemer SN (2023)
1.74 (0.69, 2.79)
17.52
Xu (2021)
1.50 (0.87, 2.13)
26.89
Overall, DL (I^2 = 67.1%, p = 0.028)
1.03 (0.43, 1.62)
100.00
-2
0
2
NOTE: Weights are from random-effects model

4

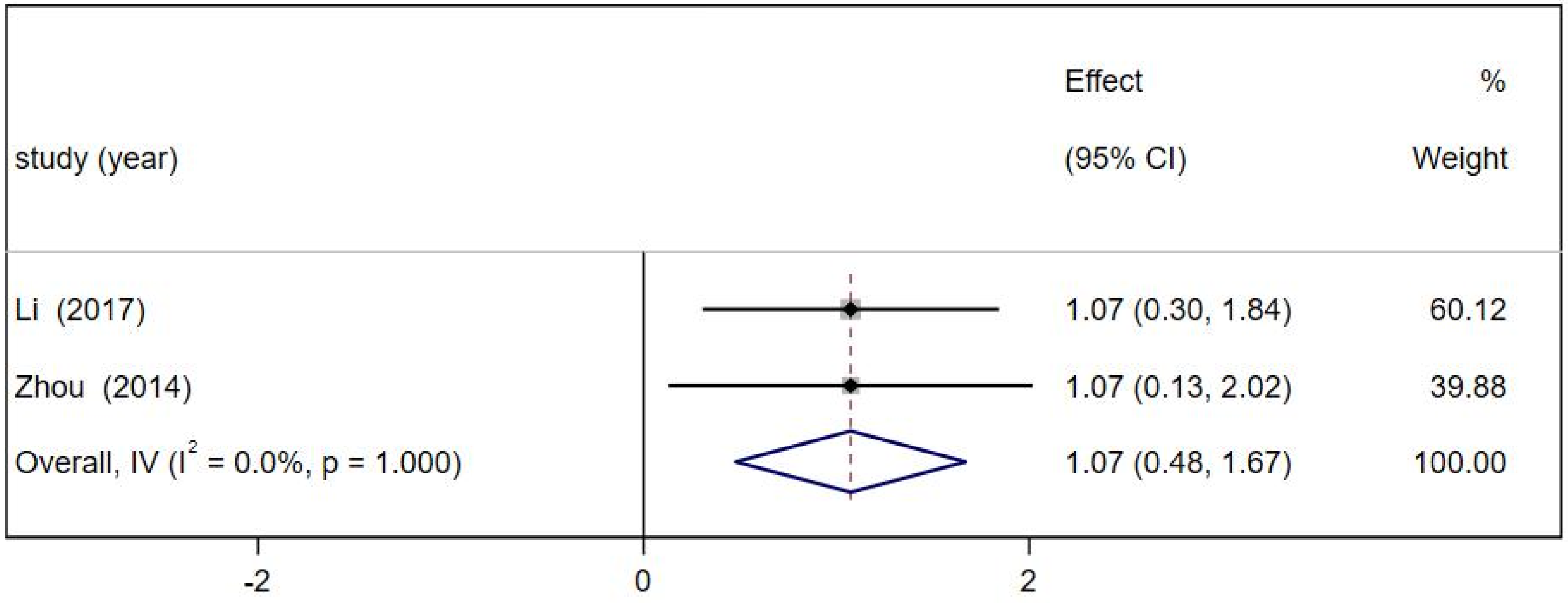
study (year)
Effect
(95% CI)
%
Weight
Li (2017)
1.07 (0.30, 1.84)
60.12
Zhou (2014)
1.07 (0.13, 2.02)
39.88
Overall, IV (I² = 0.0%, p = 1.000)
1.07 (0.48, 1.67)
100.00
-2
0
2

5

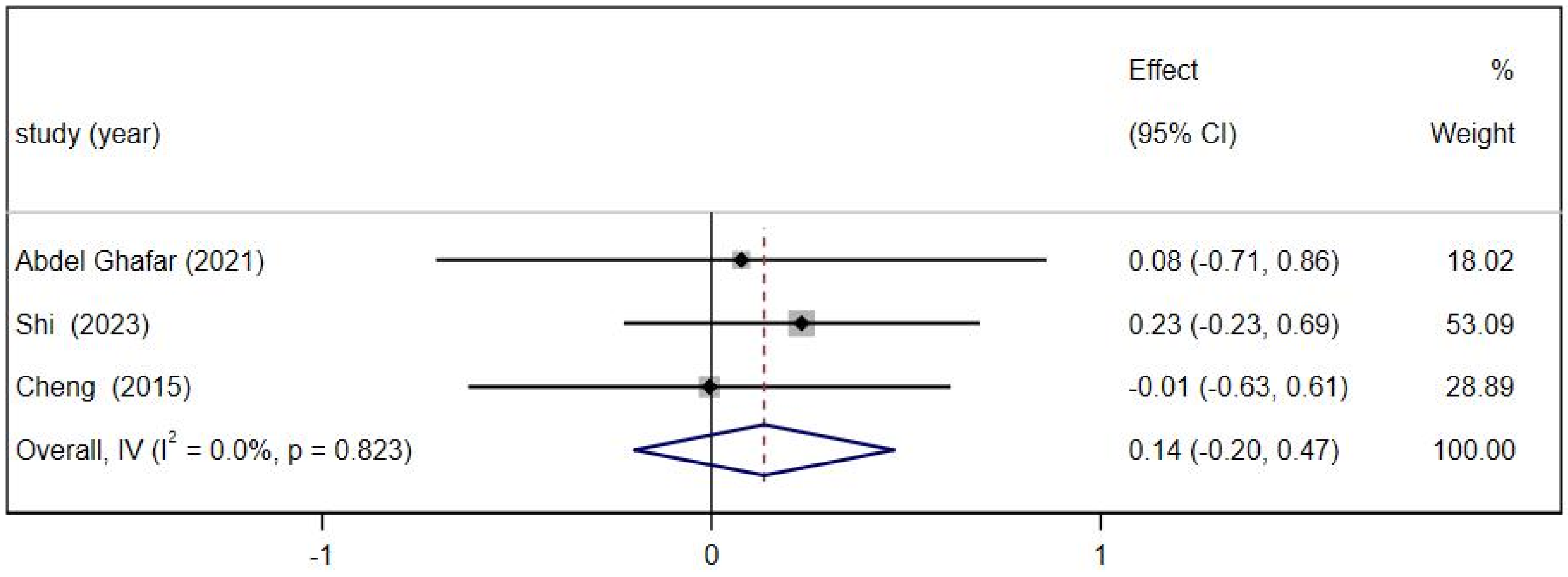

study (year)
Effect
(95% CI)
%
Weight
Abdel Ghafar (2021)
0.08 (-0.71, 0.86)
18.02
Shi (2023)
0.23 (-0.23, 0.69)
53.09
Cheng (2015)
-0.01 (-0.63, 0.61)
28.89
Overall, IV (I2 = 0.0%, p = 0.823)
0.14 (-0.20, 0.47)
100.00
-1
0
1

6

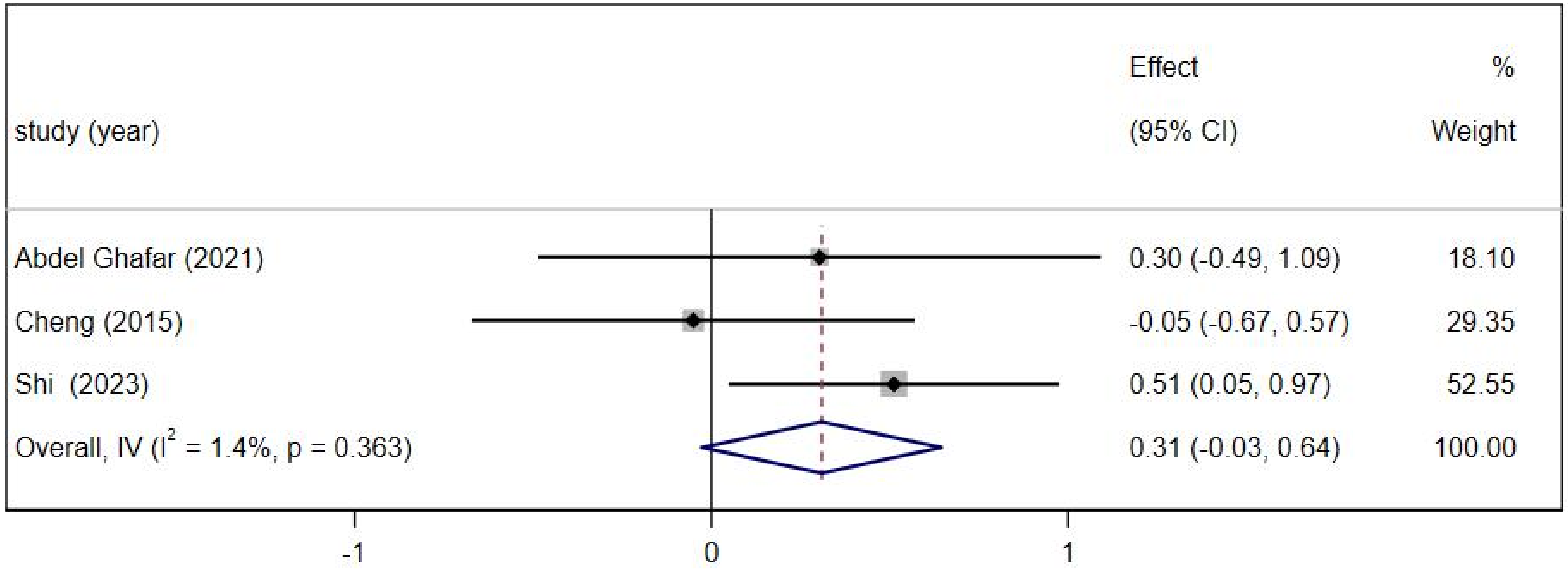
study (year)
Effect
(95% CI)
%
Weight
Abdel Ghafar (2021)
0.30 (-0.49, 1.09)
18.10
Cheng (2015)
-0.05 (-0.67, 0.57)
29.35
Shi (2023)
0.51 (0.05, 0.97)
52.55
Overall, IV (I2 = 1.4%, p = 0.363)
0.31 (-0.03, 0.64)
100.00
-1
0
1

7

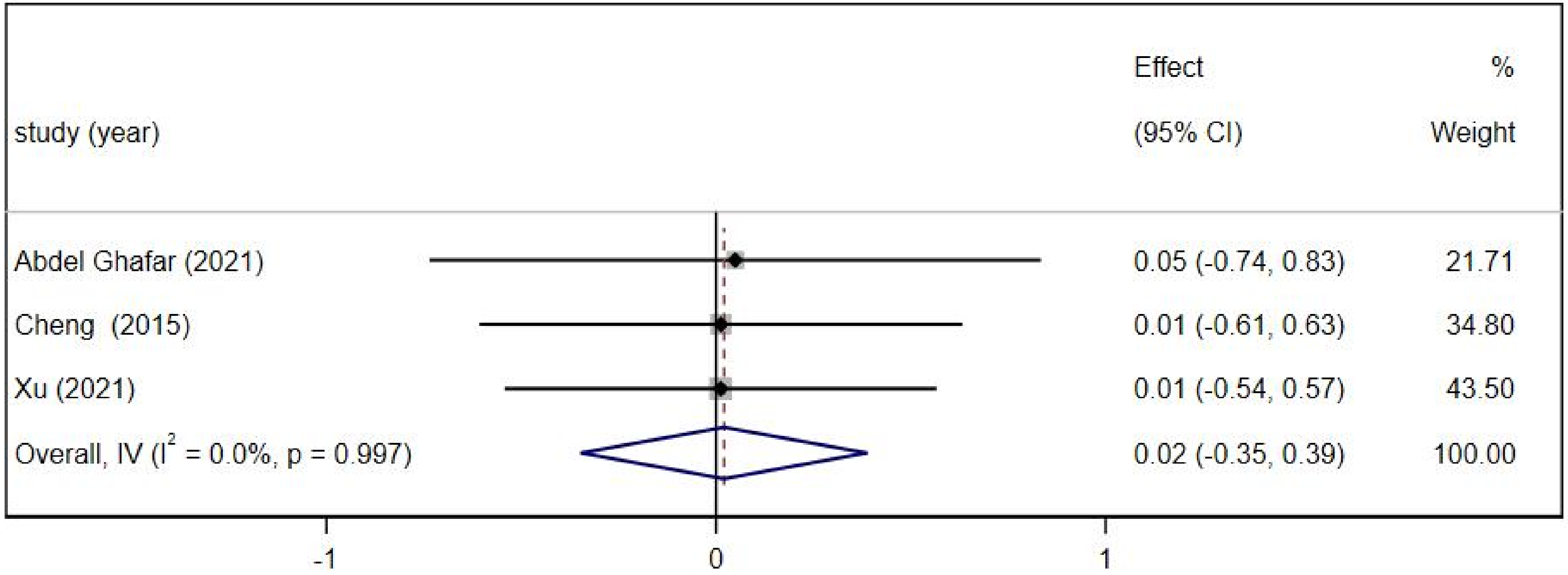
Effect
%
study (year)
(95% CI)
Weight
Abdel Ghafar (2021)
0.05 (-0.74, 0.83)
21.71
Cheng (2015)
0.01 (-0.61, 0.63)
34.80
Xu (2021)
0.01 (-0.54, 0.57)
43.50
Overall, IV (I2 = 0.0%, p = 0.997)
0.02 (-0.35, 0.39)
100.00
-1
0
1

sensitive analysis

1：GMFM-D 2:GMFM-E 3:BBS 4:Muscle Tension-Heel-Ear Test

5: Step speed 6: Step length 7: Step frequency

Over DL (DerSimonian-Laird):
This is an indicator of heterogeneity calculated using the DerSimonian-Laird method.The DL method is a random effects model used to calculate the combined effect sizes and their confidence intervals.

Over IV (Inverse Variance):
This is an indicator of heterogeneity calculated using the inverse variance weighting method.The IV method is a fixed effects model.

I^2 (I-squared) is the percentage of heterogeneity and indicates the proportion of between-stud
y variation to the total variation. p-values were used to test whether the heterogeneity was sig
nificant or not, (usually $p < 0.05$ is considered significant).
