# Supplementary material for: The effect of Kinesio Taping on motor function in children with cerebral palsy: a systematic review and meta-analysis of randomized controlled trials
Source: Front Neurol. 2025 Mar 6;16:1527308. doi: 10.3389/fneur.2025.1527308 (PMC11927513; doi:10.3389/fneur.2025.1527308)
Supplement: SUPPLEMENTARY 6 — Search strategy. [file Data_Sheet_6.pdf]

Pubmed

(("Cerebral Palsy"[MeSH Terms] OR (("cogn process"[Journal] OR "cp"[All Fields]) AND "Cerebral Palsy"[Title/Abstract]) OR (("Cerebral Palsy"[MeSH Terms] OR ("Cerebral"[All Fields] AND "Palsy"[All Fields]) OR "Cerebral Palsy"[All Fields]) AND "Dystonic-Rigid"[Title/Abstract]) OR ((("cerebrally"[All Fields] OR "cerebrum"[MeSH Terms] OR "cerebrum"[All Fields] OR "Cerebral"[All Fields] OR "brain"[MeSH Terms] OR "brain"[All Fields]) AND ("paralysis"[MeSH Terms] OR "paralysis"[All Fields] OR "palsied"[All Fields] OR "Palsies"[All Fields] OR "Palsy"[All Fields])) AND "Dystonic-Rigid"[Title/Abstract]) OR (("Cerebral Palsy"[MeSH Terms] OR ("Cerebral"[All Fields] AND "Palsy"[All Fields]) OR "Cerebral Palsy"[All Fields]) AND "Dystonic-Rigid"[Title/Abstract]) OR ("Dystonic-Rigid"[All Fields] AND "cerebral palsies"[Title/Abstract]) OR ("Dystonic-Rigid"[All Fields] AND "Cerebral Palsy"[Title/Abstract]) OR "cerebral palsy mixed"[Title/Abstract] OR (("Mixed"[All Fields] OR "mixes"[All Fields] OR "mixing"[All Fields] OR "mixings"[All Fields]) AND "cerebral palsies"[Title/Abstract]) OR "mixed cerebral palsy"[Title/Abstract] OR (("Cerebral Palsy"[MeSH Terms] OR ("Cerebral"[All Fields] AND "Palsy"[All Fields]) OR "Cerebral Palsy"[All Fields] OR ("Cerebral"[All Fields] AND "Palsy"[All Fields] AND "Monoplegic"[All Fields])) AND "Infantile"[Title/Abstract]) OR ("Monoplegic"[All Fields] AND "infantile cerebral palsy"[Title/Abstract]) OR (("Cerebral Palsy"[MeSH Terms] OR ("Cerebral"[All Fields] AND "Palsy"[All Fields]) OR "Cerebral Palsy"[All Fields] OR ("Infantile"[All Fields] AND "Cerebral"[All Fields] AND "Palsy"[All Fields]) OR "infantile cerebral palsy"[All Fields]) AND "Monoplegic"[Title/Abstract]) OR (("Cerebral Palsy"[MeSH Terms] OR ("Cerebral"[All Fields] AND "Palsy"[All Fields]) OR "Cerebral Palsy"[All Fields]) AND "quadriplegic infantile"[Title/Abstract]) OR "quadriplegic infantile cerebral palsy"[Title/Abstract] OR (("Cerebral Palsy"[MeSH Terms] OR ("Cerebral"[All Fields] AND "Palsy"[All Fields]) OR "Cerebral Palsy"[All Fields] OR ("Infantile"[All Fields] AND "Cerebral"[All Fields] AND "Palsy"[All Fields]) OR "infantile cerebral palsy"[All Fields]) AND "Quadriplegic"[Title/Abstract]) OR (("Cerebral Palsy"[MeSH Terms] OR ("Cerebral"[All Fields] AND "Palsy"[All Fields]) OR "Cerebral Palsy"[All Fields]) AND "rolandic type"[Title/Abstract]) OR "rolandic type cerebral palsy"[Title/Abstract] OR "cerebral palsy congenital"[Title/Abstract] OR "congenital cerebral palsy"[Title/Abstract] OR "little disease"[Title/Abstract] OR "little s disease"[Title/Abstract] OR "spastic diplegia"[Title/Abstract] OR (("Diplegia"[All Fields] OR "Diplegias"[All Fields]) AND "Spastic"[Title/Abstract]) OR "spastic diplegias"[Title/Abstract] OR "diplegia spastic"[Title/Abstract] OR "monoplegic cerebral palsy"[Title/Abstract] OR ((("cerebrally"[All Fields] OR "cerebrum"[MeSH Terms] OR "cerebrum"[All Fields] OR "Cerebral"[All Fields] OR "brain"[MeSH Terms] OR "brain"[All Fields]) AND ("paralysis"[MeSH Terms] OR "paralysis"[All Fields] OR "palsied"[All Fields] OR "Palsies"[All Fields] OR "Palsy"[All Fields])) AND "Monoplegic"[Title/Abstract]) OR (("Cerebral Palsy"[MeSH Terms] OR ("Cerebral"[All Fields] AND "Palsy"[All Fields]) OR "Cerebral Palsy"[All Fields]) AND "Monoplegic"[Title/Abstract]) OR ("Monoplegic"[All Fields] AND "cerebral palsies"[Title/Abstract]) OR "cerebral palsy athetoid"[Title/Abstract] OR "athetoid cerebral palsy"[Title/Abstract] OR ((("cerebrally"[All Fields] OR "cerebrum"[MeSH Terms] OR "cerebrum"[All Fields] OR "Cerebral"[All Fields] OR "brain"[MeSH Terms] OR "brain"[All

Fields]) AND ("paralysis"[MeSH Terms] OR "paralysis"[All Fields] OR "palsied"[All Fields] OR "Palsies"[All Fields] OR "Palsy"[All Fields])) AND "Athetoid"[Title/Abstract]) OR "cerebral palsy dyskinetic"[Title/Abstract] OR "dyskinetic cerebral palsy"[Title/Abstract] OR ((("cerebrally"[All Fields] OR "cerebrum"[MeSH Terms] OR "cerebrum"[All Fields] OR "Cerebral"[All Fields] OR "brain"[MeSH Terms] OR "brain"[All Fields]) AND ("paralysis"[MeSH Terms] OR "paralysis"[All Fields] OR "palsied"[All Fields] OR "Palsies"[All Fields] OR "Palsy"[All Fields])) AND "Dyskinetic"[Title/Abstract]) OR (("Cerebral Palsy"[MeSH Terms] OR ("Cerebral"[All Fields] AND "Palsy"[All Fields]) OR "Cerebral Palsy"[All Fields]) AND "Atonic"[Title/Abstract]) OR "atonic cerebral palsy"[Title/Abstract] OR "cerebral palsy hypotonic"[Title/Abstract] OR (("Hypotonic"[All Fields] OR "hypotonically"[All Fields] OR "hypotonicity"[All Fields] OR "hypotonics"[All Fields]) AND "cerebral palsies"[Title/Abstract]) OR "hypotonic cerebral palsy"[Title/Abstract] OR ((("Cerebral Palsy"[MeSH Terms] OR ("Cerebral"[All Fields] AND "Palsy"[All Fields]) OR "Cerebral Palsy"[All Fields]) AND ("Diplegic"[All Fields] OR "diplegics"[All Fields])) AND "Infantile"[Title/Abstract]) OR (("Diplegic"[All Fields] OR "diplegics"[All Fields]) AND "infantile cerebral palsy"[Title/Abstract]) OR (("Infantile"[All Fields] OR "infantiles"[All Fields]) AND "cerebral palsy diplegic"[Title/Abstract]) OR "cerebral palsy spastic"[Title/Abstract] OR (("muscle spasticity"[MeSH Terms] OR ("muscle"[All Fields] AND "spasticity"[All Fields]) OR "muscle spasticity"[All Fields] OR "Spastic"[All Fields] OR "spasticity"[All Fields] OR "spastics"[All Fields] OR "spasticities"[All Fields]) AND "cerebral palsies"[Title/Abstract]) OR "spastic cerebral palsy"[Title/Abstract]) AND ("Athletic Tape"[MeSH Terms] OR "tape athletic"[Title/Abstract] OR "orthotic tape"[Title/Abstract] OR ("Tape"[All Fields] AND "Orthotic"[Title/Abstract]) OR "kinesio tape"[Title/Abstract] OR "kinesio tapes"[Title/Abstract] OR "tape kinesio"[Title/Abstract] OR "tapes kinesio"[Title/Abstract] OR "Kinesiotape"[Title/Abstract])) AND (meta-analysis[Filter] OR randomizedcontrolledtrial[Filter])

EMBASE

Embase
Session Results
....

No.	Query	Results	Date
#2.	#1 AND 'randomized controlled trial'/de	11	28 Mar 2024
#1.	(('elastic therapeutic taping':ab,ti OR 'elastic	47	28 Mar 2024

therapeutic taping method': ab,ti OR

'k-taping': ab,ti OR 'kinesio tape application': ab,ti OR 'kinesio tape method':ab,ti OR 'kinesio tape technique': ab,ti OR 'kinesio taping application': ab,ti OR 'kinesio taping method': ab,ti OR 'kinesio taping technique':ab,ti OR

'kinesiology tape application': ab,ti OR 'kinesiology taping': ab,ti) AND 'k taping':ab,ti OR 'kinesiology taping application': ab,ti OR 'kinesiology taping method': ab,ti OR 'kinesiology taping technique': ab,ti OR 'kinesiotape application': ab,ti OR 'kinesiotape technique': ab,ti OR 'kinesiotaping':ab,ti OR 'kinesiotaping application': ab,ti OR 'kinesiotaping method':a b,ti OR 'kinesiotaping technique': ab,ti OR 'kinesio taping':ab,ti) AND ('brain palsy': ab,ti OR 'brain paralysis':ab,ti OR 'central palsy': ab,ti OR 'central paralysis': ab,ti OR 'cerebral paralysis':ab,ti OR 'cerebral paresis': ab,ti OR 'diplegia spastica': ab,ti OR 'encephalopathia infantilis': ab,ti OR 'palsy, cerebral':ab,ti OR 'spastic diplegia': ab,ti OR 'cerebral palsy': ab,ti)

Cochrane Library

Search Name:
Date Run: 28/03/2024 14:40:10
Comment:

ID Search Hits
#1 Diplegic Infantile Cerebral Palsy 1
#2 (Cerebral Palsy, Diplegic, Infantile):ti,ab,kw OR (Infantile Cerebral Palsy, Diplegic):ti,ab,kw OR (Hypotonic Cerebral Palsies):ti,ab,kw OR (Cerebral Palsy, Hypotonic):ti,ab,kw OR (Hypotonic Cerebral Palsy):ti,ab,kw 8
#3 (Cerebral Palsy, Atonic):ti,ab,kw OR (Atonic Cerebral Palsy):ti,ab,kw OR (Rolandic Type Cerebral Palsy):ti,ab,kw OR (Cerebral Palsy, Rolandic Type):ti,ab,kw OR (CP (Cerebral Palsy)):ti,ab,kw 1805
#4 (Cerebral Palsy, Mixed):ti,ab,kw OR (Mixed Cerebral Palsies):ti,ab,kw OR (Mixed Cerebral Palsy):ti,ab,kw OR (Infantile Cerebral Palsy, Monoplegic):ti,ab,kw OR (Cerebral Palsy, Monoplegic, Infantile):ti,ab,kw 187
#5 (Monoplegic Infantile Cerebral Palsy):ti,ab,kw OR (Spastic Cerebral Palsy):ti,ab,kw OR (Spastic Cerebral Palsies):ti,ab,kw OR (Cerebral Palsy, Spastic):ti,ab,kw OR (Cerebral Palsies, Athetoid):ti,ab,kw 1344
#6 (Cerebral Palsy, Athetoid):ti,ab,kw OR (Dyskinetic Cerebral Palsy):ti,ab,kw OR (Cerebral Palsies, Dyskinetic):ti,ab,kw OR (Athetoid Cerebral Palsy):ti,ab,kw OR (Cerebral Palsy, Dyskinetic):ti,ab,kw 56
#7 (Quadriplegic Infantile Cerebral Palsy):ti,ab,kw OR (Cerebral Palsy, Quadriplegic, Infantile):ti,ab,kw OR (Infantile Cerebral Palsy, Quadriplegic):ti,ab,kw OR (Little Disease):ti,ab,kw OR (Little's Disease):ti,ab,kw 9307
#8 (Spastic Diplegias):ti,ab,kw OR (Spastic Diplegia):ti,ab,kw OR (Diplegias, Spastic):ti,ab,kw OR (Diplegia, Spastic):ti,ab,kw OR (Cerebral Palsies,

Monoplegic):ti,ab,kw216
#9 (Monoplegic Cerebral Palsy):ti,ab,kw OR (Cerebral Palsy, Monoplegic):ti,ab,kw OR (Monoplegic Cerebral Palsies):ti,ab,kw OR (Cerebral Palsy, Dystonic Rigid):ti,ab,kw OR (Cerebral Palsies, Dystonic-Rigid):ti,ab,kw 4
#10 (Dystonic-Rigid Cerebral Palsies):ti,ab,kw OR (Cerebral Palsy, Dystonic-Rigid):ti,ab,kw OR (Dystonic-Rigid Cerebral Palsy):ti,ab,kw OR (Cerebral Palsy, Congenital):ti,ab,kw OR (Congenital Cerebral Palsy):ti,ab,kw 138
#11 (Tape, Kinesio):ti,ab,kw OR (Kinesiotape):ti,ab,kw OR (Kinesio Tapes):ti,ab,kw OR (Tapes, Kinesio):ti,ab,kw OR (Kinesio Tape):ti,ab,kw 858
#12 (Orthotic Tape):ti,ab,kw OR (Tape, Orthotic):ti,ab,kw OR (Tape, Athletic):ti,ab,kw OR (Kinesio taping):ti,ab,kw 1374
#13 #1 OR #2 OR #3 OR #4 OR #5 OR #6 OR #7 OR #8 OR #9 OR #10 12091
#14 #11 OR #12 1564
#15 #13 AND #14 39

Wos

Kinesio taping* AND Cerebral Palsy*

Cnki，Vip，Wan Fang

Kinesio taping AND Cerebral Palsy
