# Supplementary material for: The effect of Kinesio Taping on motor function in children with cerebral palsy: a systematic review and meta-analysis of randomized controlled trials
Source: Front Neurol. 2025 Mar 6;16:1527308. doi: 10.3389/fneur.2025.1527308 (PMC11927513; doi:10.3389/fneur.2025.1527308)
Supplement: SUPPLEMENTARY 8 — Risk of bias. [file Data_Sheet_8.pdf]

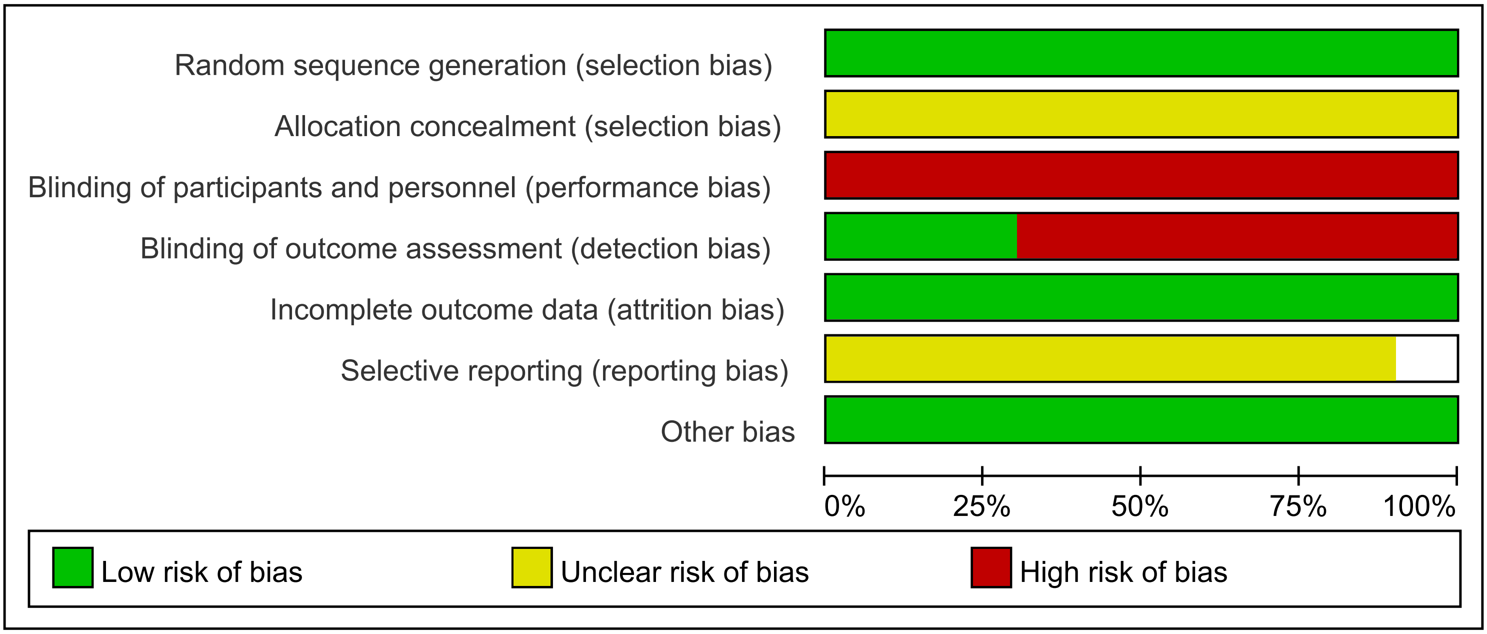

Random sequence generation (selection bias)
Allocation concealment (selection bias)
Blinding of participants and personnel (performance bias)
Blinding of outcome assessment (detection bias)
Incomplete outcome data (attrition bias)
Selective reporting (reporting bias)
Other bias
0%
25%
50%
75%
100%
Low risk of bias
Unclear risk of bias
High risk of bias

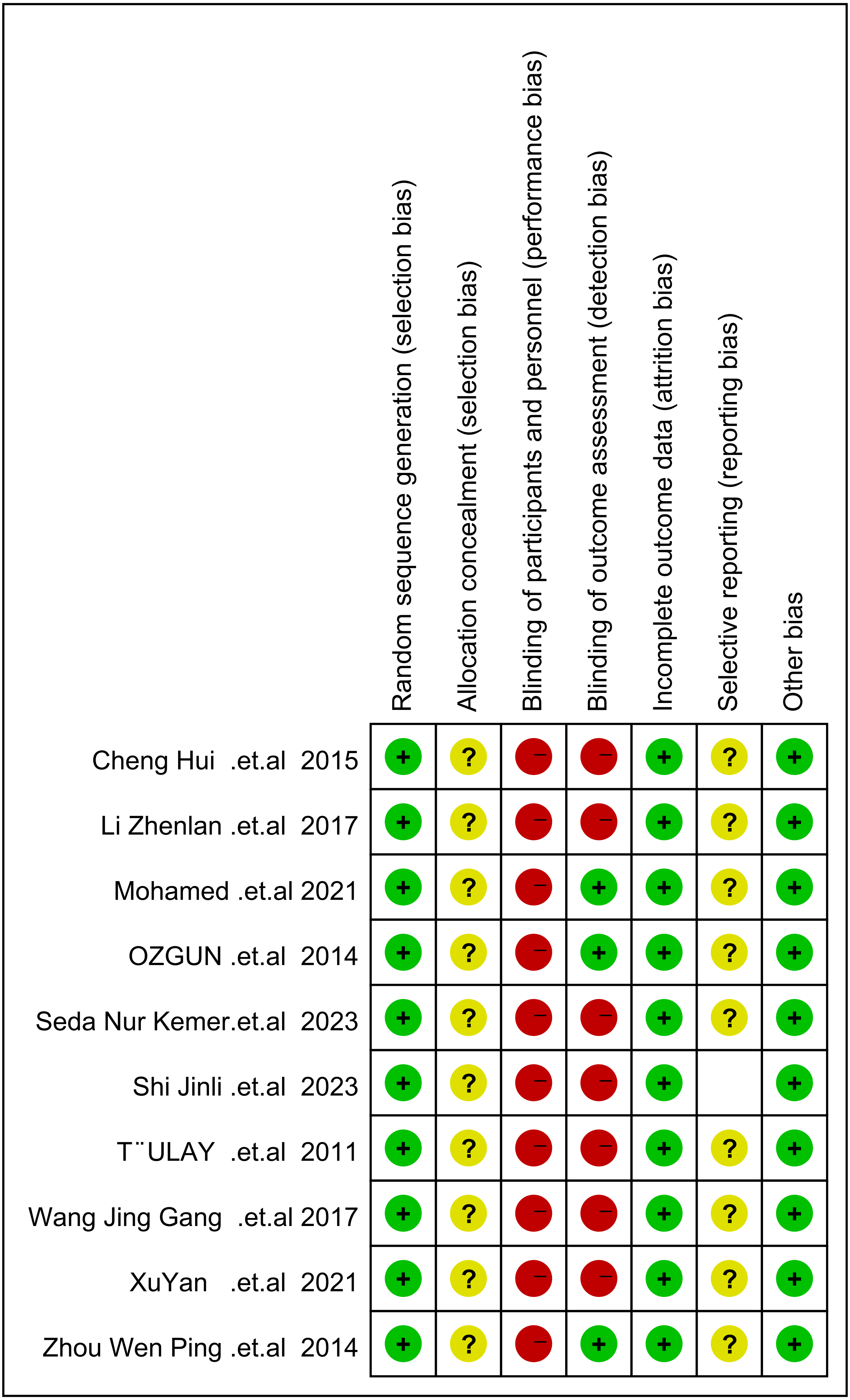
Random sequence generation (selection bias)
Allocation concealment (selection bias)
Blinding of participants and personnel (performance bias)
Blinding of outcome assessment (detection bias)
Incomplete outcome data (attrition bias)
Selective reporting (reporting bias)
Other bias
Cheng Hui .et.al 2015
Li Zhenlan .et.al 2017
Mohamed .et.al 2021
OZGUN .et.al 2014
Seda Nur Kemer.et.al 2023
Shi Jinli .et.al 2023
T¨ULAY .et.al 2011
Wang Jing Gang .et.al 2017
XuYan .et.al 2021
Zhou Wen Ping .et.al 2014
